# Variables Associated With Intravenous Rehydration and Hospitalization in Children With Acute Gastroenteritis: A Secondary Analysis of 2 Randomized Clinical Trials

**DOI:** 10.1001/jamanetworkopen.2021.6433

**Published:** 2021-04-19

**Authors:** Naveen Poonai, Elizabeth C. Powell, David Schnadower, T. Charles Casper, Cindy G. Roskind, Cody S. Olsen, Philip Tarr, Prashant Mahajan, Alexander J. Rogers, Suzanne Schuh, Katrina F. Hurley, Serge Gouin, Cheryl Vance, Ken J. Farion, Robert E. Sapien, Karen J. O’Connell, Adam C. Levine, Seema Bhatt, Stephen B. Freedman

**Affiliations:** 1 Department of Pediatrics, Schulich School of Medicine and Dentistry, London, Canada; 2 Department of Internal Medicine, Schulich School of Medicine and Dentistry, London, Canada; 3 Department of Epidemiology & Biostatistics, Schulich School of Medicine and Dentistry, London, Canada; 4 Children's Health Research Institute, London Health Sciences Centre, London, Canada; 5 Division of Emergency Medicine, Department of Pediatrics, Ann & Robert H. Lurie Children's Hospital of Chicago, Chicago, Illinois; 6 Division of Emergency Medicine, Cincinnati Children’s Hospital Medical Center, Cincinnati, Ohio; 7 Department of Pediatrics, University of Utah, Salt Lake City; 8 Department of Emergency Medicine, Columbia University College of Physicians & Surgeons, New York, New York; 9 Department of Pediatrics, University of Utah, Salt Lake City; 10 Division of Gastroenterology, Hepatology, & Nutrition, Department of Pediatrics, Washington University in St Louis School of Medicine, St Louis, Missouri; 11 Division of Emergency Medicine, Department of Pediatrics, Children’s Hospital of Michigan, Detroit; 12 Wayne State University, Detroit, Michigan; 13 Department of Emergency Medicine, University of Michigan, Ann Arbor; 14 Division of Pediatric Emergency Medicine, The Hospital for Sick Children, SickKids Research Institute, Department of Pediatrics, University of Toronto, Toronto, Ontario, Canada; 15 Department of Emergency Medicine, IWK Health Centre, Halifax, Nova Scotia, Canada; 16 Department of Pediatric Emergency Medicine, Université de Montréal, Montréal, Quebec, Canada; 17 Department of Pediatrics, Université de Montréal, Montréal, Quebec, Canada; 18 Department of Pediatrics, University of California, Davis, School of Medicine, Sacramento; 19 Department of Emergency Medicine, University of California, Davis, School of Medicine, Sacramento; 20 Department of Pediatrics, University of Ottawa, Ottawa, Ontario, Canada; 21 Department of Emergency Medicine, University of Ottawa, Ottawa, Ontario, Canada; 22 Pediatric Emergency Department, Children’s Hospital of Eastern Ontario, Ottawa, Ontario, Canada; 23 Department of Emergency Medicine, University of New Mexico, Albuquerque; 24 Division of Emergency Medicine, Children's National Hospital, Department of Pediatrics, The George Washington University School of Medicine and Health Sciences, Washington, DC; 25 Department of Emergency Medicine, Rhode Island Hospital/Hasbro Children's Hospital and Brown University, Providence; 26 Division of Emergency Medicine, Cincinnati Children's Hospital Medical Center, Department of Pediatrics, University of Cincinnati College of Medicine, Cincinnati, Ohio; 27 Sections of Pediatric Emergency Medicine and Gastroenterology, Department of Pediatric Medicine, Alberta Children’s Hospital, Alberta, Canada; 28 Section of Pediatric Emergency Medicine, Department of Emergency Medicine, Alberta Children’s Hospital, Alberta, Canada; 29 Alberta Children’s Hospital Research Institute, Cumming School of Medicine, University of Calgary, Calgary, Alberta, Canada

## Abstract

**Importance:**

Despite guidelines endorsing oral rehydration therapy, intravenous fluids are commonly administered to children with acute gastroenteritis in high-income countries.

**Objective:**

To identify factors associated with intravenous fluid administration and hospitalization in children with acute gastroenteritis.

**Design, Setting, and Participants:**

This study is a planned secondary analysis of the Pediatric Emergency Research Canada (PERC) and Pediatric Emergency Care Applied Research Network (PECARN) probiotic trials. Participants include children aged 3 to 48 months with 3 or more watery stools in 24 hours between November 5, 2013, and April 7, 2017, for the PERC study and July 8, 2014, and June 23, 2017, for the PECARN Study. Children were from 16 pediatric emergency departments throughout Canada (6) and the US (10). Data were analyzed from November 2, 2018, to March 16, 2021.

**Exposures:**

Sex, age, preceding health care visit, distance between home and hospital, country (US vs Canada), frequency and duration of vomiting and diarrhea, presence of fever, Clinical Dehydration Scale score, oral ondansetron followed by oral rehydration therapy, and infectious agent.

**Main Outcomes and Measures:**

Intravenous fluid administration and hospitalization.

**Results:**

This secondary analysis of 2 randomized clinical trials included 1846 children (mean [SD] age, 19.1 [11.4] months; 1007 boys [54.6%]), of whom 534 of 1846 (28.9%) received oral ondansetron, 240 of 1846 (13.0%) received intravenous rehydration, and 67 of 1846 (3.6%) were hospitalized. The following were independently associated with intravenous rehydration: higher Clinical Dehydration Scale score (mild to moderate vs none, odds ratio [OR], 8.73; 95% CI, 5.81-13.13; and severe vs none, OR, 34.15; 95% CI, 13.45-86.73); country (US vs Canada, OR, 6.76; 95% CI, 3.15-14.49); prior health care visit with intravenous fluids (OR, 4.55; 95% CI, 1.32-15.72); and frequency of vomiting (per 5 episodes, OR, 1.66; 95% CI, 1.39-1.99). The following were independently associated with hospitalization: higher Clinical Dehydration Scale score (mild to moderate vs none, OR, 11.10; 95% CI, 5.05-24.38; and severe vs none, OR, 23.55; 95% CI, 7.09-78.25) and country (US vs Canada, OR, 3.37; 95% CI, 1.36-8.40). Oral ondansetron was associated with reduced odds of intravenous rehydration (OR, 0.21; 95% CI, 0.13-0.32) and hospitalization (OR, 0.44; 95% CI, 0.21-0.89).

**Conclusions and Relevance:**

Intravenous rehydration and hospitalization were associated with clinical evidence of dehydration and lack of an oral ondansetron-supported oral rehydration period. Strategies focusing on oral ondansetron administration followed by oral rehydration therapy in children with dehydration may reduce the reliance on intravenous rehydration and hospitalization.

**Trial Registration:**

ClinicalTrials.gov Identifiers: NCT01853124 (PERC) and NCT01773967 (PECARN)

## Key Points

**Question:**

What clinical features are associated with intravenous rehydration and hospitalization in children with acute gastroenteritis?

**Findings:**

In this secondary analysis of 2 randomized clinical trials with 1846 children, independent variables associated with intravenous rehydration included a higher clinical dehydration score, care in the US relative to Canada, greater frequency and duration of vomiting, prior intravenous rehydration, and lack of oral ondansetron. A higher clinical dehydration score, care in the US, greater frequency of vomiting, and lack of oral ondansetron were associated with hospitalization.

**Meaning:**

These findings suggest that oral ondansetron may support oral rehydration therapy to reduce intravenous rehydration and the hospitalization of children with gastroenteritis.

## Teaser

This secondary analysis of 2 randomized clinical trials investigates which variables are associated with intravenous rehydration and hospitalization in children with acute gastroenteritis.

## Introduction

Acute gastroenteritis (AGE) accounts for nearly 500 000 deaths in children younger than 5 years annually.^1^ Although AGE is generally a mild, self-limited condition in high-income countries, it accounts for almost 1.7 million emergency department (ED) visits^2^ and 60 000 hospitalizations annually in the US.^3^ Guidelines uniformly support oral rehydration therapy (ORT), reserving intravenous rehydration for children with severe dehydration.^4,5,6^ Unfortunately, clinical dehydration scales have variable accuracy,^7^ and most overestimate dehydration severity in high-income countries.^8^ These challenges, combined with the presence of vomiting, the need to minimize ED length of stay, and caregiver expectations,^9^ often lead to intravenous rehydration use.^10^

Intravenous rehydration has potentially deleterious effects. Children rate intravenous insertion as one of the most painful aspects of hospital care,^11^ influencing future reactions to painful events.^12^ Compared with ORT, intravenous rehydration is associated with phlebitis, longer hospital stays, and major adverse events,^13,14^ and is one of the risk factors most strongly associated with ED revisits, presumably because it reinforces the decision to seek ED care and reduces the educational focus on ORT.^15^ Although quality improvement initiatives have been able to reduce intravenous rehydration rates, in many institutions, use remains frequent.^14,16^ Thus, a better understanding of the factors associated with intravenous rehydration is needed to identify approaches to mitigate use.

Reducing unnecessary hospitalizations is also a priority given cost considerations. In 2010, the Agency for Healthcare Research and Quality estimated that the cost of preventable pediatric hospitalizations for AGE in the US was nearly $150 million dollars.^17^ Although rotavirus vaccination has reduced AGE hospitalizations by 36% globally,^18^ this enormous burden continues.^19^ Furthermore, there is considerable variation in hospitalization rates for AGE, and nonobjective measures of dehydration may be an important driver.^20^

To address these issues, we conducted a secondary analysis of 2 large simultaneously collected data sets. Our objective was to explore factors associated with intravenous rehydration and hospitalization in children with AGE in the US and Canada.

## Methods

### Design

This study was a planned secondary analysis of the Pediatric Emergency Research Canada (PERC) Probiotic Regimen for Outpatient Gastroenteritis Utility of Treatment (PROGUT) (trial protocol available in Supplement 1)^21,22^ and Pediatric Emergency Care Applied Research Network (PECARN) (trial protocol available in Supplement 2)^23,24^ randomized clinical trials of probiotics in children with AGE-associated diarrhea. Research assistants at each site obtained written informed consent from the children’s parents. Participants were enrolled between November 5, 2013, and June 23, 2017, in 1 of 16 EDs, 6 in the PERC trial and 10 in the PECARN trial. Research ethics board approval was obtained at each site. The manuscript analysis plan for the present study is available in Supplement 3. This study followed the Strengthening the Reporting of Observational Studies in Epidemiology (STROBE) reporting guideline.

### Participants

Eligible children were aged 3 to 48 months and had 3 or more watery stools reported in the preceding 24 hours. Exclusion criteria were prior enrollment in the study, hematochezia, bilious emesis, chronic gastrointestinal disease, structural heart disease, indwelling vascular access line, immunotherapy or history of immunodeficiency, inability to be contacted for daily follow-up while symptomatic, supplemental probiotic use in the preceding 14 days, clinical instability (eg, hypovolemic shock), family member with an indwelling vascular access line, or being immunocompromised.^22,23^ The main distinctions between the 2 studies were maximal symptom duration before enrollment (72 hours in the PERC trial and 7 days in the PECARN trial) and different investigational probiotic products. In addition, the PERC trial excluded children with pancreatic dysfunction, oral or gastrointestinal surgery within the preceding 7 days, and known soy hypersensitivity. Participants lost to follow-up in the primary clinical trials were eligible for inclusion in this analysis if intravenous fluid administration status was known. The protocols for both trials recommended ORT supported by ondansetron as needed but did not specify criteria for intravenous rehydration or hospitalization.

### Outcomes and Measurements

The primary outcomes, evaluated at the index visit, were (1) intravenous rehydration, defined as any crystalloid administered through a peripheral intravenous line for the purposes of rehydration; and (2) hospitalization, defined as admission to an inpatient unit outside the ED. We also collected demographic characteristics, frequency of vomiting and diarrhea in the 24 hours pre-ED visit, previous health care visit for the same illness, duration of illness, fever during the current illness (temperature ≥38.0 °C at home or in the ED, adjusted to rectal temperature by adding 1.1 °C to the axillary or 0.6 °C to the oral temperature^25^) or tactile temperature (PECARN), oral ondansetron, trial of ORT, intravenous fluid administration, and ED disposition. Children who received oral ondansetron within 30 minutes of ordering intravenous rehydration were not classified as having received oral ondansetron to promote ORT.

A baseline modified Vesikari Scale score was calculated based on symptoms reported at the index ED visit. The modified Vesikari Scale is a global gastroenteritis severity scale validated in our population. Scores range from 0 to 20 and are categorized as mild (0-8), moderate (9-10), or severe (≥11).^22,26^ The number and duration of vomiting and diarrhea episodes were categorized according to the modified Vesikari Scale score cut-points.^22,26^ Dehydration was assessed using the Clinical Dehydration Scale (CDS), a validated 4-item instrument with high interrater reliability (κ = 0.77).^7,27^ The CDS includes an assessment of general appearance, eyes (eg, sunken), mucous membranes (eg, dry), and tears. Total scores range from 0 to 8 and are grouped to correspond to dehydration severity: none (0), mild to moderate (1-4), and severe (5-8).^27^ The CDS scores were assigned by a trained research assistant at recruitment.

### Laboratory Testing

Stool specimens were requested from all participants for enteropathogen identification. Testing was performed using the Luminex xTAG Gastrointestinal Pathogen Panel (Luminex Corp)^28^ that tests for viruses (adenovirus, norovirus, rotavirus), bacteria (*Clostridioides difficile* [formerly *Clostridium*] toxins A and B, enterotoxigenic *Escherichia coli* heat-labile toxin and heat-stable toxin, *E coli* O157, *Salmonella*, Shiga toxin-producing *E coli, Shigella*, *Vibrio cholera,* and *Yersinia enterocolitica*) and parasites (*Cryptosporidium, Entamoeba histolytica,* and *Giardia*). Specimens collected in the PERC study were also tested for adenovirus, astrovirus, norovirus, rotavirus, and sapovirus.^29^ Results were unavailable at the time of disposition.

### Statistical Analysis

Demographic characteristics were combined from both trials and summarized with counts and percentages for categorical data and medians and interquartile ranges for continuous data. Bivariate analyses adjusted for site and multivariable analyses were used to explore the associations between outcome variables (ie, intravenous rehydration and hospitalization) and the following prespecified, biologically plausible covariates: sex, age in months using a priori determined groups (3 to <12 months, 12 to <24 months, 24 to <36 months, and 36 to <48 months), prior health care practitioner visit during the current illness, country, distance to hospital, infectious agent, duration of vomiting and diarrhea at the time of the index visit, frequency of vomiting and diarrheal episodes within 24 hours of the index visit, fever, ED CDS score, and oral ondansetron administration. Unadjusted bivariate and adjusted multivariable odds ratios (ORs) and 95% CIs were obtained from generalized mixed-effects logistic regression models that employed random intercepts and assumed a simple diagonal covariance structure to adjust for clinical center.

We fit additional multivariable models in order to estimate the adjusted association of infectious agents among the subset of participants from whom stool specimens were obtained. Infectious agents were categorized as negative vs isolated virus vs isolated bacteria vs virus and bacteria codetection vs parasites. In models assessing factors associated with hospitalization, the parasite category was excluded owing to an insufficient number of hospitalized participants. Children 24 months or younger in whom *C difficile* was detected were classified as negative given the high colonization rate and low likelihood of symptomatic causality. Finally, we fit additional multivariable models to explore the separate associations of prior health care visits with and without intravenous rehydration.

Data were analyzed using SAS/STAT software, version 9.4 (SAS Institute Inc). A type I error rate of .05 was used to reject the null hypothesis of no association. All *P* values were 2-tailed. Data analyses were conducted from November 2, 2018, to March 16, 2021.

## Results

### Participants

**Figure 1.**
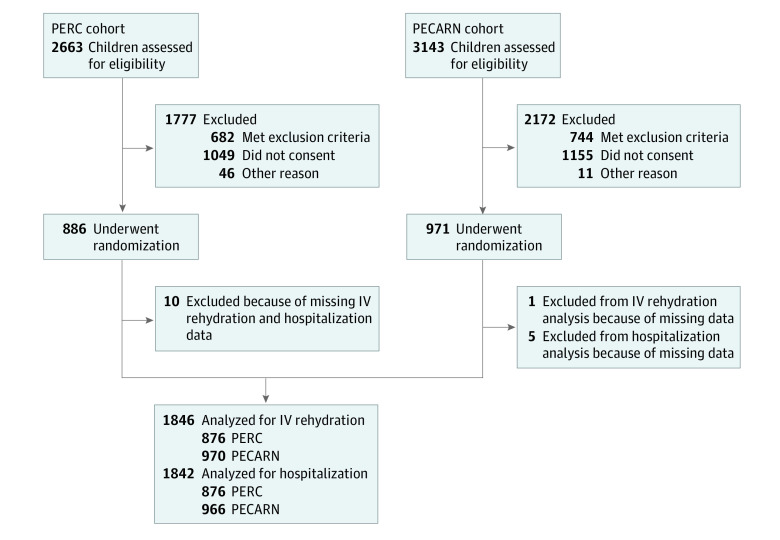
Flow Diagram of Patients Analyzed from PERC and PECARN Cohorts IV indicates intravenous; PECARN, Pediatric Emergency Care Applied Research Network; and PERC, Pediatric Emergency Research Canada.

Of the 1857 participants randomized in the parent trials, we analyzed the results from 1846 children (mean [SD] age, 19.1 [11.4] months; 1007 boys [54.6%]) (Figure 1). Table 1 and Table 2 summarize demographic features. A total of 240 of 1846 participants (13.0%) received intravenous rehydration at the index ED visit, and 67 of 1846 (3.6%) were hospitalized. Oral ondansetron to promote ORT was administered to 534 of 1846 participants (28.9%): 166 of 876 (18.9%) in Canada and 368 of 970 (37.9%) in the US. When 3 or more vomiting episodes were reported, oral ondansetron was administered to 408 of 892 participants (45.7%): 141 of 428 (32.9%) in Canada and 267 of 464 (57.5%) in the US.

**Table 1.** Bivariate Analysis of Variables Associated With Emergency Department Intravenous Fluid Administration

Variable	IV fluids during index ED visit, No./total No. (%)		Model results	
	No (n = 1606)	Yes (n = 240)	Unadjusted OR (95% CI)^a^	*P* value^b^
Sex				
Boys	878/1007 (87.2)	129/1007 (12.8)	1 [Reference]	.82
Girls	727/838 (86.8)	111/838 (13.2)	1.03 (0.78-1.37)	
Age group, mo				
3.0 to <12.0	548/612 (89.5)	64/612 (10.5)	1 [Reference]	.06
12.0 to <24.0	583/679 (85.9)	96/679 (14.1)	1.46 (1.03-2.06)	
24.0 to <36.0	294/337 (87.2)	43/337 (12.8)	1.19 (0.78-1.81)	
36.0 to <48.0	180/217 (82.9)	37/217 (17.1)	1.74 (1.11-2.72)	
ED location				
Canada	803/876 (91.7)	73/876 (8.3)	1 [Reference]	.003
US	803/970 (82.8)	167/970 (17.2)	2.63 (1.38-5.00)	
Distance between home and ED, median (IQR), km^b^	8.8 (4.6-14.9)	9.9 (4.3-21.1)	1.11 (1.02-1.19)	.01
Infectious agent^c^				
Negative	607/661 (91.8)	54/661 (8.2)	1 [Reference]	<.001
Isolated organism				
Bacteria	91/99 (91.9)	8/99 (8.1)	0.92 (0.42-2.03)	
Virus	666/810 (82.2)	144/810 (17.8)	2.81 (1.98-3.99)	
Virus/bacteria codetection	26/30 (86.7)	4/30 (13.3)	2.42 (0.78-7.50)	
Parasites^d^	15/19 (78.9)	4/19 (21.1)	3.89 (1.18-12.81)	
Clinical Dehydration Scale score				
None (0)	1062/1114 (95.3)	52/1114 (4.7)	1 [Reference]	<.001
Mild to moderate (1-4)	524/681 (76.9)	157/681 (23.1)	10.00 (6.92-14.45)	
Severe (5-8)	13/40 (32.5)	27/40 (67.5)	69.54 (31.21-154.94)	
Oral ondansetron administered in the ED^e^				
No	1119/1312 (85.3)	193/1312 (14.7)	1 [Reference]	<.001
Yes	487/534 (91.2)	47/534 (8.8)	0.43 (0.31-0.61)	
No. of diarrhea episodes in 24 h preceding index ED visit, median (IQR)^b^	5.0 (4.0-8.0)	7.0 (4.0-10.0)	1.41 (1.24-1.61)	<.001
Duration of diarrhea, h				
1 to <96	1445/1648 (87.7)	203/1648 (12.3)	1 [Reference]	.30
96 to <120	76/96 (79.2)	20/96 (20.8)	1.49 (0.87-2.55)	
≥120	68/83 (81.9)	15/83 (18.1)	1.24 (0.68-2.25)	
No. of vomiting episodes in 24 h preceding index ED visit, median (IQR)^b^	2.0 (0.0-5.0)	4.0 (2.0-8.0)	1.65 (1.44-1.89)	<.001
Duration of vomiting, h				
No vomiting	417/437 (95.4)	20/437 (4.6)	1 [Reference]	<.001
1 to <24	351/405 (86.7)	54/405 (13.3)	2.94 (1.71-5.04)	
24 to <48	311/367 (84.7)	56/367 (15.3)	3.61 (2.10-6.22)	
≥48	403/504 (80.0)	101/504 (20.0)	4.79 (2.89-7.96)	
Preceding health care practitioner visit				
No	1393/1556 (89.5)	163/1556 (10.5)	1 [Reference]	<.001
Yes, but no IV rehydration	197/261 (75.5)	64/261 (24.5)	2.43 (1.73-3.42)	
Yes, with IV rehydration	5/16 (31.3)	11/16 (68.8)	15.27 (5.14-45.39)	
Fever^f^				
No	783/861 (90.9)	78/861 (9.1)	1 [Reference]	<.001
Yes	820/981 (83.6)	161/981 (16.4)	1.86 (1.39-2.50)	

Abbreviations: ED, emergency department; IQR, interquartile range; IV, intravenous; OR, odds ratio; PECARN, Pediatric Emergency Care Applied Research Network; PERC, Pediatric Emergency Research Canada.

^a^ ORs and CIs compare the odds of receiving IV fluids for each group vs the reference category or for an increase of 5 vomit or diarrhea episodes, or for a 10-km increase in geodetic distance between a patient’s residence and the hospital zip code. A random effect of enrolling clinical site is included in all models. Models are otherwise unadjusted.

^b^ Seven participants did not report a zip code; 6 did not report a number of vomiting or diarrhea episodes in 24 hours preceding the index ED visit.

^c^ Includes participants in whom stool testing was performed and results were available.

^d^ Parasites alone were detected in 12 participants; parasite and virus or parasite and bacterium were codetected in 7 participants.

^e^ Excluding oral ondansetron given within 30 minutes of or after ordering IV rehydration.

^f^ Fever was defined as rectal temperature ≥38.0 °C in the PERC cohort; tactile fever or temperature ≥38.0 °C adjusted to rectal temperature in the PECARN cohort during the current illness, either at home or in the ED.

**Table 2.** Bivariate Analysis of Index Emergency Department Variables Associated With Hospitalization

Variable	Admission to hospital, No./total No. (%)		Model results	
	No (n = 1775)	Yes (n = 67)	Unadjusted OR (95% CI)^a^	*P* value^b^
Sex				
Boys	971/1005 (96.6)	34/1005 (3.4)	1 [Reference]	.52
Girls	804/837 (96.1)	33/837 (3.9)	1.17 (0.72-1.92)	
Age group, mo				
3.0 to <12.0	583/611 (95.4)	28/611 (4.6)	1 [Reference]	.48
12.0 to <24.0	655/678 (96.6)	23/678 (3.4)	0.79 (0.45-1.39)	
24.0 to <36.0	326/337 (96.7)	11/337 (3.3)	0.70 (0.34-1.44)	
36.0 to <48.0	211/216 (97.7)	5/216 (2.3)	0.50 (0.19-1.32)	
ED location				
Canada	854/876 (97.5)	22/876 (2.5)	1 [Reference]	.15
US	921/966 (95.3)	45/966 (4.7)	1.81 (0.80-4.12)	
Distance from home to ED, median (IQR), km^b^	8.8 (4.5-15.2)	11.7 (5.1-31.0)	1.23 (1.12-1.36)	<.001
Infectious agent^c^				
Negative	646/661 (97.7)	15/661 (2.3)	1 [Reference]	.004
Isolated organism				
Bacteria	97/99 (98.0)	2/99 (2.0)	0.88 (0.19-3.93)	
Virus	765/810 (94.4)	45/810 (5.6)	2.89 (1.58-5.31)	
Virus/bacteria codetection	29/30 (96.7)	1/30 (3.3)	1.89 (0.23-15.30)	
Parasites^d^	19/19 (100.0)	0/19	NA	
Clinical Dehydration Scale score				
None (0)	1105/1114 (99.2)	9/1114 (0.8)	1 [Reference]	<.001
Mild to moderate (1-4)	633/681 (93.0)	48/681 (7.0)	11.84 (5.67-24.71)	
Severe (5-8)	30/40 (75.0)	10/40 (25.0)	53.45 (19.10-149.57)	
Oral ondansetron administered in the ED^e^				
No	1253/1308 (95.8)	55/1308 (4.2)	1 [Reference]	.02
Yes	522/534 (97.8)	12/534 (2.2)	0.47 (0.25-0.89)	
No. of diarrhea episodes in 24 h preceding index ED visit, median (IQR)^b^	5.0 (4.0-8.0)	8.0 (4.0-15.0)	1.67 (1.39-2.01)	<.001
Duration of diarrhea, h				
1 to <96	1593/1648 (96.7)	55/1648 (3.3)	1 [Reference]	.15
96 to <120	88/96 (91.7)	8/96 (8.3)	2.21 (0.99-4.94)	
≥120	79/83 (95.2)	4/83 (4.8)	1.28 (0.44-3.70)	
No. of vomiting episodes in 24 h preceding index ED visit, median (IQR)^b^	2.0 (0.0-5.0)	4.0 (1.0-8.0)	1.50 (1.25-1.81)	<.001
Duration of vomiting, h				
No vomiting	430/437 (98.4)	7/437 (1.6)	1 [Reference]	.06
1 to <24	388/405 (95.8)	17/405 (4.2)	2.62 (1.07-6.43)	
24 to <48	354/367 (96.5)	13/367 (3.5)	2.33 (0.91-5.99)	
≥48	478/504 (94.8)	26/504 (5.2)	3.21 (1.37-7.56)	
Preceding health care practitioner visit				
No	1511/1556 (97.1)	45/1556 (2.9)	1 [Reference]	<.001
Yes, but no IV rehydration	243/261 (93.1)	18/261 (6.9)	2.21 (1.24-3.94)	
Yes, with IV rehydration	12/16 (75.0)	4/16 (25.0)	10.26 (3.11-33.86)	
Fever^f^				
No	840/861 (97.6)	21/861 (2.4)	1 [Reference]	.02
Yes	935/981 (95.3)	46/981 (4.7)	1.93 (1.14-3.28)	

Abbreviations: ED, emergency department; IV, intravenous; NA, not available; OR, odds ratio; PECARN, Pediatric Emergency Care Applied Research Network; PERC, Pediatric Emergency Research Canada.

^a^ ORs and CIs compare the odds of being admitted for each group vs the reference category or for an increase of 5 vomit or diarrhea episodes or for a 10-km increase in geodetic distance between a patient’s residence and the hospital zip code. A random effect of enrolling clinical site is included in all models. Models are otherwise unadjusted.

^b^ Five participants did not report a zip code; 2 did not report a number of vomiting or diarrhea episodes in 24 hours preceding the index ED visit.

^c^ Includes participants in whom stool testing was performed and results were available; patients with isolated parasites excluded from estimation of ORs due to low numbers.

^d^ Parasites alone were detected in 12 participants; parasite and virus or parasite and bacteria were codetected in 7 participants.

^e^ Excluding oral ondansetron given within 30 minutes or after ordering IV fluid.

^f^ Fever was defined as rectal temperature ≥38.0 °C in the PERC cohort; tactile fever or temperature ≥38.0 °C adjusted to rectal temperature in the PECARN cohort during the current illness, either at home or in the ED.

### Variables Associated With Intravenous Rehydration

In bivariate analysis adjusted for site (Table 1), variables associated with intravenous rehydration included location of care in the US (167 of 970 [17.2%]); fever (161 of 981 [16.4%]); isolated virus (144 of 810 [17.8%]); parasites (4 of 19 [21.1%]); mild, moderate, or severe dehydration using the CDS score (mild or moderate, 157 of 681 [23.1%]; severe, 27 of 40 [67.5%]); prolonged and more frequent vomiting (≥48 hours, prolonged, 101 of 504 [20.0%]; per 5-episode increase [more frequent] OR, 1.65; 95% CI, 1.44-1.89); more frequent diarrheal episodes (unadjusted OR, 1.41; 95% CI, 1.24-1.61 per 5-episode increase); prior health care visit with intravenous rehydration (11 of 16 [68.8%]); and increased distance between home and the ED (unadjusted OR, 1.11; 95% CI, 1.02-1.19). Oral ondansetron followed by ORT was associated with lower odds of intravenous rehydration (OR, 0.43; 95% CI, 0.31-0.61).

**Figure 2.**
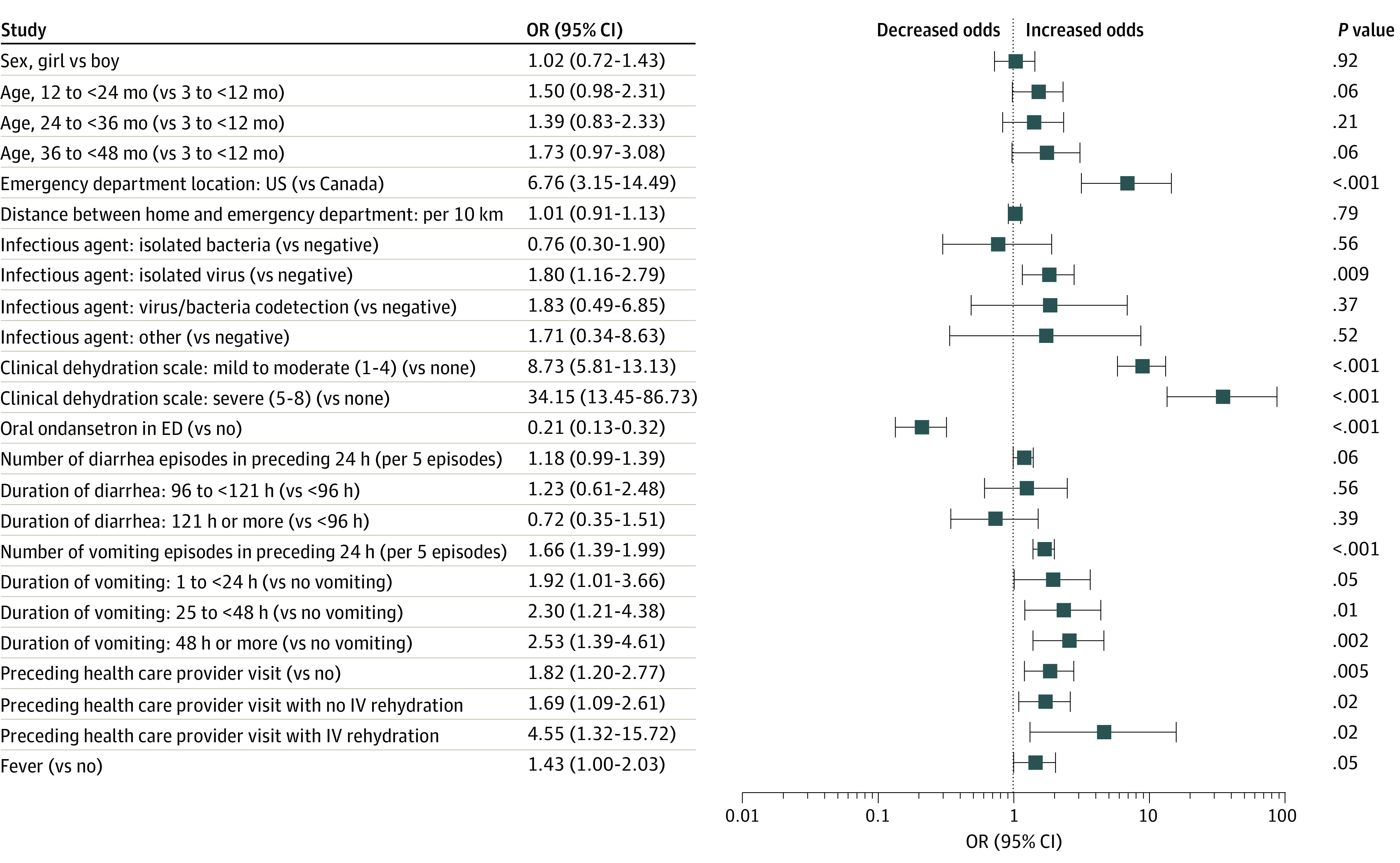
Adjusted Odds Ratios (ORs) From Multivariable Models of Variables Associated With Intravenous (IV) Rehydration Estimates for infectious agent are from a secondary model using 1489 participants for whom stool testing results were available. The “other” category included isolated parasites in the absence of other infectious agents of interest (n = 11) and parasite or virus codetection (n = 7). Estimates for a preceding health care practitioner visit with no IV rehydration and a preceding health care practitioner visit with IV rehydration are from a secondary model using 1690 participants with information about prior health care practitioner visits. All other estimates are from a model using 1692 participants in which the largest variance inflation factor was 1.28 and intraclass correlation within sites was 0.09. ED indicates emergency department.

In the multivariable model, independent variables associated with intravenous rehydration (Figure 2) were CDS scores indicative of mild to moderate (OR, 8.73; 95% CI, 5.81-13.13) and severe (OR, 34.15; 95% CI, 13.45-86.73) dehydration, care in the US (OR, 6.76; 95% CI, 3.15-14.49; relative to Canada), detection of an isolated virus (OR, 1.80; 95% CI, 1.16-2.79; relative to negative), greater number of vomiting episodes (OR, 1.66; 95% CI, 1.39-1.99 per 5-episode increase) and duration of vomiting (OR, 2.53; 95% CI, 1.39-4.61 for ≥48 hours relative to no vomiting), and prior health care visit (OR, 1.82; 95% CI, 1.20-2.77), particularly with intravenous rehydration (OR, 4.55; 95% CI, 1.32-15.72). Oral ondansetron was associated with lower odds of intravenous rehydration (OR, 0.21; 95% CI, 0.13-0.32).

### Variables Associated With Hospitalization

In bivariate analysis adjusted for site (Table 2), the variables associated with hospitalization included increased distance to the ED (US, OR, 1.23; 95% CI, 1.12-1.36), detection of an isolated viral enteropathogen (OR, 2.89; 95% CI, 1.58-5.31), evidence of dehydration on the CDS score (severe, OR, 53.45; 95% CI, 19.10-149.57), prolonged duration of vomiting (≥48 hours, OR, 3.21; 95% CI, 1.37-7.56), greater frequency of diarrheal (OR, 1.67 per 5-episode increase; 95% CI, 1.39-2.01) and vomiting episodes (OR, 1.50 per 5-episode increase; 95% CI, 1.25-1.81), prior health care practitioner visit (yes, with rehydration, OR, 10.26; 95% CI, 3.11-33.86), and presence of a fever (OR, 1.93; 95% CI, 1.14-3.28). Oral ondansetron followed by ORT was associated with lower odds of hospitalization (OR, 0.47; 95% CI, 0.25-0.89).

**Figure 3.**
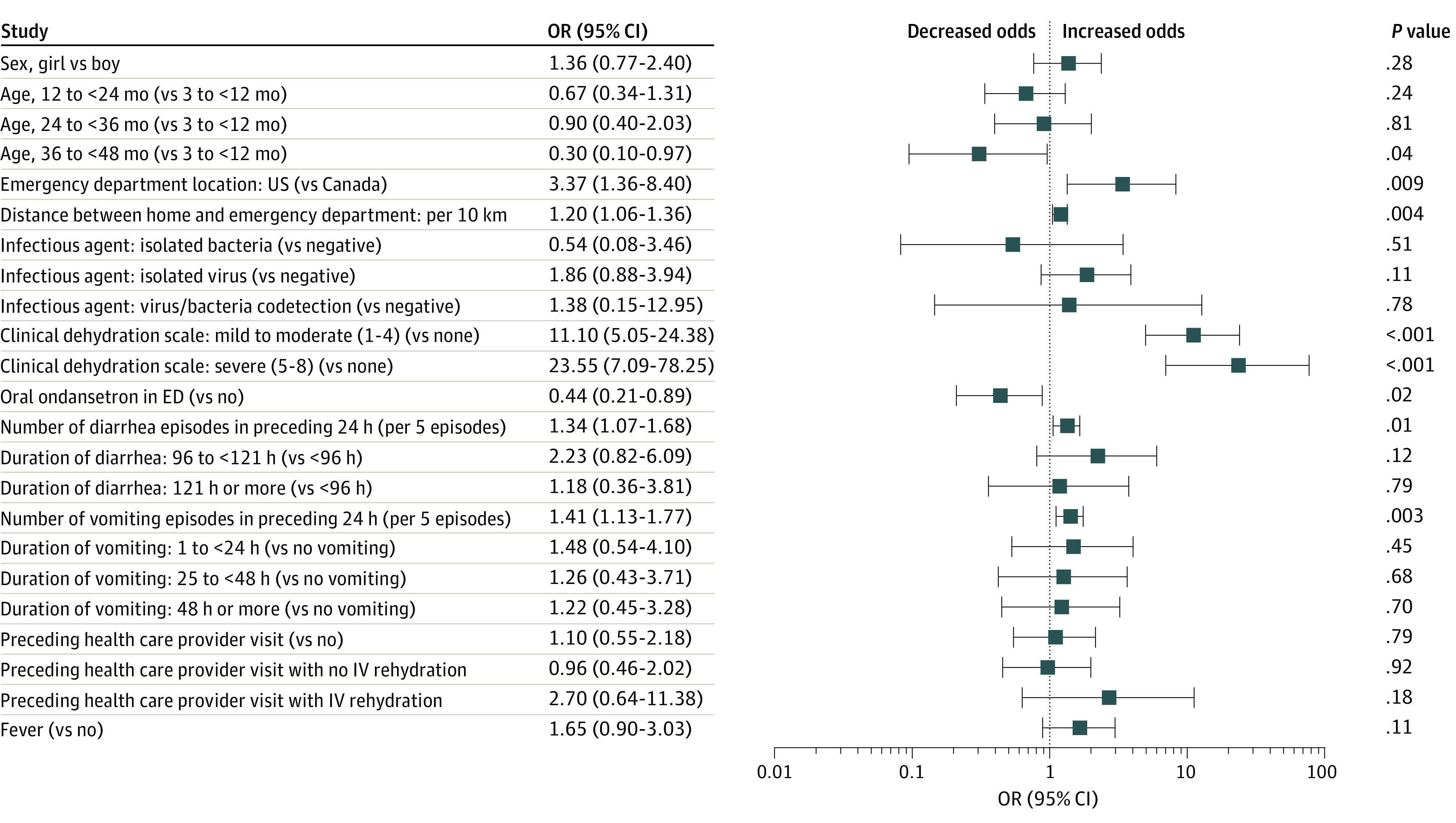
Adjusted Odds Ratios (ORs) From Multivariable Models of Variables Associated With Hospitalization From the Emergency Department (ED) Index Visit Estimates for infectious agent are from a secondary model using 1471 participants for whom stool testing results were available and excluding those with parasites. Estimates for a preceding health care practitioner visit with no intravenous (IV) rehydration and a preceding health care practitioner visit with IV rehydration are from a secondary model using 1690 participants with information about prior visits. All other estimates are from a model using 1692 participants in which the largest variance inflation factor was 1.28. Intraclass correlation within sites was 0.09.

In the multivariable model, independent variables associated with hospitalization (Figure 3) included CDS scores indicative of mild to moderate (OR, 11.10; 95% CI, 5.05-24.38; *P* < .001) and severe (OR, 23.55; 95% CI, 7.09-78.25; *P* < .001) dehydration, care in the US (OR, 3.37; 95% CI, 1.36-8.40 relative to Canada; *P* = .009), and greater number of vomiting (OR, 1.41; 95% CI, 1.13-1.77 per 5-episode increase; *P* = .003) and diarrhea (OR, 1.34; 95% CI, 1.07-1.68 per 5-episode increase; *P* = .01) episodes. Administration of oral ondansetron was associated with a lower odds of hospitalization (OR, 0.44; 95% CI, 0.21-0.89; *P* = .02).

## Discussion

In this planned secondary analysis of the PERC and PECARN trials of oral probiotics in children with AGE-associated diarrhea, we identified independent variables associated with intravenous rehydration and hospitalization. Significant variables included more severe dehydration, care in the US, greater travel distance to the ED, and more vomiting episodes in the 24 hours preceding the ED visit. Oral ondansetron followed by ORT reduced the odds of receiving intravenous rehydration and hospitalization, underlying its importance in the majority of children with AGE.^4,6,30^ These findings can inform quality improvement initiatives to improve outcomes in at-risk children.

After adjustment for clinical characteristics, intravenous rehydration and hospitalization rates were much higher in the US. This finding is most likely explained by a previously characterized difference in willingness to initiate ORT as first-line therapy in children with moderate dehydration between emergency providers in Canada (76% willing) and the US (46% willing).^31^ Differences between Canada and the US, such as perception of medicolegal risks, training, hospital budgets, and parental expectations, have been highlighted as possible explanations to explain greater use of diagnostic imaging in US EDs.^32^ Some of these explanations may apply to intravenous rehydration and hospitalization. For example, the parents of young children in the US, when given the opportunity to make an informed decision, often opt for intravenous over ORT.^9^ Despite country differences, there was no association of site with outcomes. The study protocols standardized the treatment strategies and focused on the promotion of ORT, supported by ondansetron, as required. We did not explore variation in care over time, but as no major changes occurred in the recommended care of children with AGE,^5^ there is no reason to believe there was any variation in care over time.

The proportion of children receiving intravenous rehydration in our study was lower than reported in large retrospective studies (n = 3508 [13%] in Canada^33^ and n = 30 519 [26%] in the US^16^). Although it is encouraging to see lower overall numbers, it should be noted that a leading driver of intravenous rehydration, vomiting,^34^ was absent in 30% of our participants, with 51% having fewer than 3 episodes. This may have explained the low proportion of participants that received ondansetron in our study (28.9%), as ondansetron is indicated for dehydration and frequent and recent vomiting. Importantly, among participants who received intravenous rehydration, 89% were not severely dehydrated, suggesting that intravenous rehydration may be overused in pediatric EDs.

Our results are consistent with evidence that frequent vomiting is associated with intravenous rehydration.^34^ Although gastroenteritis severity is often characterized by the frequency and duration of diarrhea, our multivariable models showed that dehydration severity, quantified using the CDS, was more strongly associated with both intravenous rehydration and hospitalization. Although this association makes intuitive sense, clinical and laboratory assessments of dehydration in children are inaccurate.^7^ Cognitive bias may partly explain the strong association between higher CDS scores and intravenous rehydration. Sunken eyes and dry mucous membranes are both components of the CDS and have long been held as useful clinical signs of dehydration.^35^ Clinical identification of these factors may have driven intravenous rehydration, because subjective clinical measures often overestimate the degree of dehydration,^36^ leading to potentially unnecessary intravenous rehydration. Thus, in high-income countries, otherwise healthy children, even those with high CDS scores, in the absence of circulatory compromise should initially undergo a trial of ORT.

One approach to reduce the use of intravenous rehydration is oral ondansetron followed by a trial of ORT.^37^ Although clinical trials have consistently demonstrated benefit, database studies, which lack detailed clinical characteristics and timelines, have reported less positive results.^38^ Our study, which included timelines related to ondansetron administration, route, and timing of orders for intravenous rehydration, enabled us to ensure that oral ondansetron was administered a minimum of 30 minutes before the order for intravenous rehydration, thereby ensuring it was given and followed by ORT. This is an important concept, as one can reach incorrect conclusions when such an approach is not incorporated into analyses, because in some settings, oral ondansetron and intravenous rehydration are ordered simultaneously. Our finding associating oral ondansetron with a reduction in intravenous rehydration and hospitalization suggest that strategies promoting the appropriate use of oral ondansetron in children with AGE and nonsevere dehydration are crucial to accruing its benefits.^39^

Unscheduled revisit rates for children with AGE range from 7% to 18%^15,38,40^ and are associated with absence of a primary care provider, higher serum bicarbonate,^40^ greater frequency of vomiting and diarrhea,^15^ and administration of intravenous rehydration in the ED.^15^ Similarly, we found that prior ED visits, particularly those associated with intravenous rehydration, were associated with intravenous rehydration at the enrollment ED visit. These findings highlight the importance of administering intravenous rehydration based on presenting clinical features rather than previous therapies. This approach is important because caregivers of children who received intravenous rehydration are less likely to comply with ORT recommendations.^41^

Consistent with previous reports,^38,42,43^ the proportion of children with AGE admitted to the hospital was low (3.6%). Our data suggest that hospitalization was associated with more severe dehydration and care in the US. The latter association may reflect previously published differences in health care resource utilization between Canada and the US in children with AGE.^31^ Although we did not quantify volume of oral fluids consumed or fluid losses in the ED, a higher CDS score is independently associated with ORT failure,^44^ which may have influenced the decision for hospitalization. Consistent with previous reports,^45,46^ we found oral ondansetron to be associated with a lower odds of hospitalization, most likely through the reduction in intravenous rehydration. Thus, promoting oral ondansetron and a trial of ORT in the ED for children with nonsevere dehydration may decrease the risk of intravenous rehydration and demonstrate a strategy that caregivers can continue post–ED discharge.

### Limitations

Our study has several limitations. We enrolled children presenting to tertiary care pediatric centers in high-income countries, and only a small proportion were severely dehydrated. Although baseline characteristics, such as socioeconomic status, would have helped generalize our findings, we unfortunately did not collect this information in both studies in a manner that could be integrated into a joint analysis. Therefore, our results may not be applicable to children presenting for care in rural or low-resource settings where geographic, economic, and etiologic factors may influence the need for intravenous rehydration. As this was a secondary analysis, our data may not be generalizable to patients outside the trial’s eligibility criteria. Furthermore, we were unable to ascertain the role of other potential risk factors, including insurance status, provider experience, or the ability of the child and caregiver to perform ORT. The 2 trials were conducted in countries with different populations and health care systems. An important protocol difference that could have affected results was maximal symptom duration prior to enrollment between PERC (72 hours) and PECARN (7 days). For this reason, country and duration of gastrointestinal symptoms at the index visit were included in the models to address these potential limitations. Finally, we did not describe parental expectations, a potentially influential factor in clinical decision-making. Evidence suggests that parental expectations often contradict clinical practice guidelines and reflect a preference for intravenous rehydration.^9,41^

## Conclusions

In this study of children with AGE and minimal dehydration, independent variables associated with intravenous rehydration and hospitalization included greater dehydration, care in the US, greater travel distance to the ED, and more vomiting episodes in the 24 hours preceding the ED visit. Oral ondansetron followed by ORT was associated with a lower odds of both intravenous rehydration and hospitalization. Cost- and time-saving strategies focused on promoting successful integration of ORT and oral ondansetron into ED care for most children with AGE have the potential to reduce intravenous rehydration and hospitalizations rates.

## Notes

Supplement 1. **PERC Protocol**

Supplement 2. **PECARN Protocol**

Supplement 3. **Statistical Analysis Plan**

Supplement 4. **Data Sharing Statement**
