# Error in Author’s Name

**DOI:** 10.1001/jamanetworkopen.2021.16800

**Published:** 2021-06-07

In the Original Investigation titled “Variables Associated With Intravenous Rehydration and Hospitalization in Children With Acute Gastroenteritis: A Secondary Analysis of 2 Randomized Clinical Trials,”^1^ published April 19, 2021, the middle initial “I.” was missing from author Phillip Tarr’s name in the byline. This article has been corrected.^1^
