# Supplementary material for: Variables Associated With Intravenous Rehydration and Hospitalization in Children With Acute Gastroenteritis: A Secondary Analysis of 2 Randomized Clinical Trials
Source: JAMA Netw Open. 2021 Apr 19;4(4):e216433. doi: 10.1001/jamanetworkopen.2021.6433 (PMC8056281; doi:10.1001/jamanetworkopen.2021.6433)
Supplement: Supplement 1. — PERC Protocol [file jamanetwopen-e216433-s001.pdf]

Impact of Emergency Department Probiotic Treatment of Pediatric Gastroenteritis: Randomized Controlled Trial

Sponsor: The University of Calgary

Principal Investigator: Dr. Stephen Freedman
The Alberta Children's Hospital

Co-Investigators:

Dr. David Johnson
Alberta Children's Hospital

Dr. Serge Gouin
CHU Ste-Justine

Dr. Ken Farion
Children's Hospital of Eastern Ontario

Dr. Katrina Hurley
IWK Health Centre

Dr. Suzanne Schuh
The Hospital for Sick Children

Dr. Philip Sherman
The Hospital for Sick Children

Dr. Andrew Willan
The Hospital for Sick Children

Dr. Marc Gorelick
Medical College of Wisconsin

Dr. David Schnadower
Washington University

Dr. Ron Goeree
McMaster University

Dr. Yaron Finkelstein
The Hospital for Sick Children

Dr. Linda Chui
University of Alberta

Dr. Bonita Lee
University of Alberta

Dr. Xiao-Li Pang
University of Alberta

Dr. Marie Louie
University of Calgary

Dr. Alberto Nettel-Aguirre
University of Calgary

Dr. Naveen Poonai
London Children's Hospital

Funding Agency: Canadian Institutes for Health Research (CIHR)

Other Support: Lallemand Health Solutions
Dr. Thomas Tompkins

Protocol Version: 7.0
Protocol Date: November 1, 2017

1.0 THE NEED FOR A TRIAL

1.1 WHAT IS THE PROBLEM TO BE ADDRESSED?

The burden of acute gastroenteritis (AGE) on children and their families continues to be enormous. **It accounts for 1.7 million pediatric emergency department (ED) visits annually in the United States and nearly 240,000 in Canada.**[1] Children often suffer from prolonged[2] and severe illness; amongst hospitalized Canadian children, 19% have clinical sepsis, 7% seizures and 4% require intensive care unit admission.[3] In a study that we conducted at 11 Canadian EDs, 51% of children experienced moderate to severe disease.[4] Parents rate such episodes as being equivalent to a 10 day admission (moderate) and persistent moderate hearing loss (severe).[5] The burden is augmented by the 50% household transmission rate[2,6] and 42% prolonged work absenteeism rate.[7] Apart from supportive care, **health-care providers have little to offer to relieve suffering.**[8]

Probiotics, which are defined as viable microbial preparations that have a beneficial effect on the health of the host,[9] represent a rapidly expanding field. While they are available as over-the-counter products, according to the National Institutes of Health, the Food and Drug Administration has not yet approved a single agent for any health claims.[10] Further, a 2012 meta-analysis concluded that there is limited data to support their indications and no published pediatric gastroenteritis trials reported on side effects.[11] Thus, understanding the benefits and side effects of probiotics is crucial before widespread use can be endorsed. Although probiotic clinical trials have been performed,[12] only one (still unpublished) has been ED based.[13] Most studies to date have been significantly flawed and **guidelines do NOT endorse their use stating that well-controlled human trials are needed.**[14] Consequently, we and others have found that they are rarely used in clinical practice.[4,15-19] Reasons cited include **(1)** questionable clinical meaning to the outcomes evaluated thus far; **(2)** absence of studies in the appropriate patient population, and **(3)** a lack of confidence in the quality of probiotic agents studied.[19]

Our proposed definitive trial is necessary because it addresses the weaknesses and deficiencies in prior studies. We **(1)** focus on the burden of disease and outcomes of relevance to the infected child and his/her caregiver, **(2)** study outpatient children (>95% of those infected), **(3)** employ rigorous methodology and a sample size significantly larger than any prior study,[12] **(4)** will evaluate the side effect profile and conduct subgroup analyses by etiologic agent, and **(5)** will be free of bias (i.e. industry funding).[20,21] These elements have not been previously addressed by any pediatric probiotic clinical trial. We will additionally investigate **several novel domains**: **(1)** the economics of widespread probiotic use and **(2)** the in vivo impact on immunoglobulin secretion.

This study will address (1) the needs of the medical community, which is aware of the widening gap between the number of important pediatric and adult trials[22,23] and (2) the interest of caregivers in "probiotics" - 71% are aware of the term; 31% believe they may be beneficial in children with diarrhea, and > 90% would administer a probiotic if it could make their child better.[24] Furthermore, our pilot study has provided promising preliminary data and has proven the feasibility of our methods. Thus we are poised to conduct a randomized controlled trial (RCT) that will definitively determine if meaningful benefits are derived from probiotic use and will provide critical information regarding their mechanism of action. This information will impact on practice, the burden of disease, and ensure that children receive the best care possible. The results of our proposed RCT will enable guidelines to either clearly endorse or recommend against the routine use of a probiotic agent in children with AGE.

We also hypothesize that the therapeutic benefits of probiotics in children with AGE vary by infecting pathogen (Appendix 1 Pathogen-Specific Effectiveness). We have assembled a team to bridge the gap between the clinical RCT team, molecular diagnostics, and immunologic to quantify the pathogen-specific effects of probiotics. The latter is likely because there are distinct mechanisms (e.g. invasive, inflammatory, non-inflammatory) by which pathogens cause clinical symptoms.[25] Similarly, probiotic effects are exerted through multiple modes-of-action (e.g. direct antimicrobial activity, competitive exclusion, immune response stimulation, inhibition of virulence gene or protein

expression).[26] *The simultaneous evaluation of pathogen-specific effects on clinical, microbiological and immunological levels has not previously been performed.*

The knowledge gained through this multi-faceted approach will inform understanding of the probiotic-host-pathogen interactions that are responsible for improved clinical outcomes in children with AGE. Our study population, outpatient children, is both the main group of patients who suffer from AGE as well as the main consumer of probiotics. Thus, our findings will be relevant and ready for translation into clinical care while simultaneously opening up avenues for future research.

1.2 WHAT ARE THE PRINCIPAL RESEARCH QUESTIONS TO BE ADDRESSED?

Hypotheses: In children aged 3-48 months presenting to an ED with less than 72 hours of AGE like symptoms, compared with placebo, the administration of a probiotic agent:

1. Will result in a significantly lower proportion of children developing moderate to severe disease over the subsequent 2 weeks.
2. Will not be associated with a significantly greater occurrence of minor side effects.
3. Will be associated with a greater increase in secretory IgA (sIgA).
4. Will have varying effects based on the etiologic pathogen, given the diverse underlying pathophysiologic processes induced by the causative agents[25] and the multiple mechanisms of action of probiotics.[26]

Clinical Efficacy:

Primary Question: For previously healthy children, ages 3-48 months, who present to an ED with less than 72 hours of AGE like symptoms, is the proportion who develop *moderate to severe disease* [Modified Vesikari Score (MVS) $\geq$ 9] following ED evaluation, significantly different in those who receive a probiotic agent (Lacidofil) compared to those who receive placebo?

Secondary Questions: In this group of patients, amongst those receiving active treatment versus placebo:

1. Is there a difference in the (a) *duration of diarrhea* or (b) *duration of vomiting*?
2. Is there a difference in the *proportion who require an unscheduled health care provider* visit?
3. Is there a difference in the *effectiveness of treatment based on the infecting pathogen*?

Side Effect Profile:

Question: In this group of patients, is the proportion that experiences a *side effect* (e.g. bloating, fever, abdominal distention, rash) significantly different in those who receive Lacidofil compared to placebo?

Mechanism of Action:

Question: In this group of patients, are fecal sIgA levels 5 days and 4 weeks after the initiation of treatment higher in those who receive Lacidofil compared to those who receive placebo?

Microbiologic – Stool Pathogen-Specific Load:

Question: In this group of patients, is there a difference in the pathogen specific reduction in stool pathogen load in those who receive Lacidofil compared to those who receive placebo?

1.3. WHY IS A TRIAL NEEDED NOW? Definitive data is lacking to guide clinical decision making and most guidelines do not endorse routine probiotic use.[14,27] Hence, probiotics are rarely prescribed by North American physicians.[4,19,28] **However, there are current trends that obligate an urgent assessment**. First, since probiotics are sold as food supplements, manufacturers can encourage their use while their relevance has yet to be established.[29] Manufacturers have embarked on aggressive campaigns making health claims that may not be supported by rigorous research.[30-33] At stake is the world-wide probiotic market which is growing at 13% annually and is valued at $33 billion/year.[34]

Second, North American and European government agencies remain concerned about their value and safety.[35-37] Third, some institutions are now recommending the routine use of probiotics.[38] Fourth, parents of affected children are often providing probiotics.[17] **We are therefore concerned that probiotic consumption is increasing in the absence of solid evidence. This underscores the necessity to conduct this definitive trial without delay. Prior research on the topic suffers from the following important shortcomings:**

1-Outcome measures used to date have limited clinical meaning: Studies have focused on individual symptoms (e.g. stool duration), without consideration of the full picture of the illness[39] (e.g. fever, vomiting, ED visits, hospitalization). A 2010 Cochrane Review concluded that the instruments employed to date are heterogeneous, lack evidence of validity and focus on outcomes that are not important to participants.[40] Thus, the significance of conclusions reached are questioned.[41,42] **We will employ a validated burden of disease score and will focus on outcomes of relevance to children and their caregivers to enable an evidence-based conclusion to be drawn**.[12]

2-Populations studied to date do not apply to the majority of children: Though 95% or more of children are treated as outpatients,[43] only a handful of small studies have focused on outpatients.[41] Inpatient research cannot be extrapolated to outpatients, as hospitalized children are more likely to benefit from probiotics.[12,44,45]

3-Quality of studies to date is inadequate: Most are small, single-centre[46] and have been conducted by pharmaceutical companies.[47] Many negative probiotic studies remain unpublished.[48] Design issues are a concern: in a 2010 Cochrane Review, only 16% of studies adequately reported the 4 key methodological assessment parameters (i.e. allocation sequence generation, concealment, blinding, and loss to follow-up).[12] Of 175 outstanding dietary research articles selected over the past 7 years by the National Institutes of Health, only 2 addressed probiotics and none AGE.[49] **Hence, high quality studies funded by non-vested parties that assess outcomes of interest to children and parents are needed.[47,50]**

4-Inadequate data available from research in the relevant patient population: No studies to date have evaluated the impact of probiotics on children with gastroenteritis treated in primary care. Only a single ED study has been performed: 129 children received a probiotic or placebo agent and the authors found statistically insignificant trends towards a reduction in stool frequency (30% fewer diarrheal stools) and duration (median 14 hours fewer of diarrhea) amongst those administered a probiotic agent.[51] The groups did not differ in terms of return to normal activities, return for medical care or the need for hospitalization. In light of these potentially important trends, the conclusions of systematic reviews, and the burden of disease – **there are 1.7 million ED visits in the United States and 240,000 ED visits annually in Canada for pediatric gastroenteritis – conclusive data regarding the routine outpatient use of probiotics in North American children with AGE are needed.[1]**

5-Knowledge about the in-vivo Mechanism of Action in AGE is lacking: Our understanding of the mechanism of action of probiotics is limited.[52,53] Possible methods of action are **(1)** *Microbiologic* – by improving intestinal mucosal permeability,[54] modifying the microbiota, inhibiting adherence of pathogenic bacteria, and competing for nutrients;[55] **(2)** *Immunologic* – by upregulating gene expression,[56] inhibiting the activation of pro-inflammatory pathways,[57] increasing the concentrations of anti-inflammatory cytokines,[58] and promoting local antigen-specific immunoglobulin A (IgA) responses.[59] **Studies incorporating both clinical outcomes and the measurement of biomarkers potentially related to the clinical effects are desperately needed.[12,60]**

6-Lack of Probiotic Quality Control: As reported in an RCT comparing 5 probiotic products,[61] not all are equally effective. Strain, viability, and dose are important factors.[62] In North America, most have

never been clinically evaluated,[63] some claim to contain organisms that do not exist,[64] others do not match their labeled microbiologic specifications. Our work with Lacidofil has demonstrated that it reduces epithelial injury,[65,66] prevents bacterial binding, invasion and translocation,[66,67] reduces gastric inflammation,[68] attenuates colonic disease and dysfunction,[66,69,70] improves intestinal barrier function,[71] normalizes corticosterone release,[70] and plays an immunomodulatory role.[66] As a mandatory, yet rarely performed research requirement,[12,72] **we have obtained independent analyses to confirm the viable colony forming unit (CFU) count and microbe identity (Appendix 2-Lacidofil).** We have obtained Health Canada approval for our pilot which has guided this proposal's design. **Hence our study will provide evidence about a high quality product available in Canada.**[73]

1.4 RELEVANT SYSTEMATIC REVIEWS AND NEED FOR THIS TRIAL IN LIGHT OF THESE REVIEWS. Meta-analyses[12,44,47,74,75] are encouraging however, they **(1)** question the clinical relevance of the outcomes evaluated,[12,41,47] **(2)** conclude that publication bias is a concern, and **(3)** advocate for a large RCT,[28] funded by an unbiased agency, in an ambulatory pediatric population.[47] A 2010 Cochrane Review reported reductions in the mean duration of diarrhea (25 hours), diarrhea lasting ≥4 days (risk ratio 0.41), and stool frequency on day 2 (mean difference 0.8).[12] Given the limited clinical relevance of these findings, and the significant between-study heterogeneity, the authors of this and other reviews have called for studies that **(1)** evaluate specific regimens in large numbers of participants, **(2)** identify infectious causes,[41] **(3)** present data separately for important subgroups, **(4)** include identification of the probiotic being tested, **(5)** confirm viability and quantity, **(6)** identify mechanisms underlying the beneficial effects, **(7)** conduct cost-effectiveness analyses,[41,76] and **(8)** are definitive multicentre RCTs.[12,47,77] Our proposed study, which builds on our promising pilot work, addresses all the limitations raised by the previous reviews and will provide the missing pieces of information.

1.5 HOW WILL THE RESULTS OF THIS TRIAL BE USED? The generalizability of the proposed trial will be excellent. If probiotics are effective for specific pathogens, we will develop a knowledge translation (KT) plan to ensure integration into care occurs. We will encourage incorporation into clinical pathways and seek endorsement by knowledge user groups (e.g. Canadian Pediatric Society, Canadian Association of Emergency Physicians).[78] Successful dissemination strategies similar to those previously employed will be adopted.[79-84] This study, which has been endorsed by Pediatric Emergency Research Canada (PERC), a 2011 winner of the CIHR-CMAJ Top Achievements in Health Research Awards, will be conducted at 6 member sites. The network has recently been awarded funding by the Networks of Centres of Excellence Knowledge Mobilization program to build a 36 site network termed *TRanslating Emergency Knowledge for Kids.* The network's purpose is to optimize the transfer of knowledge into non-academic institutions..

Dr. Finkelstein, editor of "KiDrug Alert Journal Club", Journal of Clinical Pharmacology and Population Therapeutics, will disseminate our findings to parents and professionals through this open access venue. Integrated methods will be employed to ensure the lessons learned at ProvLab and the Sherman Lab are rapidly disseminated through publications in peer-reviewed journals enabling others to replicate the process. Epidemiologic findings will be disseminated annually to share new knowledge of circulating pathogens. End-of-grant activities, as described above, will be performed focusing on infectious disease, microbiology, laboratory medicine and public health communities given our strong ties to Alberta Health the Public Health Agency of Canada. From a consumer perspective; our efforts would focus on enhancing the accuracy of labeling of the over-the-counter products, based on our results.

1.6 PLEASE DESCRIBE ANY RISKS TO THE SAFETY OF THE PARTICIPANTS INVOLVED IN THE TRIALS. Well over 200 billion doses of probiotics have been consumed[85] and

no serious side effects have been reported in well people.[12] Five pediatric cases of lactobacillus bacteremia have been reported in which the strain was indistinguishable from the strain administered.[86-88] The cases include short gut syndrome (3), complex congenital heart disease (1), and cerebral palsy and sepsis (1). There have been no reports of adverse overdose events.[16] There is no evidence that probiotic use will worsen diarrhea, result in complications from the disease process, or introduce new toxicity. In our pilot, adverse events were only reported in the placebo group. Information on Lacidofil - testing, safety data, and research by Dr. Sherman's lab are available in Appendix 2 - Lacidofil.

2.0 THE PROPOSED TRIAL

2.1 WHAT IS THE PROPOSED TRIAL DESIGN? Randomized, placebo-controlled, double-blind, multicentre (6), Canadian, ED trial. All children aged 3 months to less than 48 months of age who present to a participating ED will be assessed for eligibility. A total of 886 children will be randomized to receive 5 days of a probiotic agent (Lacidofil – 8 x 10^9 CFU/day) or placebo. The study will be conducted employing methodology suggested by the 2010 CONSORT statement.[89,90]

2.2 WHAT ARE THE PLANNED TRIAL INTERVENTIONS?

ED Intervention: The 1st dose will be administered in the ED. The sachet's contents will be sprinkled into 30 mL of a liquid (ideally ORS) which may be cool (0°C-25°C) but without ice crystal formation. Caregivers will receive instructions on study drug administration, completion of study forms, what and how much fluid to drink, criteria for seeing a health care practitioner or returning to the ED (Appendix 4), and standardized AGE discharge instructions from each hospital.

Home Intervention: All patients will take 1 sachet, based on randomization, every 12 hours for 5 days (total of 9 home doses). They will administer the medication at meal time, mixed with 30 mL of an unfrozen beverage with no ice crystal formations (above 0°C) and ingested immediately to optimize viability. Carbonated and highly acidic beverages should be avoided. We will stress the importance of administering all doses dispensed and the need to communicate with the study team on a daily basis until symptoms resolve. One extra dose/day will be provided (i.e. kits will contain 5 extra doses – total of **15** sachets to account for vomiting or wastage). The dose may be repeated once should the child vomit within 15 minutes of medication administration. Vomiting after medication administration rarely occurs > 1 time.[79] Oral fluid therapy will be encouraged according to established guidelines.[14] Children who are hospitalized will continue as per study protocol as we have successfully done previously.[91] Hospitalization at a non-study hospital site is very uncommon – 1/800 (0.1%) children in the PERC multicentre bronchiolitis RCT were admitted at an alternative site.[91] Should this occur, caregivers will have a letter describing the study, the care-plan, and the contact information of the Site Investigator.

Rationale for Treatment Dose: Although multi-strain products, such as Lacidofil, appear to show greater efficacy than single strains,[92] the optimal CFU/kg dose is unknown.[93] Lacidofil data indicates that a dose of 3-6 x 10^9 CFU/day is effective.[94] Our pilot trial, which employed low (4 x 10^9 CFU/day) and high (8 x 10^9 CFU/day) dose arms, found no side effects with either dose. However, a positive association is postulated to exist between the probiotic dose and clinical benefits[47] with most positive studies employing doses ≥ 6 x 10^9 CFU/day.[44] Thus, we will employ a dose of 8 x 10^9 CFU/day. This should enable us to definitively answer our research question and hence influence future usage. The duration of therapy has been selected based on the best available evidence, the recommendations of experts in the field, previous studies, and the typical duration of most episodes of AGE.[95]

Stool Sample Testing: In keeping with usual common clinical practice, stool samples from all enrolled children will be sent for bacterial culture. Bulk specimens will be obtained whenever feasible. As was done in our pilot study, for children who do not provide a stool specimen prior to discharge, rectal swabs

(2 swabs) will be performed. One sample will be collected for bacterial culture according to site specific practices. The second sample will be collected using a flocked tipped sterile swab (FLOQSwabs™ Flocked Swabs, Copan) and will be stored and frozen (-80ºC) in Universal Transport Media (UTM; Copan). This approach allows us to obtain a specimen for molecular pathogen identification prior to discharge (i.e. prior to probiotic administration altering the accuracy of pathogen identification) on all study participants and will only be tested if an ED bulk stool is not obtained. Viral testing will be performed in batches. We will also attempt to collect a bulk stool sample from all RCT participants in the ED prior to discharge. This specimen is the preferred specimen for pathogen identification testing.

Bulk Stool from Home (Pathogen Identification): Patients enrolled at all sites will be asked to provide additional bulk samples at home. Patients may decline, when obtaining informed consent, to collect bulk stool at home. The need to provide bulk stool samples will be stressed as these samples are required to perform pathogen-specific load quantification (i.e. cannot be performed on rectal swabs).
DAY #0: We will collect a bulk stool sample from all study participants who do not provide specimens in the ED prior to discharge.
DAY #5: We will collect a bulk stool sample from all study participants who provided a Day #0 bulk stool sample.
DAY #28: We will collect a Day #28 bulk stool sample from all study participants who consent to provide a Day #0 and #5 bulk stool sample. To collect specimens, caregivers will be provided with instructions (see Appendix 5) along with stool collection containers.

Initial pathogen identification testing will employ the sample (either bulk stool or rectal swab) obtained in the ED to minimize the impact of probiotic administration on test results. The specimen will be tested using the Luminex xTAG GPP. Day #0 bulk stool specimens collected at home will only undergo pathogen identification testing if the ED rectal swab test does not identify a pathogen. This will ensure that negative rectal swab test results do not reflect inadequate sampling (i.e. rectal swab performed but insufficient stool obtained thereby yielding a false negative test). Day #5 and Day #28 specimens will only be tested if the Day #0 specimen identifies a pathogen. The pathogen identification data is required to assign an etiology to all study participants; this information will be employed to determine the pathogen-specific response across all study aims.

Bulk Stool from Home (secretory IgA): In addition to pathogen identification and quantification, bulk specimens provided by participants on Days #0, #5, #28 will be sent to the Hospital for Sick Children (HSC) to the lab of Dr. Philip Sherman for sIgA testing (Appendix 6-sIgA Procedures). Samples will be stored at -80°C and will be sent to the Hospital for Sick Children (HSC) in bulk shipments from the labs of Dr. Linda Chui and Dr. Xiao-Li Pang. Fecal sIgA analysis will be performed by Dr. Sherman's laboratory which is certified to handle human specimens.[96,97]

If a sample is unable to be provided at Enrolment, on Days 5 and 28, the first sample provided after Enrolment, the Day 5, and Day 28 time points respectively will be accepted.

Patients/caregivers will receive a reminder telephone call or email correspondence, based on preferred method of follow up, one day prior to the scheduled sample return date (i.e. on Day 4 and Day 27).

All specimens will be labeled with the date and time of collection and the subject's study identification number. Once a sample is obtained, caregivers will contact a contracted biomedical courier service who will transport the specimens to the enrolment site with shipment costs covered by study funds. Upon receipt at the laboratory, each sample will be frozen and split appropriately for future testing. This procedure is based on work by the Centers for Disease Control, [143] and our colleagues.[144,145]

Sites will batch ship all frozen stool samples to the Alberta Provincial Laboratory (ProvLab) and the lab of Dr. Xiao-li Pang in Edmonton, Alberta on a regular basis to enable interim laboratory analyses to verify collection and processing procedures. Regular shipments will minimize shipping costs and is acceptable given the stability of nucleic acid in frozen stool samples.[146] All the analyses will be conducted blinded to patient allocation.

2.3 WHAT ARE THE PROPOSED PRACTICAL ARRANGEMENTS FOR ALLOCATING PARTICIPANTS TO TRIAL GROUPS? ***Sequence Generation:*** The Women & Children's Health Research Institute (WCHRI), based at the University of Alberta, will provide data management services for this study. Randomize.net (www.randomize.net), an internet based randomization service, will produce a randomization list stratified by study site, using random-number generating software The lists will be sent to the central pharmacy (ACH) who will prepare consecutively numbered study kits according to the randomization schedule. These will be couriered to the clinical sites, using proper shipment containers and temperature monitors, where they will be stored in the Research Support Pharmacies. ***Allocation Concealment:*** Randomize.net uses industry standard security to send data over the internet. Randomization will be blocked using random blocks of 4 and 6 with a 1:1 allocation ratio. Stratifying by clinical site and blocked randomization will ensure that variations (e.g. site specific practice patterns, gastrointestinal pathogens) are comparably distributed across treatment arms. Only the research pharmacy at the coordinating centre and www.randomize.net will retain the randomization code. ***Implementation:*** Potentially eligible patients (i.e. all children with diarrhea who meet age criteria) will be identified by the triage nurses and will be screened by the Clinical Research Assistant or Nurse for eligibility. A log of all screened patients will be maintained. If eligible, the details of the study will be discussed with the caregivers of all eligible children by the Clinical Research Assistant or Nurse who will seek consent. If consent is obtained, enrolled children will consecutively be assigned a patient ID number by the clinical site. The Clinical Research Assistant or Nurse will collect baseline demographic clinical variables and will complete the data collection forms (Appendix 7-Study Subject Timeline) either on paper or directly into the secure online REDCap database via electronic tablet. Elements of clinical dehydration (Gorelick Score)[98] and baseline disease severity scores (Modified Vesikari Score)[7] will be assigned to enable baseline comparisons between treatment arms. The Clinical Research Assistant or Nurse will then log into randomize.net which will randomize the patient (i.e. it will provide a kit number that corresponds to a study drug kit at the clinical site which will be given to the patient). Following randomization the first dose will be administered (Section 2.2).

2.4 WHAT ARE THE METHODS FOR PROTECTING AGAINST SOURCES OF BIAS? Bias will be minimized by strictly adhering to the 2010 CONSORT Statement recommendations including the use of "third-party" assignment (Section 2.3).[89] Moreover, because the active ingredient constitutes < 10% of the sachet, the probiotic and placebo powders will be identical in appearance, taste, texture and smell. Thus, participants, families, healthcare providers, data collectors (Research Assistants/Nurses), outcome adjudicators (Research Assistants/Nurses), and data analysts will be blinded, thereby preventing bias in outcome assessment. An intention-to-treat analysis will be performed to minimize bias associated with poor compliance and non-random loss of participants.[99] Co-interventions (e.g. antiemetic, intravenous rehydration, antibiotic administration) and other sources of confounding will be recorded. Reporting bias will be avoided by registering the trial at clinicaltrials.gov. Additionally our use of a published score as an outcome measure will protect against the introduction of bias in the assessment of treatment effects.[100]

2.5 WHAT ARE THE PLANNED INCLUSION/EXCLUSION CRITERIA? All patients with gastroenteritis presenting to the ED of 6 participating hospitals will be eligible. The diagnosis of gastroenteritis is at the discretion of the emergency department supervising physician and may or may not include vomiting. Alternative terminologies that reflect as similar diagnosis are acceptable provided they meet all other eligibility criteria. Examples include: viral illness, diarrhea, vomiting, upper respiratory infection, post-infectious gastroenteritis, antibiotic associated diarrhea, toddlers diarrhea, viral infection, enteritis, viremia, fever, and bronchiolitis.

Inclusion criteria (Patients must meet all of the following criteria to be eligible)
1. ***Presence of diarrhea:*** defined as ≥ 3 watery stools in a 24-hour period.[101]
2. ***Duration of vomiting or diarrhea < 72 hours:*** Early administration = greater efficacy.[29,102,103]
3. ***Age 3 to < 48 months:*** AGE severity and frequency are greatest amongst young children.[104]

Exclusion criteria (Patients who meet any one of the following criteria will not be eligible)
1. ***Presence of an indwelling vascular access line or structural heart disease*** (bacteremia risk).[105]
2. ***Taking immunosuppressive therapy, or known history of immunodeficiency*** (bacteremia risk).[106]
3. ***Hematochezia in the preceding 72 hours, underlying significant chronic gastrointestinal problem or inflammatory bowel disease:*** Not including constipation, gastroesophageal reflux or chronic pain.
4. ***Family member with an indwelling vascular access line, on immunosuppressive therapy, or with a known immunodeficiency:*** Does not include use of short course oral (<7 days) or inhaled steroids.
5. ***Bilious vomitus:*** May indicate a diagnosis other than AGE is possible.
6. ***Probiotic use (supplement) in the preceding 2 weeks:*** However, consumption of foods containing probiotics will not result in exclusion as they are ubiquitous.
7. ***Previously enrolled in this trial*** (to ensure that the observations on trial patients are independent).
8. ***Daily telephone follow-up will not be possible while symptomatic*** (travel plans or language barrier).
9. ***Allergy to soy*:** Lacidofil, as well as the placebo product have come in contact with soy during the manufacturing process.
10. ***Pre-existing, or known, pancreatic dysfunction or insufficiency***[107]
11. ***Oral or Gastrointestinal surgery within the preceding 7 days:*** theoretical wound infection risk**.**

Concomitant Medications
The concomitant administration of antibiotics will be permitted and will be at the discretion of the child's treating physician. Children taking antibiotics will *not* be excluded as probiotics remain effective when given concomitantly with antibiotics[108] and their survival is not significantly altered. Similar criteria will be applied to the administration of antipyretics, anti-emetics, and any other medications. As per Standard of Care at the participating sites, Oral Rehydration Solution (ORS) will be provided during the emergency department visit to enable the performance of oral rehydration therapy. In keeping with institutional Standard of Care, patient/parent discharge instructions that will be provided, as specified in protocol section 2.2, will encourage the ongoing use of appropriate ORS following discharge.

2.6 WHAT IS THE PROPOSED DURATION OF TREATMENT PERIOD? Five days.

2.7 WHAT IS THE PROPOSED FREQUENCY AND DURATION OF FOLLOW-UP? Daily telephone or e-mail survey follow-up will occur, 7 days/week, **until both the diarrhea and vomiting have resolved.** We will also conduct follow-up on days #5 and #14 even if symptoms have resolved.

2.8 WHAT ARE THE PROPOSED PRIMARY AND SECONDARY OUTCOME MEASURES?
Primary Outcome (Clinical): The primary outcome is the development of moderate-severe disease in the 2 weeks after the index ED visit as measured by the MVS (Appendix 8-MVS).[7] The original 20 point Vesikari Score has been employed as a dichotomous variable in many clinical studies[109-117] despite

limited evidence supporting its use. However, it has been shown to correlate with other meaningful measures such as caregiver anxiety, helplessness, and stress.[118] Recently, increasing severity scores were associated with higher parental worry, greater changes in the child's behavior, and trends towards greater impact on the parents' daily activities and higher parental distress.[119] ***So, why did we develop a Modified Score?:*** Percent dehydration, an element of the original score, is challenging to determine. While using baseline and rehydrated weights is the gold standard,[98] this is often of limited value due to difficulties in ensuring follow-up, determining when rehydration has occurred, and the variation related to timing of voiding, stooling, eating, and drinking. Moreover, clinical estimates of dehydration are extremely inaccurate.[120] Thus, this element is omitted or incorrectly assigned in most studies. The modified score which we have created includes an important and easy to obtain outcome that reflects global disease severity-***need for unscheduled future health care visits within 2 weeks of the index visit***.[7] This is supported by evidence that the utilization of professional medical care correlates with disease severity.[118] **Unscheduled future health care visits** is a powerful marker that has the capacity to alter clinical practice and influence decision makers. Similar modifications have been performed previously when percent dehydration has been unavailable[118,121] and we have previously shown that because ED care does not alter the disease process in AGE, ED revisits are very common (publication attached).[79,122] **The MVS[7] is presented** below (Table 1), with the score structure (0, 1, 2, 3 points) unaltered from the original score.

**Table 1. Modified Vesikari Scale Score**				
Points	0	1	2	3
Diarrhea Duration (d)	0	1-96 hours	97-120 hours	≥ 121 hours
Max # of diarrheal stools/24 hr period	0	1-3	4-5	≥ 6
Vomiting Duration (d)	0	1-24 hours	25-48 hours	≥ 49 hours
Max # of vomiting episodes/24 hr period	0	1	2-4	≥ 5
Max Recorded Fever	< 37.0˚C R	37.1-38.4 ˚C R	38.5-38.9˚C R	≥ 39.0˚C R
Unscheduled Future Health Care Visit	0%	-	Primary Care	Emergency Dept.
Treatment Administered	None	Rehydration	Hospitalization	-

Characteristics of the MVS: We prospectively evaluated the MVS in an 11 centre (455 children) ED study[7] in children meeting eligibility criteria as planned for the current proposal (≥3 stools in a 24 hour period and <72 hours of symptoms) which found that it effectively measures global disease severity. Factor analysis revealed that item correlations were acceptable and supported the appropriateness of retaining all factors. Multi-collinearity was not a problem and the correlations between the MVS and other measures of clinical significance were in the expected direction. Disease severity was associated with prolonged daycare ($P = 0.01$) and work ($P = 0.002$) absenteeism. The MVS had a normal distribution with minimal kurtosis (-0.14; SE: 0.24) and skewing (0.39; SE: 0.12). There was good variation across severity ranges (49% mild; 21% moderate; 30% severe). Variation between institutions was insignificant ($P = 0.11$) and complete follow-up was achieved in 91% of participants.

How will it be Calculated?: Following enrollment (Time 0), follow-up will occur daily until both the diarrhea and vomiting have resolved (Section 2.7). Once follow-up is complete (Day #14) each variable is assigned a score for the entire study period (Time 0 to Day #14); each patient gets a single total score for the study. Variables are scored based on the worst 24 hour period (e.g. maximal number of episodes of vomiting in a 24 hour period) or on the total duration of symptoms (e.g. number of days of vomiting) or are based on the occurrence of an outcome (e.g. hospitalization).

What if at baseline the pre-enrollment MVS is ≥ 9?: Regardless of the score assigned at Time 0 (i.e. *pre-enrollment score*), EVERYONE reverts to a score of 0 at enrollment (i.e. the study evaluates the

impact on the disease process going forward). The *pre-enrollment score*, which is based on symptoms in the 72 hours prior to presentation, will serve as a covariate in a secondary analysis of the primary outcome and will be employed for sub-analysis purposes. An example is provided (Appendix 8-MVS). **The primary outcome** *(the presence of moderate-severe disease, as defined by a MVS of ≥ 9 during the 2 week follow-up period)* **will ONLY include symptoms and outcomes that occur following the ED visit (i.e. after randomization) and will not be directly impacted by the *pre-enrollment score*.** ***Why a cut-point of 9?:*** With the original score, severe disease was defined as ≥ 11;[109,110,115,116,123-125] moderate as ≥ 9.[126] In our derivation study,[7] construct validity was proven by using scores of ≥ 9 to define moderate and ≥ 11 to define severe disease. These cut-points were associated with significant increases in other measures of disease severity [e.g. daycare (P=0.01) and work absenteeism (P=0.002).[7]

<u>**Secondary Outcomes (Clinical):**</u>

1. **The duration of diarrhea:** Time from treatment initiation until the appearance of the last watery stool[127-129] as reported during daily phone conversations.
2. **The duration of vomiting:** Limited data indicate that probiotic administration may reduce vomiting.[102,130] Recovery will be evaluated in children who vomit ≥ 3 times over the 24 hours prior to the ED visit and defined as "time from treatment initiation until last vomiting episode." We have previously reported that vomiting frequency predicts outcomes in AGE.[131]
3. **Return visits for unscheduled care to a health care provider related to vomiting, diarrhea, dehydration, fever, or fluid refusal, within two weeks:** Not included will be scheduled visits (e.g. re-assessment, vaccinations). This outcome is important as > 50% of children have a follow-up office visit,[43] 8-18% require an ED visit,[132] and 5-8% are hospitalized.[43]

Additional Outcomes: Work and daycare absenteeism.

<u>Side Effect Profile</u>***: To determine if short course probiotic administration to young children with AGE is associated with an increase in minor side effects.*** As stated by the NIH, probiotic safety needs to be studied scientifically.[133] Groups will be compared regarding the development of any side effects with particular attention paid to bloating, abdominal distention, duration of fever, and buttock rash. The importance of evaluating side effects has been highlighted by a recent adult pancreatitis study which found an unexpected increase in mortality in probiotic treated patients.[107]

<u>Mechanism of Action</u>***: To determine if probiotic administration increases fecal secretory IgA levels in children with AGE (Appendix 6).*** The first stool sample produced following enrollment will be collected along with samples on days 5 and 28. sIgA is a key element in the gastrointestinal immune defense as it agglutinates microorganisms and prevents pathogen adherence to mucosal surfaces.[134-137] Evaluating sIgA in children with AGE has been identified as a needed element to advance this field of research.[138,139] Animal studies have reported a substantial increase in anaerobic bacteria in the absence of normal sIgA and that normalization of sIgA production results returns intestinal microbiota to its regular composition.[140] Probiotics are believed to enhance host immunity by regulating inflammatory cytokines[141] and by increasing sIgA production.[142,143] In human studies, probiotic administration appears to increase fecal sIgA concentration in healthy adults,[144] children,[145] infants,[146,147] and pre-term infants.[148] However, correlation with clinical outcomes has not yet been evaluated. We will determine if fecal sIgA levels are greater amongst children treated with a probiotic agent compared with placebo. Levels will be correlated with clinical findings. However, **experiments correlating probiotic administration, clinical outcomes, and fecal sIgA levels in the context of enteric infection have not been conducted.** Specifically, we will determine, at a pathogen-specific level, if fecal sIgA levels are higher in children treated with a probiotic agent compared with placebo, and if higher fecal sIgA levels are associated with improved clinical outcomes.

<u>**Pathogen Load Quantification**</u>**:** ***To determine if a 5-day probiotic treatment course administered to children with AGE results in pathogen-specific reductions in stool pathogen load***. Our team, which includes experts in molecular diagnostics, virology and bacteriology, has the capacity to quantify the impact of probiotic administration on stool pathogen infectious loads. These measures represent disease severity in individuals with AGE;[149-153] higher stool loads are associated with more severe symptoms, prolonged shedding,[150,151,153,154] hospitalization,[155] and the presence of virus in the blood (i.e. viremia).[155,156] In children with AGE, stool viral loads correlate (r = 0.80, P<0.001) with the Vesikari Score.[157] Bacterial loads, analyzed from other biological specimens, also have clinical relevance – for example, sputum *Pseudomonas aeroginosa* loads correlate with clinical status[158] and those of *Neisseria meningitides* in serum are associated with death and permanent sequelae.[159] All of this work builds on the model of human immunodeficiency disease, where serum viral load has been a key prognostic marker for decades.[160] Consequently, stool infectious load quantification is increasingly encouraged.[161] Our team, which has led many key advances in molecular virology[162-167], has developed a standardized approach (see Section 2.9.2) to quantify **stool viral and bacterial loads, enabling us to quantify an objective marker of disease severity**.

2.9 HOW WILL THE STUDY AIMS AND OUTCOMES BE ACHIEVED?

<u>2.9.1 Aim #1: Clinical Benefits – Modified Vesikari Scale Score</u>

The Modified Vesikari Scale score will be assigned based on data collected during the follow-up period via electronic survey or phone call. A single score is assigned to each of the 7 elements representing either symptom duration, the maximal frequency of vomiting, maximal frequency of diarrhea, maximal recorded body temperature, and subsequent healthcare use and treatments provided. Each participant will have a single, Modified Vesikari Scale score assigned at the conclusion of the follow-up period which reflects the severity of the child's disease. The relationship between the assigned score, the identified pathogen, and probiotic exposure (active/placebo) will be quantified.

<u>2.9.2 Aim #2: Microbiologic – Stool Pathogen-Specific Load</u>

All children with a Day #0 viral or bacterial pathogen identified *and* who provided bulk stool specimens on Days #0, #5, and Day #28 will have samples tested for pathogen-specific load quantification. Results will be reported as NA copies of pathogen/gm and the difference between Days #0 and SUBSEQUENT TEST DAYS will represent the participant's pathogen-specific load reduction. The relationships between pathogen-specific load reduction, infecting pathogen, and probiotic exposure (active/placebo) will be quantified. In addition, to enhance the clinical interpretation of pathogen load reduction, we will explore the relationship between Modified Vesikari Score and pathogen load reduction separately.

Quantification procedures will be standardized to ensure that the homogeneity and proportion of stool included in each analysis is consistent between samples (intra- and inter-patient) and hence per reporting unit (gm). To achieve this degree of standardization, a 20% (weight/volume) suspension of stool specimen will be prepared with phosphate-buffered saline (PBS) and clarified by centrifugation. Standardization will be facilitated by conducting batch analyses including Days #0 and #5, and Day #28 specimens from each participant in the same run, thereby eliminating inter-run variation.

Quantification of enteric viruses: This will be performed as previously described by our team (Pang, Lee).[166] In brief, samples will be thawed, mixed by vortexing and a 20% stool specimen suspension will be prepared and clarified by centrifugation. Total NA will then be extracted and eluted using the NucliSENS® easyMAG® automated system (bioMerieux, Durham). Viral NA prepared from non-study stool samples testing positive for well-characterized enteric viruses (i.e. rotavirus, norovirus GI/GII, and adenovirus 40/41) will be used as positive controls. The primers and probes for the detection of norovirus, rotavirus, and adenovirus[164,168-171] will be labeled with Fam detector and Tamara quencher dyes (Applied Biosystems). Individual real-time PCR reactions for each virus will be performed. After

incubation for denaturing, PCR amplification will be performed and profiles will be collected and analyzed using Sequence Detection Software version 1.0. To quantify the 3 viruses, an external standard curve will be established using 10-fold dilutions from 1 copy to 1.0 x 10^8 copies.

Quantification of enteric bacteria: Building on our prior work and collaborating with team members (Pang, Lee), we will employ methodology as described above for the viral targets, to quantify bacterial loads. This will be determined for stool samples positive for each bacteria using singleplex real-time PCR assays for each respective bacteria (*Salmonella, Campylobacter, Shigella, E. coli, Yersinia*). Standard curves correlating CFU and crossing point of the real-time PCR assay for each organism will be created by performing real-time PCR on 10-fold dilutions of standardized bacterial suspensions that will also be plated onto sheep blood agar plate to determine the CFU count.

2.9.3 Aim #3: Immune Response – Fecal Secretory Immunoglobin A (sIgA) Quantification

sIgA testing will be performed employing the Eagle Biosciences Secretory IgA ELISA kits (catalog #: SGA35-K01) in accordance with the manufacturers instructions.

2.10 HOW WILL THE OUTCOME MEASURES BE MEASURED AT FOLLOW-UP? All caregivers will receive discharge instructions that will include information on tasks required following discharge. Training materials have been developed based on the 3 site probiotic pilot study.

1. *Daily Telephone/Survey Communication:* At the index visit, caregivers will be asked their preferred method of communication – electronic (i.e. email survey) versus telephone. Surveys (telephone and email) will be offered in French and English for sites requiring bilingual data collection. Following discharge, site Clinical Research Assistants or Nurses will contact the family daily until both the diarrhea and vomiting have resolved employing the identified method. A standardized script or survey/data collection form will be employed. If phone is opted for, the caller will enquire about ongoing symptoms, medical evaluations, treatments, child care and work absenteeism, and side effects. Detailed questioning will follow positive responses. The survey will employ advanced logic to enhance ease of use. If the caregiver does not complete the survey within 48 hours, a telephone follow-up will be performed. ***Compliance*** will be assessed on day #5 and final data points will be collected on day #14. Protocols will be developed to deal with caregiver questions in accordance with institutional requirements. To maximize validity, caregivers will be reminded of the importance and method of administering the probiotic/placebo. Similar schemes have been successfully implemented by the principal investigator,[79,122] other PERC multicentre studies,[82,91] and was employed in the pilot. Caregiver report (telephone/survey) will serve as the primary source document.

2. *Chart Review:* We will verify data regarding revisits, intravenous hydration, hospitalization, and microbiology testing using each centre's medical record database.

3. *Database Reviews:* Provincial databases (e.g. National Ambulatory Care Reporting System; Alberta Ambulatory Care Classification System; Alberta Health Care Insurance Plan) and Canadian Institute for Health Information databases will be employed to verify future health care provider use.

2.11 WILL HEALTH SERVICE RESEARCH ISSUES BE ADDRESSED? As called for by the 2010 Cochrane review,[12] an economic evaluation will be conducted by Dr. Willan and Mr. Goeree[172-177]alongside the clinical trial (Appendix 9-Economic Analysis Plan). We will monitor work absenteeism, as this is the major item contributing to cost.[122] Moreover, days of diarrhea has been found to correlate with work absenteeism,[178] and a recent pediatric, Canadian ED study found that > 50% of the societal costs occur in the 15 days following the ED visit.[179] Hence, if effective, cost savings are likely from a societal perspective due to the inexpensive nature of probiotics and the economic benefit derived from

reduced work absenteeism. Because adding a therapeutic intervention may add to overall health care costs, willingness to pay will be determined. The incremental cost effectiveness will be determined by assessing resources and costs associated with the treatment of AGE for children who receive the current standard of care compared to those who receive a probiotic.

2.12 WHAT IS THE PROPOSED SAMPLE SIZE AND WHAT IS THE JUSTIFICATION FOR THE ASSUMPTIONS UNDERLYING THE POWER CALCULATIONS (APPENDIX 10)?

Clinical Outcome: The sample size is based on the assessment of the between-group difference in proportions of children with a *post-randomization* score ≥ 9 on the MVS. **This is a superiority study** in which the adoption of probiotic use can be recommended if the rate of the primary outcome is significantly lower amongst those who receive the probiotic medication. Calculations are based on a **two-sided type I error (α) of 0.05 and power (1-β) of 0.90**. The null hypothesis is H_0: $P_c - P_I = 0$, where P_I and P_C are the event rates in the intervention and control groups respectively. The alternative hypothesis is H_A: $|P_I - P_C| > 0.10$ (*i.e.* the event rates will differ by at least 10 percentage points).
Minimal Clinically Important Difference (MCID): Ten content experts from the US and Canada were surveyed regarding the MCID. Absolute risk differences ranging from 7.5-15% were suggested. We chose a conservative estimate of 10% for the primary outcome (number needed to treat of 10).
Outcome in Control Group: Our estimate for the development of moderate to severe AGE in the controls is based on data collected as part of our 2009 evaluation of the MVS in 455 children aged 3 – 48 months, with < 72 hours of symptoms, who presented to one of 11 Canadian EDs (Section 2.8).[7] Using the ED visit as time 0, 25% of eligible children had scores consistent with moderate to severe disease following discharge. This is lower than previous reports of ED[110,125] and community populations[109,124,126] because we did not include symptoms that existed prior to the visit. However, Dr. Schnadower's group in the United States has just completed data collection on 282 children enrolled at 6 sites in the United States and they found that 24% of children in their sample had scores consistent with moderate to severe disease following discharge (personnel communication September 6, 2012). Since our study population and method of MVS calculation in the derivation and recent validation studies and the current proposal are the same, 25% is a very accurate estimate. Given the above, the required sample size to compare proportions between two different groups is 670.[180]
Sample Size Adjustment Calculation: Based on previous work by our group with similar follow-up designs[79,91,181] and extensive reviewer feedback, we have assumed a 10% loss to follow-up (670/0.9=744), 5% drop out ($744/(0.95)^2$=825), and 2.5% drop in (caregivers who decide to buy a probiotic agent at a pharmacy to administer to their child) rate ($825/(0.975)^2$=868). Adjustment for O'Brien-Fleming monitoring boundaries requires a further 2% increase. Thus, **the total number randomized (final sample size) will be 886.**

Side Effect Profile: To date, clinical trials employing probiotics have not attributed any adverse events to probiotic administration.[12] We suspect that minor side effects have not been documented; however, clinicians need to have an understanding of the side effect profile in order to enable caregivers to make an informed treatment decision. Given our sample size, a significant difference between groups will be easily detected (i.e. 80% power to detect an increase in reported adverse events from 5% to 10%).

Mechanism of Action: A study evaluating the impact of formula supplementation with oligosaccharides found fecal sIgA values of 729 and 377 μg/g in the intervention and control groups respectively.[182] If we assume a clinically significant difference of 300 μg/g, a standard deviation of 500 μg/g, 80% power and a type I error of 0.05, the required sample size is 45 subjects/group. Thus we will aim to include a minimum of 100 patients which will be recruited from all study sites, with the exception of the IWK Health Centre.

<u>**Pathogen-Specific-Effectiveness Study**</u> (Table 2): Home Stool Collection on Days #0 and #5 will be completed at all 6 study sites. It is anticipated that bulk stool will be collected on 25% of children in the ED and 75% of those requiring home Day #0 collection. Of those providing an ED/home Day #0 specimen, 75% will provide a Day #5 sample.[183,184] We will collect specimens to enable pathogen identification on all study subjects (n=886). These will be paired with Modified Vesikari Scale score data from the estimated 797 children (90%) who will complete follow-up. Data from these 797 children will support the conduct of Aim #1 analyses. Assuming ~50% viral (n=399), ~40% unidentified (n=318), and ~10% bacterial (n=80), and trusting randomization (~50% probiotics, ~50% placebo) we anticipate a minimum of 40 children per arm in our smallest group. Day #0 and 5 paired samples will be obtained from ~465 children of which ~232 will be positive for a virus and ~46 for a bacteria. Thus, pathogen load reduction calculations will be performed for 278 participants. These accrual estimates are summarized in a diagram in **Appendix 12**.

Table 2. Current and Anticipated Enrollment and Specimens per Study Aim

	**Required**	**Bridge Funding**		**Pathogen-Specific-Effectiveness Study**			**Total**
		**Collected**	**To be Collected**	**10/2014 – 09/2015**	**10/2015 – 09/2016**	**10/2016 – 09/2017**	
Aim #1	• Modified Vesikari Scale score<br>• ED stool sample or rectal swab	77 (actual)	120	200	200	200	797
Aim #2	• Days #0 and #5 stool samples<br>• Positive pathogen identification	14	43	74	74	73	278
Aim #3	• Days #0 and 5 stool samples	24 (actual)	72	123	123	123	465

2.13 WHAT IS THE PLANNED RECRUITMENT RATE (APPENDIX 11)?

The 5 original proposed study sites saw 10,344 children aged 3 – 48 months with AGE in 2011 (a 17% increase since 2009). During our pilot RCT, 2.1% of children with AGE aged 0-4 years were enrolled. Based on the published literature and our data: **(i)** presenting November 1 – May 31 between 8:00 – 24:00 (55%), **(ii)** meet definition of diarrhea (50%), **(iii)** < 72 hours of symptoms (45%), **(iv)** absence of exclusion criteria (80%), and **(v)** provide consent (50%), our best estimate is that 4.7% of children with AGE aged 3 - 48 months will be enrolled. The difference between our pilot and the best point estimate is due to the requirement of daycare attendance in our pilot study. Based on our experience with AGE,[79,185] and multicentre trials,[82,186] we believe that we should employ our worst case scenario recruitment estimate (3.1%) which will enable us to enroll our full sample size over three AGE seasons. The only prior North American ED study, which employed similar eligibility criteria, recruited 129 subjects at 1 site in just 8 months[51] therefore we believe our recruitment plan is realistic. The data outlined in Appendix 11 is for the initial 5 sites. A sixth study site has been added to improve enrolment and projected timelines. Data related to gastroenteritis visits is unavailable for the sixth study site.

2.14 ARE THERE LIKELY TO BE ANY PROBLEMS WITH COMPLIANCE?

While infrequently reported and not considered to be problematic,[187] non-compliance is unlikely related to probiotic side effects.[12] Participant withdrawal has primarily been related to the primary illness.[12] A recent study reported 108% compliance due to medication re-administration in subjects who vomited.[188] As the intervention is of a short duration, the burden to caregivers is minimal. In our pilot, compliance was 91% as reported by caregivers and verified by return sachet counts. This does not reflect the impact of vomiting following medication administration. A recent ED probiotic study reported that 87% of caregivers found the probiotic and placebo powders to be "very" or "somewhat" easy to administer.[51] Hence, we do not anticipate compliance problems; nonetheless, we will track compliance by obtaining unused sachet counts (day #5) and requesting their return (day #14).

2.15 WHAT IS THE LIKELY RATE OF LOSS TO FOLLOW-UP?

Our previous ED pediatric AGE research achieved telephone follow-up rates of 98-99% on Day #3 and 96-99% on day #7.[79,122] Similar success has been documented in prior PERC (99%) [82,91] multicentre studies. We will err on the conservative side and estimate a 10% loss to follow-up. If daily contact does not occur we will collect data from missed days on subsequent days when caregivers are contacted. The use of databases (Section 2.10) will supplement the daily telephone calls.

2.16 HOW MANY CENTRES WILL BE INVOLVED?

Six EDs that are members of PERC, a network which has extensive experience conducting large scale clinical studies,[4,82,91,147,189-191] will participate – Alberta Children's Hospital (Calgary), Hospital for Sick Children (Toronto), Children's Hospital of Eastern Ontario (Ottawa), Centre Hospitalier Sainte-Justine (Montreal), IWK Health Centre (Halifax), and the London Children's Hospital (London).

2.17 WHAT IS THE PROPOSED TYPE OF ANALYSES?

All analyses will be undertaken by the intention to treat principle. Adverse events will use the "as treated" principle. Patients who drop out or crossover will be followed and included. All statistical tests of hypotheses will be two-sided. Baseline characteristics will be compared between groups using frequency counts and percentages for discrete variables, and means, medians, standard deviations, and interquartile ranges for continuous variables. Baseline characteristics will be analyzed to determine if there is a need to adjust for differences between groups. Sensitivity analyses will be performed to assess the possibility and consequences of losses to follow-up not occurring at random, as well as to assess the classification of children who have multiple pathogens identified (<5%). Initial classification will be based on Day #0 load (i.e. classified based on higher load); re-classification will evaluate the impact of classification according to the agent with the lower pathogen load.

<u>**Clinical-Primary Outcome:**</u> The proportion of children with moderate to severe disease (i.e. MVS ≥ 9) will be analyzed by comparing proportions utilizing a Mantel-Haenszel test, stratified by clinical centre. **Significance for the primary outcome measure will be determined using a two-sided 0.05 level.** The *pre-enrollment* MVS will not be included in the primary analysis as we do not anticipate the baseline and post-intervention scores to be correlated. Secondary analyses of the primary outcome will employ logistic regression methods to adjust for covariates that may be imbalanced between groups (e.g. age, *pre-enrollment* MVS, severity of baseline diarrhea and vomiting, hydration assessment, need for hospitalization at index visit). We will also analyze the MVS as a continuous variable through a stratified Wilcoxon rank-sum test. The mean benefit will be explored, separately, in relation to:

1. Pathogen-group: virus vs. bacteria vs. not identified
2. Viral agent: rotavirus vs. norovirus vs. adenovirus
3. Bacterial agent: *Campylobacter* vs. *Salmonella* (only ones anticipated to have sufficient numbers)

All analyses will first employ 2-way ANOVA to assess main effects and interactions of treatment assignment and pathogen group. To assess for other covariates and potential confounders, multivariable

regression models including treatment, pathogen and other key covariates (e.g. age, sex, Modified Vesikari Scale score at enrollment, hospitalization, antibiotic use) will be constructed.

Clinical-Secondary & Tertiary Outcomes: The overall significance level for statistical tests on the **secondary outcomes** will be set at 0.05. Holm's method will be used to adjust for multiple comparisons. The continuous variables of **(1)** duration of diarrhea and **(2)** vomiting will be measured in hours and analyzed with a Van Elteren test, stratified by clinical centre. **(3)** Unscheduled health care visits will be analyzed using a Mantel-Haenszel test, stratified by clinical centre. The **tertiary outcomes** of **(4)** number of days the child is absent from daycare and the **(5)** caregiver is absent from work will be analyzed using an appropriate model with robust estimates for standard errors. Dichotomous outcomes to be evaluated but unlikely to achieve significance include ED revisits, intravenous rehydration, and hospitalization. Additional analyses involving these outcomes will include linear and logistic regression models that adjust for possible effects of baseline characteristics.

Side Effect Profile: The proportions of children experiencing any **side effect**, as reported by the caregivers, will be compared between groups using the Mantel-Haenszel test, stratified by site. The analysis will evaluate the presence/absence of side effects, as an aggregate outcome variable.

Mechanism-Fecal Secretory IgA: To test for a difference in **fecal secretory IgA** the Wilcoxon rank-sum test will be performed. As this is a mechanistic outcome and the motivation of its study is distinct from other outcomes, the test will be performed at the 0.05 level. Data will be analyzed to determine if fecal secretory IgA levels 5 days and 4 weeks after initiation of treatment are higher amongst children treated with probiotic than those treated with placebo. Fecal sIgA data will also be analyzed by outcome, comparing levels amongst those with mild disease to those with moderate-severe disease.

Pathogen Load Quantification: To determine if a 5-day probiotic treatment course administered to children with AGE results in **pathogen-specific reductions in stool pathogen load.** Benefit is defined as the difference in stool pathogen load between Days #0, #5, and #28. The analysis will employ a 2-way ANOVA followed by multivariable linear regression models adjusted for pathogen, interaction and important covariates (e.g. age, sex, baseline Modified Vesikari Scale score, baseline pathogen load, antibiotic use, increase in fecal sIgA). The analysis will determine if reduction in pathogen-specific load is independently related to treatment and pathogen. Based on the distribution of the reduction in pathogen-specific loads, the mean or median reductions will be explored in relation to pathogen, comparing:

1. **Pathogen-group:** virus vs. bacteria
2. **Viral agent:** rotavirus vs. norovirus vs. adenovirus
3. **Bacterial agent:** *Campylobacter* vs. *Salmonella* (only ones anticipated to have sufficient numbers)

Since there is the potential that clinical response, pathogen load reduction, and fecal sIgA are related outcomes, we will explore the overall simultaneous change in the means of the outcomes due to treatment arm by performing a Hotelling's t-test on the three response vectors (i.e. differences in Modified Vesikari Scale score, and the Days #0 and 5 and Days #0 and 28 changes in infectious load and fecal sIgA).

2.18 WHAT IS THE PROPOSED FREQUENCY OF ANALYSES?

The **Data Safety Monitoring Committee (DSMC)** will meet after 200 and 500 patients to review enrollment, study procedures, form completion, data quality, loss to follow-up, drop-in rate, and interim safety and efficacy results. The analyses will test the hypothesis that the probability of developing moderate to severe AGE in the probiotic arm is equal to that in the placebo arm. Conservative O'Brien-Fleming monitoring boundaries, implemented using the Lan-DeMets alpha-spending function approach,

will be used as guidelines for early stopping for safety or efficacy. Based on trends and adverse events, the DSMC may decide to meet sooner than planned using boundaries adjusted accordingly. Because this trial involves children under the age of 6 months, the DSMC has approved a plan to complete an interim safety analysis on the first 20 subjects enrolled under 6 months of age. All serious adverse events will be reported within 24 hours to the DSMC and based on these reports; the DSMC may decide to conduct a safety analysis before the full 20 subjects have been enrolled in this age group. Otherwise, a blinded analysis will be conducted after the 20 subjects < 6 months of age have been enrolled. This data will be unblinded if the DSMC deems it necessary to conduct an unblinded interim safety analysis. The results of this analysis will be communicated to the NNHPD branch of Health Canada at the discretion of the DSMC chair should any concerns be identified.

2.19 ARE THERE ANY PLANNED SUBGROUP ANALYSES? (1) The presence of a MVS ≥ 9 will be analyzed by **(i)** age < 1 year, **(ii)** breast-feeding status, **(iii)** antibiotic usage and **(iv)** protocol compliance. **(2)** Duration of vomiting will be analyzed only in those patients who have ≥ 3 episodes of vomiting in the 24 hours prior to enrollment. **(3)** Daycare and work absenteeism will only be analyzed for children who attend daycare and caregivers who work. A subgroup analysis will be performed for children with **(4)** rotavirus infection by adding an interaction term between treatment and rotavirus positivity in a logistic regression model. The independent variables in the model will be **(i)** treatment group, **(ii)** rotavirus positivity (yes/no) and **(iii)** the interaction between treatment group and rotavirus positivity. Universal rotavirus vaccination does not exist in Canada with the decision being made individually by each province based on the expense as well as feasibility.[192,193] At present it is included in the provincial schedules in Quebec and Ontario but not in Nova Scotia or Alberta. The varying use of the vaccine and our goal to identify etiologic agents and to conduct sub-analyses will yield very important information related to probiotic use in the presence/absence of rotavirus vaccination. **(5)** Fecal sIgA levels will be sub-analyzed based on the mother's breast-feeding status.

2.20 DATA SHARING

Participant data will be stored in an online electronic data capture system (REDCap). Collected data will be downloaded at the coordinating centre in Calgary, Alberta Canada. In order to complete the planned subgroup and economic analyses, a de-identified dataset containing only the variables required will be shared with collaborating institutions. The planned economic analyses will be performed by the Program for Assessment of Technology in Health (PATH) Research Institute at McMaster University (Appendix 9-Economic Analysis Plan) located in Hamilton, Ontario Canada. Data will also be shared with the University of Utah Data Coordinating Center (DCC) located in Salt Lake City, Utah USA. The DCC will integrate our study data with those from a companion clinical trial taking place in the United States (co-PIs Dr. Stephen Freedman and Dr. David Schnadower). Integration of data will allow for additional analyses to be performed that would be underpowered for either study to perform them in isolation.

Pathogen Load Quantification Data: Specimens are received de-identified by the processing labs. Results of the pathogen load testing performed by Drs. Xiao-Li Pang, Linda Chui, and Bonita Lee will be compiled and entered in to a simple database. The de-identified database will be sent to the coordinating centre in Calgary, Alberta using a secure email service (Alberta Health Services). These results may be shared with the DCC in Salt Lake City, Utah.

Fecal Secretory IgA Data: Fecal sIgA results will be entered in to a simple database. The database will be encrypted and sent to the Principal Investigator at the coordinating centre via institutional email. All participant results will be de-identified. De-identified specimens are received at the lab of Dr. Sherman located at the Hospital for Sick Children. These results may be shared with the DCC in Salt Lake City, Utah.

2.21 HAS ANY PILOT STUDY BEEN CARRIED OUT USING THIS DESIGN?

The participating research team members and PERC network have extensive experience conducting clinical research.[4,82,91,147,191] The network has monthly conference calls and the executive meets several times per year. Dr. Freedman, the Vice-Chair of PERC, has successfully completed and published several gastroenteritis clinical trials,[66,194] with publications in BMJ[122] and NEJM.[79] He additionally led a 50 patient multicentre pilot study employing Lacidofil which provided promising preliminary data, evaluated the feasibility of the current proposal and identified potential problems. The pilot included a placebo group and two dosages: 4 x 10^9 CFU/day and 8 x 10^9 CFU/day. It did not detect a trend toward increased side effects in the 8 x 10^9 CFU/day arm; hence, to ensure our study has the optimal ability to answer the primary question, the 8 x 10^9 CFU/day dose will be used. Overall, 91% of all doses dispensed were administered. Key information data provided by the pilot were: **(1)** the safety of high dose Lacidofil, **(2)** anticipated recruitment and compliance estimates, **(3)** the revision of data collection forms, **(4)** the use of rectal swab for specimen collection (aside from sIgA), **(5)** the optimal rectal swab testing device, **(6)** day #5 instead of 7 compliance assessment, **(7)** modified follow-up protocol to minimize loss to follow-up, and **(8)** proved our ability to obtain Health Canada approval.

3.0 TRIAL MANAGEMENT

3.1 WHAT ARE THE ARRANGEMENTS FOR DAY-TO-DAY MANAGEMENT OF THE TRIAL? (APPENDIX 13) WCHRI, based at the University of Alberta, will act as a central repository for all study data. Staffing will include a project manager, a medical informatics specialist and an assistant. WCHRI will be responsible for the provision of data collection technology and clinical data management services. WCHRI's staff has extensive experience and expertise in collecting data using REDCap software and managing study data in accordance with Good Clinical Practice requirements including the use of qualified and trained study personnel, study monitoring, standard operating procedures, validated software, data audit trails, and quality assurance. Study participating sites will retain the option of using the developed REDCap database as the primary method of data collection and storage. Due to the extensive validation completed by WCHRI, data can be obtained from the patient and then directly entered into the secure REDCap database via an electronic tablet (e.g. iPad®). Study sites may also collect data on paper case report forms, which would then be transcribed into the REDCap database. For all study data collected, source documentation will be defined in the Manual of Operations. The Alberta Children's Hospital (the PI's institution) serving as the coordinating centre, will be in constant communication with WCHRI, and will be responsible for study training, monitoring, and progress. Drs. Willan and Nettel-Aguirre will supervise all data analyses. Dr. Freedman will take overall responsibility for the study. Site Investigators and Clinical Research Assistants/Nurses will share responsibilities including day to day activities, payroll, study promotion, contacting caregivers, and reviewing charts.

Research Ethics Board (REB) and Health Canada approvals will be obtained. All ED physicians and nurses will be educated regarding the study and Clinical Research Assistants/Nurses will be trained. Sites have committed to having Clinical Research Assistants or Clinical Research Nurses present 75 hours/week during peak season and volume periods (7 months/year). Their presence will maximize study enrollment by continuously reminding physicians about the study and enrolling eligible children. Participating institutions all have significant infrastructure in place and will use a variety of methods to optimize coverage while minimizing costs including Clinical Research Assistants or Nurses covering multiple studies and volunteer programs (e.g. www.sickkids.ca/HealthcareProfessionalsandStudents/clinical-research/index.html).

The AHS Research Pharmacy will ship the study drug in batches to the participating institutions. The AHS Research Pharmacy will also maintain a batch of sachets which have not been randomized to be

sent to high recruiting sites. Collaborating pharmacies will be blinded to study drug and will be responsible for storage and providing study kits to the site Clinical Research Assistants/Nurses. Regular e-mail, weekly teleconferencing for the first 6 weeks of the trial, and monthly conference calls will be used to monitor start up and to obtain updates on recruitment and issues arising. Real-time data entry will facilitate an ongoing data cleaning plan. Double data entry will be employed on a random sampling of subjects at various time points throughout the study to ensure the data collected is accurate and is being recorded properly.

Drs. Pang, Louie and Chui will take responsibility for microbiologic testing, specimen storage and data management at ProvLab AB. They have extensive experience managing stool specimens and will correspond with the study team at ACH on a weekly basis.

3.2 WHAT WILL BE THE ROLE OF EACH INVESTIGATOR AND COLLOBORATOR? This study, under the umbrella of PERC brings together North American investigators with transdisciplinary expertise. Dr. Freedman who has expertise in AGE research,[1,4,7,19,66,122,131,132,194-197] recently reported[79] that ondansetron, an antiemetic agent, is effective in pediatric AGE. It is now routinely used to reduce the need for intravenous hydration and hospitalization.[80,132,198-200] Dr. Gorelick, a clinical epidemiologist,[201-204] with significant network research experience,[205-209] has provided senior guidance and high level input from a large research think-tank in the United States (Pediatric Emergency Care Applied Research Network-PECARN). Dr. Schuh[83,186] has successfully completed 15 pediatric ED RCTs and has guided the study since its inception. Dr. Johnson, [82,186] who has multicentre RCT experience has served as a resource regarding operational issues and will guide KT[210-212] efforts. Dr. Schnadower has led efforts to conduct a similar study in the United States and has served as a liaison with the Pediatric Emergency Medicine Collaborative Research Committee (Dr. Freedman is a steering committee member). Site investigators will supervise the study at their respective institutions. Dr. Philip Sherman has experience with Lacidofil[69,71,213] and his laboratory will perform the fecal sIgA analyses.[96,97] Drs. Willan and Nettel-Aguirre, both PhD statisticians, and Mr. Goeree, a health economist, will perform the statistical and economic evaluations. Dr. Willan is extensively involved in methodologic research in the area of health economics and optimizing decision-making in health care research and policy.[214-216] **Dr. Nettel-Aguirre** (co-applicant) is a biostatistician with extensive experience in analyzing health outcomes and related data from large, complex, linked datasets and in designing healthcare studies.[217-221]

Microbiologic Team: **Dr. Yaron Finkelstein** (co-PA), a board-certified clinical pharmacologist and pediatric emergency medicine physician has conducted multiple RCTs exploring pharmacometrics and safety in infected pediatric[222,223] and general[224] populations in addition to pathogen-specific efficacy studies in infectious gastrointestinal diseases.[225,226] Drs. Freedman and Finkelstein have successfully collaborated on several pediatric AGE and clinical medication studies.[224,227-229] Our team includes **Drs. Xiao-li (Lilly) Pang and Bonita Lee** (co-applicants) who have collaborated extensively[167,230] and have developed numerous assays for virus detection,[163,164,169,230,231] and quantification.[166,232-234] They will share joint responsibility for all viral analyses. **Dr. Linda Chui** (co-applicant), who has done extensive work in the development of protocols for the molecular detection of non-traditional enteric bacteria (i.e. non-O157 STEC) employing real-time PCR,[235-239] will be responsible for the quantification of stool bacterial load which is a natural extension of her molecular work and expertise in this area.[232,237,240,241] **Dr. Marie Louie** (co-applicant), an infectious disease specialist and medical microbiologist with expertise investigating and managing the public health implications of enteric pathogens,[240,242,243] will lead knowledge translation efforts within the microbiology community.

Working with stool from children with norovirus (n=244) and rotavirus (n=102), our team (Pang, Lee) has developed and validated real-time quantitative PCR assays to measure enteric virus genomic nucleic

acid (NA) in stool (i.e. quantify stool viral load; see Appendix 3). A standard curve has been established employing known genomic copies of DNA fragments, which have then undergone 10-fold dilutions from a single copy to 1 x 10^8 copies. Our team (Chui) has established a bacterial DNA extraction protocol which yields high quality and quantity of DNA. This led to the whole-genome sequencing of 200 bacterial isolates which identified biomarkers for the development of amplification assays, both loop-mediated isothermal amplification (LAMP) and real-time polymerase chain reaction (PCR) assays. Both assays have excellent sensitivity and no evidence of cross reactivity has been observed. These quantitative assays have been correlated with colony forming unit (CFU) counts with crossing point values in the real-time PCR assay.

All team members will be aided by WCHRI, MICYRN, the Clinical Research Coordinator, and the site Clinical Research Assistants or Nurses. Each study site has a dedicated study research coordinator who is responsible for organizing the conduct of the study at their respective institutions. Supporting the pathogen effectiveness work, lab research technologists have extensive experience with specimen processing, handling, storage, and testing.

3.3 DESCRIBE THE TRIAL STEERING COMMITTEE AND THE DATA SAFETY AND MANAGEMENT COMMITTEE. ***Trial Steering Committee:*** The advisory panel has included knowledge users, caregivers, pediatricians, emergency medicine physicians, gastroenterologists, and infectious disease physicians. The protocol has been revised based on guidance provided by the PERC and PECARN networks. Non-research team members who have had extensive input include clinicians, statisticians, ethicists, and coordinators with multicentre research expertise. Official committee members have included senior clinical research team members (Drs. Gorelick, Schuh, Johnson), Dr. Sherman, a Canada Research Chair in Gastrointestinal Disease (selection of probiotic agent, dose, duration of therapy, and planned translational studies), Dr. Kuppermann,[181,244-248] the past-Chair of PECARN, Dr. Dean,[249-253] expert in conduct of multicentre network research, and Dr. Plint, the Chair of PERC. This has ensured that the study will answer important questions that can readily be applied by these leading KT research networks.[210-212] ***Data Safety Monitoring Board (section 2.18 also):*** There will be an independent monitoring committee consisting of a biostatistician (Nick Barrowman, PhD-Ottawa), and two physicians with RCT expertise (Drs. Mark Roback–Minnesota and Terry Klassen (Chair) - Winnipeg). This committee will be independent of the investigators and will be advised of all adverse events.

3.4 ADVERSE EVENT REPORTING

Adverse Event (AE): An adverse event is any unfavorable or unintended clinical or other occurrence during the study period that may or may not be the result of participation in the research study.

Expected Adverse Drug Reactions/Events

These include the following as they are part of the natural history of the underlying disease process:

- Hospitalization
- Future health care provider visit, ED return visit
- IV rehydration
- Abdominal pain, distension
- Vomiting, diarrhea, fever, flatulence

Because expected adverse events are part of the natural history of acute gastroenteritis and diarrheal illness in children, they will not need to be reported as Adverse Events. This information will be recorded in normal study data collection processes.

Serious Adverse Events

Any Serious Adverse Event (SAE) that occurs after the first sachet administered will be reported to the Research Ethics Board (REB) and the study subject will be followed until the conclusion of the event.

A SAE is defined as:

- Results in death.
- Is life-threatening. This refers to an event in which the patient was at immediate risk of death; it does not refer to an event that might have caused death had it been more severe.
- Results in a persistent or significant disability/incapacity
- Results in a congenital anomaly/birth defect
- Is medically significant. Important medical events that may not result in death, be life-threatening, or require hospitalization may be considered SAEs when, based upon appropriate medical judgment, may jeopardize the patient and may require medical or surgical intervention to prevent one of the outcomes listed in this definition.

In addition, any serious adverse reaction to the natural health product will be reported to the Natural Health Product Directorate (NHPD).

Adverse Event Reports

For unexpected adverse events, we will inform the REB, in addition to the clinical chief of the ED, and the external sponsor within 7 days of learning of the event, if applicable and deemed necessary by the Principal Investigator.

For unexpected SAEs, we will inform the REB, in addition to the clinical chief of the ED, and the external sponsor within 24 hours of learning of the event (by AE form, telephone or email). The SAE information will be sent even if the information is incomplete. A complete follow-up AE report will be submitted as soon as possible but no later than 7 days after the initial reporting.

Collaborating Study Sites

The principal investigator or delegate will also submit to the University of Calgary REB information received from other sites. Conversely, serious adverse events that occur at The Alberta Children's Hospital (The University of Calgary) will be communicated by the principal investigator to collaborating sites, as their local requirements dictate. To ensure that data remains confidential and unbiased, a medical monitor will be appointed at the sponsoring institution (The University of Calgary). The medical monitor will be an Emergency Department physician with expertise in clinical research. The medical monitor will review adverse event information from collaborating study sites, in lieu of the principal investigator. The principal investigator (as the sponsor) will still maintain the responsibility of reviewing any Serious Adverse Events occurring at any of the participating study sites.

Adverse Event Coding:

Adverse Event (AE) data will be reviewed by trained staff and coded using the Medical Dictionary for Regulatory Activity (MedDRA - https://www.meddra.org/) system. Adverse Event data will be collected from participants at the time of the event. MedDRA coding will be assigned to each event at the end of the recruiting period.

Health Canada (Natural Health Product Directorate) Reporting

Adverse drug reactions (ADR) that are both serious and unexpected are subject to expedited reporting to Health Canada (NHPD) *by the sponsor*. These include reactions;

- Where it is fatal or life-threatening, immediately where possible and, in any event, within 7 days after becoming aware of the information

- A complete follow up report within 8 days which includes an assessment of the importance and implication of any findings including relevant previous experience with the same or similar drugs
- Where it is neither fatal nor life-threatening within 15 days after becoming aware of the information

Each ADR which is subject to expedited reporting will be reported individually in accordance with the data element(s) specified in Section 78 of the *NHP Regulations,* ICH Guidance Document *E2A: Clinical Safety Data Management: Definitions and Standards for Expedited Reporting.*

Emergency Unblinding

Un-blinding should only occur in the event that there is clinical concern regarding the possibility of bacteremia/septicemia or when it is felt by the treating physician that unblinding would alter the clinical care being provided. All patients whose therapy is intentionally un-blinded will discontinue the experimental therapy. Un-blinding should only occur when future clinical treatment of the patient will depend on prior treatment administered. Approval from the principal investigator or designate will be obtained prior to un-blinding. If the principal investigator cannot be reached, the un-blinding can be performed and the principal investigator informed within 24 hours via e-mail or telephone call. Accidental and intentional un-blinding will be documented and reported and the subject will be withdrawn from the study.

3.5 PREMATURE WITHDRAWL/DISCONTINUATION CRITERIA

The subjects retain the right to withdraw from the study at any time, although withdrawal from the study is strongly discouraged after the subject has been enrolled.

Every effort will be made to contact all subjects for follow-up as scheduled. Subjects will be withdrawn from the study if:

1. After enrollment they are determined to meet any of the exclusion criteria
2. If the subject is admitted to an intensive care unit
3. If it is deemed by the treating physician that the child's health may be jeopardized by continued participation in the study
4. The patient's caregivers wish to withdraw their child for whatever reason

If the patient's caregiver chooses to withdraw their child from the study, they will be provided with a choice regarding their exit from the study:

1. The caregiver may choose to withdraw the child from the study, as well as all data collected from their child's participation in the study
2. The caregiver may choose to withdraw their child from the study; however they will allow continued use of study data collected from their child.

3.6 RECORD KEEPING

The data produced from this study will be stored in a secure, locked location. Only members of the research team will have access to the data. Following completion of the research study the data will be stored and kept for a minimum of 25 years. The data will then be destroyed in accordance with the University of Calgary and Tri Council destruction policy for clinical trial documentation.

1. Freedman SB, Steiner M, Chan K. Oral Ondansetron Administration in Emergency Departments to Children with Gastroenteritis: An Economic Analysis. PLoS Med 7(10): e1000350 doi:101371/journalpmed1000350 2010.
2. Senecal M, Brisson M, Lebel MH, et al. Measuring the Impact of Rotavirus Acute Gastroenteritis Episodes (MIRAGE): A prospective community-based study. The Canadian journal of infectious diseases & medical microbiology = Journal canadien des maladies infectieuses et de la microbiologie medicale / AMMI Canada 2008;19:397-404.
3. Le Saux N, Bettinger JA, Halperin SA, Vaudry W, Scheifele DW. Substantial morbidity for hospitalized children with community-acquired rotavirus infections: 2005-2007 IMPACT surveillance in Canadian hospitals. Pediatr Infect Dis J 2010;29:879-82.
4. Freedman SB, Gouin S, Bhatt M, et al. Prospective assessment of practice pattern variations in the treatment of pediatric gastroenteritis. Pediatrics 2011;127:e287-95.
5. Carroll AE, Downs SM. Improving decision analyses: parent preferences (utility values) for pediatric health outcomes. J Pediatr 2009;155:21-5, 5 e1-5.
6. Dennehy PH. Transmission of rotavirus and other enteric pathogens in the home. Pediatr Infect Dis J 2000;19:S103-5.
7. Freedman SB, Eltorky M, Gorelick M, Pediatric Emergency Research Canada Gastroenteritis Study G. Evaluation of a gastroenteritis severity score for use in outpatient settings. Pediatrics 2010;125:e1278-85.
8. Mast TC, DeMuro-Mercon C, Kelly CM, Floyd LE, Walter EB. The impact of rotavirus gastroenteritis on the family. BMC Pediatr 2009;9:11.
9. Salminen S, Ouwehand A, Benno Y, Lee YK. Probiotics: how should they be defined? Trend Food Sci Technol 1999;10:107-10.
10. National Center for Complementary and Alternative Medicine at the National Institutes of Health. Oral Probiotics: An Introduction. 2011. (Accessed August 27, 2012, at http://nccam.nih.gov/health/probiotics/introduction.htm.)
11. Salari P, Nikfar S, Abdollahi M. A meta-analysis and systematic review on the effect of probiotics in acute diarrhea. Inflamm Allergy Drug Targets 2012;11:3-14.
12. Allen SJ, Martinez EG, Gregorio GV, Dans LF. Probiotics for treating acute infectious diarrhoea. Cochrane Database of Systematic Reviews 2010;11:CD003048.
13. Hom J. Do probiotics reduce the duration and symptoms of acute infectious diarrhea? Ann Emerg Med 2011;58:445-6.
14. King CK, Glass R, Bresee JS, Duggan C. Managing acute gastroenteritis among children: oral rehydration, maintenance, and nutritional therapy. MMWR Recomm Rep 2003;52:1-16.
15. D'Souza AL, Rajkumar C, Cooke J, Bulpitt CJ. Probiotics in prevention of antibiotic associated diarrhoea: meta-analysis. BMJ (Clinical Research Ed) 2002;324:1361.
16. Reid G, Jass J, Sebulsky MT, McCormick JK. Potential uses of probiotics in clinical practice. Clin Microbiol Rev 2003;16:658-72.
17. Li ST, Klein EJ, Tarr PI, Denno DM. Parental management of childhood diarrhea. Clin Pediatr (Phila) 2009;48:295-303.
18. Vernacchio L, Vezina RM, Mitchell AA, Lesko SM, Plaut AG, Acheson DW. Diarrhea in American infants and young children in the community setting: incidence, clinical presentation and microbiology. Pediatr Infect Dis J 2006;25:2-7.
19. Freedman SB, Sivabalasundaram V, Bohn V, Powell EC, Johnson DW, Boutis K. The treatment of pediatric gastroenteritis: a comparative analysis of pediatric emergency physicians' practice patterns. Acad Emerg Med 2011;18:38-45.
20. Rochon PA, Sekeres M, Hoey J, et al. Investigator experiences with financial conflicts of interest in clinical trials. Trials 2011;12:9.

21. Crocetti MT, Amin DD, Scherer R. Assessment of risk of bias among pediatric randomized controlled trials. Pediatrics 2010;126:298-305.
22. Martinez-Castaldi C, Silverstein M, Bauchner H. Child versus adult research: the gap in high-quality study design. Pediatrics 2008;122:52-7.
23. Cohen E, Goldman RD, Ragone A, et al. Child vs adult randomized controlled trials in specialist journals: a citation analysis of trends, 1985-2005. Archives of Pediatrics and Adolescent Medicine 2010;164:283-8.
24. Freedman SB. Parental probiotic knowledge. Unpublished raw data. The Hospital for Sick Children; 2010.
25. Navaneethan U, Giannella RA. Mechanisms of infectious diarrhea. Nat Clin Pract Gastroenterol Hepatol 2008;5:637-47.
26. Corr SC, Hill C, Gahan CG. Understanding the mechanisms by which probiotics inhibit gastrointestinal pathogens. Adv Food Nutr Res 2009;56:1-15.
27. National Collaborating Centre for Women's and Children's Health. Diarrhoea and vomiting caused by gastroenteritis: diagnosis, assessment and management in children younger than 5 years: Commissioned by the National Institute for Health and Clinical Excellence; Available at: http://www.nice.org.uk/guidance/cg84/resources/cg84-diarrhoea-and-vomiting-in-children-under-5-full-guideline2. Accessed October 15, 2014; April 2009; London, UK.
28. Weizman Z. Probiotics use in childhood acute diarrhea: a web-based survey. J Clin Gastroenterol 2011;45:426-8.
29. Vandenplas Y, Salvatore S, Viera M, Devreker T, Hauser B. Probiotics in infectious diarrhoea in children: are they indicated? Eur J Pediatr 2007.
30. Katan MB. Why the European Food Safety Authority was right to reject health claims for probiotics. Benef Microbes 2012;3:85-9.
31. Vero V, Gasbarrini A. The EFSA health claims 'learning experience'. Int J Food Sci Nutr 2012;63 Suppl 1:14-6.
32. Flynn A. Scientific substantiation of health claims in the EU. Proc Nutr Soc 2012;71:120-6.
33. Heimbach JT. Health-benefit claims for probiotic products. Clin Infect Dis 2008;46 Suppl 2:S122-4; discussion S44-51.
34. Global probiotics market worth US$32.6 billion by 2014. 2012. (Accessed July 27th 2012, 2012, at http://www.marketsandmarkets.com/PressReleases/global-probiotics-market-worth-US-32-billion-by-2014.asp.)
35. Coppens P, da Silva MF, Pettman S. European regulations on nutraceuticals, dietary supplements and functional foods: a framework based on safety. Toxicology 2006;221:59-74.
36. Saldanha LG. US Food and Drug Administration regulations governing label claims for food products, including probiotics. Clin Infect Dis 2008;46 Suppl 2:S119-21; discussion S44-51.
37. von Wright A. Regulating the safety of probiotics--the European approach. Curr Pharm Des 2005;11:17-23.
38. Use of Lactobacillus GG in Children with Acute Gastroenteritis. 2011. (Accessed July 27th, 2012, at http://www.google.com/url?sa=t&rct=j&q=&esrc=s&source=web&cd=3&ved=0CFsQFjAC&url=http%3A%2F%2Fwww.cincinnatichildrens.org%2FWorkArea%2Flinkit.aspx%3FLinkIdentifier%3Did%26ItemID%3D88039%26libID%3D87727&ei=FK0SUJXFN4iirAH65oHYDQ&usg=AFQjCNE8lhSeTiAUcY3kmdemCHBt3HfmXQ.)
39. Guyatt G, Montori V, Devereaux PJ, Schunemann H, Bhandari M. Patients at the center: in our practice, and in our use of language. ACP journal club 2004;140:A11-2.
40. Johnston BC, Shamseer L, da Costa BR, Tsuyuki RT, Vohra S. Measurement issues in trials of pediatric acute diarrheal diseases: a systematic review. Pediatrics 2010;126:e222-31.

41. Das RR. Should Probiotics be Used in the Treatment of Acute Childhood Diarrhea? J Clin Gastroenterol 2012;46:526-7.
42. Guandalini S. Probiotics for children with diarrhea: an update. J Clin Gastroenterol 2008;42 Suppl 2:S53-7.
43. Coffin SE, Elser J, Marchant C, et al. Impact of acute rotavirus gastroenteritis on pediatric outpatient practices in the United States. Pediatr Infect Dis J 2006;25:584-9.
44. Szajewska H, Skorka A, Ruszczynski M, Gieruszczak-Bialek D. Meta-analysis: Lactobacillus GG for treating acute diarrhoea in children. Aliment Pharmacol Ther 2007;25:871-81.
45. Szymanski H, Pejcz J, Jawien M, Chmielarczyk A, Strus M, Heczko PB. Treatment of acute infectious diarrhoea in infants and children with a mixture of three Lactobacillus rhamnosus strains--a randomized, double-blind, placebo-controlled trial. Aliment Pharmacol Ther 2006;23:247-53.
46. Huang JS, Bousvaros A, Lee JW, Diaz A, Davidson EJ. Efficacy of probiotic use in acute diarrhea in children: a meta-analysis. Dig Dis Sci 2002;47:2625-34.
47. Van Niel CW, Feudtner C, Garrison MM, Christakis DA. Lactobacillus therapy for acute infectious diarrhea in children: a meta-analysis. Pediatrics 2002;109:678-84.
48. Vandenplas Y, Salvatore S, Vieira M, Devreker T, Hauser B. Probiotics in infectious diarrhoea in children: are they indicated? Eur J Pediatr 2007;166:1211-8.
49. Hoffman FA, Heimbach JT, Sanders ME, Hibberd PL. Executive summary: scientific and regulatory challenges of development of probiotics as foods and drugs. Clin Infect Dis 2008;46 Suppl 2:S53-7.
50. Szajewska H, Mrukowicz JZ. Use of probiotics in children with acute diarrhea. Paediatr Drugs 2005;7:111-22.
51. Nixon AF, Cunningham SJ, Cohen HW, Crain EF. The effect of Lactobacillus GG (LGG) on acute diarrheal illness in the pediatric emergency department (PED). Pediatric Academic Societies' 2010 Annual Meeting [abstract]. Vancouver, Canada; in press Pediatr Emerg Care.
52. Walker WA. Mechanisms of action of probiotics. Clin Infect Dis 2008;46 Suppl 2:S87-91; discussion S144-51.
53. Sarker SA, Fuchs GJ. The role of probiotics in the treatment and prevention of infectious diarrhea in children. In: Michail S, Sherman PM, eds. Proboitics in pediatric medicine. Totowa, NJ: Humana Press; 2009.
54. Tannock GW, Munro K, Harmsen HJ, Welling GW, Smart J, Gopal PK. Analysis of the fecal microflora of human subjects consuming a probiotic product containing Lactobacillus rhamnosus DR20. Applied and environmental microbiology 2000;66:2578-88.
55. Michail S, Abernathy F. Lactobacillus plantarum reduces the in vitro secretory response of intestinal epithelial cells to enteropathogenic Escherichia coli infection. J Pediatr Gastroenterol Nutr 2002;35:350-5.
56. Hooper LV, Wong MH, Thelin A, Hansson L, Falk PG, Gordon JI. Molecular analysis of commensal host-microbial relationships in the intestine. Science 2001;291:881-4.
57. Neish AS, Gewirtz AT, Zeng H, et al. Prokaryotic regulation of epithelial responses by inhibition of IkappaB-alpha ubiquitination. Science 2000;289:1560-3.
58. Pessi T, Sutas Y, Hurme M, Isolauri E. Interleukin-10 generation in atopic children following oral Lactobacillus rhamnosus GG. Clin Exp Allergy 2000;30:1804-8.
59. Majamaa H, Isolauri E. Probiotics: a novel approach in the management of food allergy. J Allergy Clin Immunol 1997;99:179-85.
60. Wolvers D, Antoine JM, Myllyluoma E, Schrezenmeir J, Szajewska H, Rijkers GT. Guidance for Substantiating the Evidence for Beneficial Effects of Probiotics: Prevention and Management of Infections by Probiotics. J Nutr 2010.
61. Canani RB, Cirillo P, Terrin G, et al. Probiotics for treatment of acute diarrhoea in children: randomised clinical trial of five different preparations. BMJ (Clinical Research Ed) 2007;335:340.

62. Food and Agriculture Organization of the United Nations, World Health Organization. Joint FAO/WHO expert consultation on evaluation of health and nutritional properties of probiotics in food including powder milk and live lactic acid bacteria2001.

63. Reid G, Kirjaivanen P. Taking probiotics during pregnancy. Are they useful therapy for mothers and newborns? Can Fam Physician 2005;51:1477-9.

64. Sanders M, Morelli L, Bush S. "Lactobacillus sporogenes" is not a *Lactobacillus* probiotic. ASM News 2001;67:385-6.

65. Sherman PM, Johnson-Henry KC, Yeung HP, Ngo PS, Goulet J, Tompkins TA. Probiotics reduce enterohemorrhagic Escherichia coli O157:H7- and enteropathogenic E. coli O127:H6-induced changes in polarized T84 epithelial cell monolayers by reducing bacterial adhesion and cytoskeletal rearrangements. Infection and Immunity 2005;73:5183-8.

66. Freedman SB, Couto M, Spooner L, Haladyn JK. The implementation of a gastroenteritis education program. Am J Emerg Med 2011;29:271-7.

67. Wine E, Gareau MG, Johnson-Henry K, Sherman PM. Strain-specific probiotic (Lactobacillus helveticus) inhibition of Campylobacter jejuni invasion of human intestinal epithelial cells. FEMS Microbiol Lett 2009;300:146-52.

68. Johnson-Henry KC, Mitchell DJ, Avitzur Y, Galindo-Mata E, Jones NL, Sherman PM. Probiotics reduce bacterial colonization and gastric inflammation in H. pylori-infected mice. Dig Dis Sci 2004;49:1095-102.

69. Johnson-Henry KC, Nadjafi M, Avitzur Y, et al. Amelioration of the effects of Citrobacter rodentium infection in mice by pretreatment with probiotics. The Journal of infectious diseases 2005;191:2106-17.

70. Gareau MG, Jury J, MacQueen G, Sherman PM, Perdue MH. Probiotic treatment of rat pups normalises corticosterone release and ameliorates colonic dysfunction induced by maternal separation. Gut 2007;56:1522-8.

71. Zareie M, Johnson-Henry K, Jury J, et al. Probiotics prevent bacterial translocation and improve intestinal barrier function in rats following chronic psychological stress. Gut 2006;55:1553-60.

72. Rijkers GT, Bengmark S, Enck P, et al. Guidance for Substantiating the Evidence for Beneficial Effects of Probiotics: Current Status and Recommendations for Future Research. J Nutr 2010.

73. Reid G, Hammond JA. Probiotics. Some evidence of their effectiveness. Canadian Family Physician 2005;51:1487-93.

74. Szajewska H, Mrukowicz JZ. Probiotics in the treatment and prevention of acute infectious diarrhea in infants and children: a systematic review of published randomized, double-blind, placebo-controlled trials. J Pediatr Gastroenterol Nutr 2001;33 Suppl 2:S17-25.

75. Huang JS, Bousvaros A, Lee JW, Diaz A, Davidson EJ. Efficacy of probiotic use in acute diarrhea in children: a meta-analysis. Dig Dis Sci 2002;47:2625-34.

76. Dinleyici EC, Eren M, Ozen M, Yargic ZA, Vandenplas Y. Effectiveness and safety of Saccharomyces boulardii for acute infectious diarrhea. Expert Opin Biol Ther 2012;12:395-410.

77. Szajewska H, Mrukowicz JZ. Use of probiotics in children with acute diarrhea. Paediatr Drugs 2005;7:111-22.

78. Pappano D, Conners G, McIntosh S, Humiston S, Roma D. Sources of knowledge transfer among primary care pediatric health care providers. Clin Pediatr (Phila) 2008;47:930-4.

79. Freedman SB, Adler M, Seshadri R, Powell EC. Oral ondansetron for gastroenteritis in a pediatric emergency department. N Engl J Med 2006;354:1698-705.

80. Sturm JJ, Hirsh DA, Schweickert A, Massey R, Simon HK. Ondansetron Use in the Pediatric Emergency Department and Effects on Hospitalization and Return Rates: Are We Masking Alternative Diagnoses? Ann Emerg Med 2009.

81. Johnson D. Croup. Clin Evid (Online) 2009;2009.

82. Bjornson CL, Klassen TP, Williamson J, et al. A randomized trial of a single dose of oral dexamethasone for mild croup. The New England journal of medicine 2004;351:1306-13.
83. Schuh S, Reisman J, Alshehri M, et al. A comparison of inhaled fluticasone and oral prednisone for children with severe acute asthma. The New England journal of medicine 2000;343:689-94.
84. Vuillermin P, South M, Robertson C. Parent-initiated oral corticosteroid therapy for intermittent wheezing illnesses in children. Cochrane Database of Systematic Reviews 2006;3:CD005311.
85. Reid G. Safety of lactobacillus strains as probiotic agents. Clin Infect Dis 2002;35:349-50.
86. Land MH, Rouster-Stevens K, Woods CR, Cannon ML, Cnota J, Shetty AK. Lactobacillus sepsis associated with probiotic therapy. Pediatrics 2005;115:178-81.
87. Kunz AN, Noel JM, Fairchok MP. Two cases of Lactobacillus bacteremia during probiotic treatment of short gut syndrome. J Pediatr Gastroenterol Nutr 2004;38:457-8.
88. De Groote MA, Frank DN, Dowell E, Glode MP, Pace NR. Lactobacillus rhamnosus GG bacteremia associated with probiotic use in a child with short gut syndrome. Pediatr Infect Dis J 2005;24:278-80.
89. Moher D, Hopewell S, Schulz KF, et al. CONSORT 2010 explanation and elaboration: updated guidelines for reporting parallel group randomised trials. BMJ (Clinical Research Ed) 2010;340:c869.
90. Schulz KF, Altman DG, Moher D. CONSORT 2010 statement: updated guidelines for reporting parallel group randomised trials. BMJ (Clinical Research Ed) 2010;340:c332.
91. Plint AC, Johnson DW, Patel H, et al. Epinephrine and dexamethasone in children with bronchiolitis. N Engl J Med 2009;360:2079-89.
92. Chapman CM, Gibson GR, Rowland I. Health benefits of probiotics: are mixtures more effective than single strains? Eur J Nutr 2011;50:1-17.
93. Cabana MD, Shane AL, Chao C, Oliva-Hemker M. Probiotics in primary care pediatrics. Clin Pediatr (Phila) 2006;45:405-10.
94. Tlaskal P, Schramlova J, Kokesova A, et al. Probiotics in the treatment of diarrheal disease of viral etiology in children. NAFAS 2005;3:25-8.
95. Szajewska H, Setty M, Mrukowicz J, Guandalini S. Probiotics in gastrointestinal diseases in children: hard and not-so-hard evidence of efficacy. J Pediatr Gastroenterol Nutr 2006;42:454-75.
96. Gao Y, Sherman PM, Sun Y, Li D. Multiplexed high-throughput electrokinetically-controlled immunoassay for the detection of specific bacterial antibodies in human serum. Analytica Chimica Acta 2008;606:98-107.
97. Lin FY, Gao Y, Li D, Sherman PM. Development of microfluidic-based heterogeneous immunoassays. Front Biosci (Schol Ed) 2010;2:73-84.
98. Gorelick MH, Shaw KN, Murphy KO. Validity and reliability of clinical signs in the diagnosis of dehydration in children. Pediatrics 1997;99:E6.
99. Lewis JA, Machin D. Intention to treat--who should use ITT? Br J Cancer 1993;68:647-50.
100. Marshall M, Lockwood A, Bradley C, Adams C, Joy C, Fenton M. Unpublished rating scales: a major source of bias in randomised controlled trials of treatments for schizophrenia. Br J Psychiatry 2000;176:249-52.
101. World Health Organization. The treatment of diarrhoea: A manual for physicians and other senior health workers. 4th revision.: WHO Press; 2005.
102. Shornikova AV, Casas IA, Isolauri E, Mykkanen H, Vesikari T. Lactobacillus reuteri as a therapeutic agent in acute diarrhea in young children. J Pediatr Gastroenterol Nutr 1997;24:399-404.
103. Villarruel G, Rubio DM, Lopez F, et al. Saccharomyces boulardii in acute childhood diarrhoea: a randomized, placebo-controlled study. Acta Paediatr 2007;96:538-41.
104. Brandt CD, Kim HW, Rodriguez WJ, et al. Pediatric viral gastroenteritis during eight years of study. J Clin Microbiol 1983;18:71-8.
105. Munoz P, Bouza E, Cuenca-Estrella M, et al. Saccharomyces cerevisiae fungemia: an emerging infectious disease. Clin Infect Dis 2005;40:1625-34.

106. Riquelme AJ, Calvo MA, Guzman AM, et al. Saccharomyces cerevisiae fungemia after Saccharomyces boulardii treatment in immunocompromised patients. J Clin Gastroenterol 2003;36:41-3.
107. Besselink MG, van Santvoort HC, Buskens E, et al. Probiotic prophylaxis in predicted severe acute pancreatitis: a randomised, double-blind, placebo-controlled trial. Lancet 2008;371:651-9.
108. Johnston BC, Supina AL, Vohra S. Probiotics for pediatric antibiotic-associated diarrhea: a meta-analysis of randomized placebo-controlled trials. CMAJ 2006;175:377-83.
109. Fruhwirth M, Heininger U, Ehlken B, et al. International variation in disease burden of rotavirus gastroenteritis in children with community- and nosocomially acquired infection. Pediatr Infect Dis J 2001;20:784-91.
110. Givon-Lavi N, Greenberg D, Dagan R. Comparison between two severity scoring scales commonly used in the evaluation of rotavirus gastroenteritis in children. Vaccine 2008;26:5798-801.
111. Ruiz-Palacios GM, Perez-Schael I, Velazquez FR, et al. Safety and efficacy of an attenuated vaccine against severe rotavirus gastroenteritis. The New England journal of medicine 2006;354:11-22.
112. Madhi SA, Cunliffe NA, Steele D, et al. Effect of human rotavirus vaccine on severe diarrhea in African infants. The New England journal of medicine 2010;362:289-98.
113. Ruuska T, Vesikari T. Rotavirus disease in Finnish children: use of numerical scores for clinical severity of diarrhoeal episodes. Scand J Infect Dis 1990;22:259-67.
114. Iturriza Gomara M, Simpson R, Perault AM, et al. Structured surveillance of infantile gastroenteritis in East Anglia, UK: incidence of infection with common viral gastroenteric pathogens. Epidemiol Infect 2008;136:23-33.
115. Tapia MD, Armah G, Breiman RF, et al. Secondary efficacy endpoints of the pentavalent rotavirus vaccine against gastroenteritis in sub-Saharan Africa. Vaccine 2012;30 Suppl 1:A79-85.
116. Feikin DR, Laserson KF, Ojwando J, et al. Efficacy of pentavalent rotavirus vaccine in a high HIV prevalence population in Kenya. Vaccine 2012;30 Suppl 1:A52-60.
117. Breiman RF, Zaman K, Armah G, et al. Analyses of health outcomes from the 5 sites participating in the Africa and Asia clinical efficacy trials of the oral pentavalent rotavirus vaccine. Vaccine 2012;30 Suppl 1:A24-9.
118. Huppertz HI, Forster J, Heininger U, Roos R, Neumann HU, Hammerschmidt T. The parental appraisal of the morbidity of diarrhea in infants and toddlers (PAMODI) survey. Clin Pediatr (Phila) 2008;47:363-71.
119. Diez-Domingo J, Patrzalek M, Cantarutti L, et al. The impact of childhood acute rotavirus gastroenteritis on the parents' quality of life: prospective observational study in European primary care medical practices. BMC Pediatr 2012;12:58.
120. Mackenzie A, Barnes G, Shann F. Clinical signs of dehydration in children. Lancet 1989;2:605-7.
121. Yee EL, Staat MA, Azimi P, et al. Burden of rotavirus disease among children visiting pediatric emergency departments in Cincinnati, Ohio, and Oakland, California, in 1999-2000. Pediatrics 2008;122:971-7.
122. Freedman SB, Parkin PC, Willan AR, Schuh S. Rapid versus standard intravenous rehydration in paediatric gastroenteritis: pragmatic blinded randomised clinical trial. BMJ 2011;343:d6976.
123. Vesikari T, Ruuska T, Delem A, Andre FE, Beards GM, Flewett TH. Efficacy of two doses of RIT 4237 bovine rotavirus vaccine for prevention of rotavirus diarrhoea. Acta Paediatr Scand 1991;80:173-80.
124. Joensuu J, Koskenniemi E, Pang XL, Vesikari T. Randomised placebo-controlled trial of rhesus-human reassortant rotavirus vaccine for prevention of severe rotavirus gastroenteritis. Lancet 1997;350:1205-9.
125. Cicek C, Karatas T, Altuglu I, Koturoglu G, Kurugol Z, Bilgic A. Comparison of ELISA with shell vial cell culture method for the detection of human rotavirus in fecal specimens. New Microbiol 2007;30:113-8.

126. Binka FN, Anto FK, Oduro AR, et al. Incidence and risk factors of paediatric rotavirus diarrhoea in northern Ghana. Trop Med Int Health 2003;8:840-6.

127. Guarino A, Canani RB, Spagnuolo MI, Albano F, Di Benedetto L. Oral bacterial therapy reduces the duration of symptoms and of viral excretion in children with mild diarrhea. J Pediatr Gastroenterol Nutr 1997;25:516-9.

128. Isolauri E, Juntunen M, Rautanen T, Sillanaukee P, Koivula T. A human Lactobacillus strain (Lactobacillus casei sp strain GG) promotes recovery from acute diarrhea in children. Pediatrics 1991;88:90-7.

129. Rosenfeldt V, Michaelsen KF, Jakobsen M, et al. Effect of probiotic Lactobacillus strains in young children hospitalized with acute diarrhea. Pediatr Infect Dis J 2002;21:411-6.

130. Reid G. Microbiology: Categorize probiotics to speed research. Nature 2012;485:446.

131. Freedman SB, Powell E, Seshadri R. Predictors of Outcomes in Pediatric Enteritis: A Prospective Cohort Study. Pediatrics 2009;123:e9-e16.

132. Freedman SB, Tung C, Cho D, Rumantir M, Chan KJ. Time-series analysis of ondansetron use in pediatric gastroenteritis. J Pediatr Gastroenterol Nutr 2012;54:381-6.

133. National Center for Complementary and Alternative Medicine. (2008). An introduction to probiotics. Available: http://nccam.nih.gov/health/probiotics/. Last accessed January 2, 2009.

134. Hanson LA, Korotkova M, Lundin S, et al. The transfer of immunity from mother to child. Annals of the New York Academy of Sciences 2003;987:199-206.

135. Walker WA. Role of nutrients and bacterial colonization in the development of intestinal host defense. J Pediatr Gastroenterol Nutr 2000;30 Suppl 2:S2-7.

136. Mazanec MB, Nedrud JG, Kaetzel CS, Lamm ME. A three-tiered view of the role of IgA in mucosal defense. Immunol Today 1993;14:430-5.

137. Bouvet JP, Fischetti VA. Diversity of antibody-mediated immunity at the mucosal barrier. Infect Immun 1999;67:2687-91.

138. Guarino A, Lo Vecchio A, Canani RB. Probiotics as prevention and treatment for diarrhea. Curr Opin Gastroenterol 2009;25:18-23.

139. Lebeer S, Vanderleyden J, De Keersmaecker SC. Genes and molecules of lactobacilli supporting probiotic action. Microbiol Mol Biol Rev 2008;72:728-64.

140. Suzuki K, Meek B, Doi Y, et al. Aberrant expansion of segmented filamentous bacteria in IgA-deficient gut. Proc Natl Acad Sci U S A 2004;101:1981-6.

141. Chen CC, Kong MS, Lai MW, et al. Probiotics have clinical, microbiologic, and immunologic efficacy in acute infectious diarrhea. Pediatric Infectious Disease Journal 2010;29:135-8.

142. Fedorak RN, Madsen KL. Probiotics and prebiotics in gastrointestinal disorders. Curr Opin Gastroenterol 2004;20:146-55.

143. Duggan C, Gannon J, Walker WA. Protective nutrients and functional foods for the gastrointestinal tract. Am J Clin Nutr 2002;75:789-808.

144. Kabeerdoss J, Devi RS, Mary RR, et al. Effect of yoghurt containing Bifidobacterium lactis Bb12(R) on faecal excretion of secretory immunoglobulin A and human beta-defensin 2 in healthy adult volunteers. Nutr J 2011;10:138.

145. Fukushima Y, Kawata Y, Hara H, Terada A, Mitsuoka T. Effect of a probiotic formula on intestinal immunoglobulin A production in healthy children. Int J Food Microbiol 1998;42:39-44.

146. Mullie C, Yazourh A, Thibault H, et al. Increased poliovirus-specific intestinal antibody response coincides with promotion of Bifidobacterium longum-infantis and Bifidobacterium breve in infants: a randomized, double-blind, placebo-controlled trial. Pediatr Res 2004;56:791-5.

147. Osmond MH, Klassen TP, Wells GA, et al. CATCH: a clinical decision rule for the use of computed tomography in children with minor head injury. CMAJ 2010;182:341-8.

148. Campeotto F, Suau A, Kapel N, et al. A fermented formula in pre-term infants: clinical tolerance, gut microbiota, down-regulation of faecal calprotectin and up-regulation of faecal secretory IgA. Br J Nutr 2011:1-10.
149. Phillips G, Tam CC, Conti S, et al. Community incidence of norovirus-associated infectious intestinal disease in England: improved estimates using viral load for norovirus diagnosis. Am J Epidemiol 2010;171:1014-22.
150. Barreira DM, Ferreira MS, Fumian TM, et al. Viral load and genotypes of noroviruses in symptomatic and asymptomatic children in Southeastern Brazil. J Clin Virol 2010;47:60-4.
151. Partridge DG, Evans CM, Raza M, Kudesia G, Parsons HK. Lessons from a large norovirus outbreak: impact of viral load, patient age and ward design on duration of symptoms and shedding and likelihood of transmission. J Hosp Infect 2012;81:25-30.
152. Furuya D, Kuribayashi K, Hosono Y, et al. Age, viral copy number, and immunosuppressive therapy affect the duration of norovirus RNA excretion in inpatients diagnosed with norovirus infection. Jpn J Infect Dis 2011;64:104-8.
153. Aoki Y, Suto A, Mizuta K, Ahiko T, Osaka K, Matsuzaki Y. Duration of norovirus excretion and the longitudinal course of viral load in norovirus-infected elderly patients. J Hosp Infect 2010;75:42-6.
154. Phillips G, Lopman B, Tam CC, Iturriza-Gomara M, Brown D, Gray J. Diagnosing norovirus-associated infectious intestinal disease using viral load. BMC Infect Dis 2009;9:63.
155. Fumian TM, Justino MC, Mascarenhas JD, et al. Quantitative and molecular analysis of noroviruses RNA in blood from children hospitalized for acute gastroenteritis in Belem, Brazil. J Clin Virol 2013;58:31-5.
156. Takanashi S, Hashira S, Matsunaga T, et al. Detection, genetic characterization, and quantification of norovirus RNA from sera of children with gastroenteritis. J Clin Virol 2009;44:161-3.
157. Kang G, Iturriza-Gomara M, Wheeler JG, et al. Quantitation of group A rotavirus by real-time reverse-transcription-polymerase chain reaction: correlation with clinical severity in children in South India. J Med Virol 2004;73:118-22.
158. Deschaght P, Schelstraete P, Van Simaey L, et al. Is the Improvement of CF Patients, Hospitalized for Pulmonary Exacerbation, Correlated to a Decrease in Bacterial Load? PLoS One 2013;8:e79010.
159. Darton T, Guiver M, Naylor S, et al. Severity of meningococcal disease associated with genomic bacterial load. Clin Infect Dis 2009;48:587-94.
160. Valentine ME, Jackson CR, Vavro C, et al. Evaluation of surrogate markers and clinical outcomes in two-year follow-up of eighty-six human immunodeficiency virus-infected pediatric patients. Pediatr Infect Dis J 1998;17:18-23.
161. Liu J, Kibiki G, Maro V, et al. Multiplex reverse transcription PCR Luminex assay for detection and quantitation of viral agents of gastroenteritis. J Clin Virol 2011;50:308-13.
162. Hasing ME, Lee BE, Preiksaitis JK, et al. Emergence of a new norovirus GII.4 variant and changes in the historical biennial pattern of norovirus outbreak activity in Alberta, Canada, from 2008 to 2013. J Clin Microbiol 2013;51:2204-11.
163. Pang X, Cao M, Zhang M, Lee B. Increased sensitivity for various rotavirus genotypes in stool specimens by amending three mismatched nucleotides in the forward primer of a real-time RT-PCR assay. J Virol Methods 2011;172:85-7.
164. Pang XL, Lee BE, Pabbaraju K, et al. Pre-analytical and analytical procedures for the detection of enteric viruses and enterovirus in water samples. J Virol Methods 2012;184:77-83.
165. Pang XL, Lee BE, Tyrrell GJ, Preiksaitis JK. Epidemiology and genotype analysis of sapovirus associated with gastroenteritis outbreaks in Alberta, Canada: 2004-2007. J Infect Dis 2009;199:547-51.
166. Pang XL, Preiksaitis JK, Lee B. Multiplex real time RT-PCR for the detection and quantitation of norovirus genogroups I and II in patients with acute gastroenteritis. J Clin Virol 2005;33:168-71.

167. Pang XL, Preiksaitis JK, Wong S, Li V, Lee BE. Influence of novel norovirus GII.4 variants on gastroenteritis outbreak dynamics in Alberta and the Northern Territories, Canada between 2000 and 2008. PLoS One 2010;5:e11599.
168. Kageyama T, Kojima S, Shinohara M, et al. Broadly reactive and highly sensitive assay for Norwalk-like viruses based on real-time quantitative reverse transcription-PCR. Journal of clinical microbiology 2003;41:1548-57.
169. Pang XL, Lee B, Boroumand N, Leblanc B, Preiksaitis JK, Yu Ip CC. Increased detection of rotavirus using a real time reverse transcription-polymerase chain reaction (RT-PCR) assay in stool specimens from children with diarrhea. J Med Virol 2004;72:496-501.
170. Oka T, Katayama K, Hansman GS, et al. Detection of human sapovirus by real-time reverse transcription-polymerase chain reaction. Journal of medical virology 2006;78:1347-53.
171. Iturriza-Gomara M, Kang G, Gray J. Rotavirus genotyping: keeping up with an evolving population of human rotaviruses. J Clin Virol 2004;31:259-65.
172. Willan AR, Kowgier ME. Cost-effectiveness analysis of a multinational RCT with a binary measure of effectiveness and an interacting covariate. Health Econ 2008;17:777-91.
173. Manca A, Willan AR. 'Lost in translation': accounting for between-country differences in the analysis of multinational cost-effectiveness data. Pharmacoeconomics 2006;24:1101-19.
174. Willan AR, Goeree R, Pullenayegum EM, McBurney C, Blackhouse G. Economic evaluation of rivastigmine in patients with Parkinson's disease dementia. Pharmacoeconomics 2006;24:93-106.
175. Reed SD, Anstrom KJ, Bakhai A, et al. Conducting economic evaluations alongside multinational clinical trials: toward a research consensus. Am Heart J 2005;149:434-43.
176. Pinto EM, Willan AR, O'Brien BJ. Cost-effectiveness analysis for multinational clinical trials. Stat Med 2005;24:1965-82.
177. Willan AR, Pinto EM, O'Brien BJ, et al. Country specific cost comparisons from multinational clinical trials using empirical Bayesian shrinkage estimation: the Canadian ASSENT-3 economic analysis. Health Econ 2005;14:327-38.
178. Fontana M, Zuin G, Pancheri P, Fusco FC, Lambertini A, Berni Canani R. Costs associated with outpatient diarrhoea in infants and toddlers: a nationwide study of the Italian Society of Paediatric Gastroenterology and Hepatology (SIGEP). Dig Liver Dis 2004;36:523-7.
179. Le Saux N, Bettinger J, Dery P, et al. The hidden costs and characteristics of childhood rotavirus emergency visits in Canada. Pediatr Infect Dis J 2012;31:159-63.
180. Fleiss J. Statistical methods for rates and proportions, 2nd ed. New York: John Wiley & Sons; 1981.
181. Corneli HM, Zorc JJ, Mahajan P, et al. A multicenter, randomized, controlled trial of dexamethasone for bronchiolitis. The New England journal of medicine 2007;357:331-9.
182. Scholtens PA, Alliet P, Raes M, et al. Fecal secretory immunoglobulin A is increased in healthy infants who receive a formula with short-chain galacto-oligosaccharides and long-chain fructo-oligosaccharides. J Nutr 2008;138:1141-7.
183. Denno DM, Shaikh N, Stapp JR, et al. Diarrhea etiology in a pediatric emergency department: a case control study. Clin Infect Dis 2012;55:897-904.
184. Denno DM, Stapp JR, Boster DR, et al. Etiology of diarrhea in pediatric outpatient settings. Pediatr Infect Dis J 2005;24:142-8.
185. Shavit I, Brant R, Nijssen-Jordan C, Galbraith R, Johnson DW. A novel imaging technique to measure capillary-refill time: improving diagnostic accuracy for dehydration in young children with gastroenteritis. Pediatrics 2006;118:2402-8.
186. Johnson DW, Jacobson S, Edney PC, Hadfield P, Mundy ME, Schuh S. A comparison of nebulized budesonide, intramuscular dexamethasone, and placebo for moderately severe croup. N Engl J Med 1998;339:498-503.

187. Dendukuri N, Costa V, McGregor M, Brophy JM. Probiotic therapy for the prevention and treatment of Clostridium difficile-associated diarrhea: a systematic review. CMAJ 2005;173:167-70.
188. Salazar-Lindo E, Figueroa-Quintanilla D, Caciano MI, Reto-Valiente V, Chauviere G, Colin P. Effectiveness and safety of Lactobacillus LB in the treatment of mild acute diarrhea in children. J Pediatr Gastroenterol Nutr 2007;44:571-6.
189. Gravel J, Fitzpatrick E, Gouin S, et al. Performance of the Canadian Triage and Acuity Scale for Children: A Multicenter Database Study. Ann Emerg Med 2012.
190. Gravel J, Gouin S, Goldman RD, et al. The canadian triage and acuity scale for children: a prospective multicenter evaluation. Ann Emerg Med 2012;60:71-7 e3.
191. Plint AC, Johnson DW, Wiebe N, et al. Practice variation among pediatric emergency departments in the treatment of bronchiolitis. Acad Emerg Med 2004;11:353-60.
192. Erickson LJ, De Wals P, Farand L. An analytical framework for immunization programs in Canada. Vaccine 2005;23:2470-6.
193. Vega RM, Avner JR. A prospective study of the usefulness of clinical and laboratory parameters for predicting percentage of dehydration in children. Pediatr Emerg Care 1997;13:179-82.
194. Freedman SB, Cho D, Boutis K, Stephens D, Schuh S. Assessing the palatability of oral rehydration solutions in school-aged children: a randomized crossover trial. Arch Pediatr Adolesc Med 2010;164:696-702.
195. Freedman SB, Deiratany S, Goldman RD, Benseler S. Development of a Caregiver Gastroenteritis Knowledge Questionnaire. Ambul Pediatr 2008;8:261-5.
196. Kinlin LM, Freedman SB. Evaluation of a clinical dehydration scale in children requiring intravenous rehydration. Pediatrics 2012;129:e1211-9.
197. Freedman SB. Acute infectious pediatric gastroenteritis: beyond oral rehydration therapy. Expert Opin Pharmacother 2007;8:1651-65.
198. Roslund G, Hepps TS, McQuillen KK. The role of oral ondansetron in children with vomiting as a result of acute gastritis/gastroenteritis who have failed oral rehydration therapy: a randomized controlled trial. Ann Emerg Med 2008;52:22-9 e6.
199. DeCamp LR, Byerley JS, Doshi N, Steiner MJ. Use of antiemetic agents in acute gastroenteritis: a systematic review and meta-analysis. Arch Pediatr Adolesc Med 2008;162:858-65.
200. Ramsook C, Sahagun-Carreon I, Kozinetz CA, Moro-Sutherland D. A randomized clinical trial comparing oral ondansetron with placebo in children with vomiting from acute gastroenteritis. Ann Emerg Med 2002;39:397-403.
201. Gorelick MH. Bias arising from missing data in predictive models. Journal of Clinical Epidemiology 2006;59:1115-23.
202. Gorelick MH, Wagner D, McLellan SL. Development and validation of a self-administered questionnaire to measure water exposures in children. Ambul Pediatr 2008;8:388-91.
203. Gorelick MH, Meurer JR, Walsh-Kelly CM, et al. Emergency department allies: a controlled trial of two emergency department-based follow-up interventions to improve asthma outcomes in children. Pediatrics 2006;117:S127-34.
204. Gorelick MH, Yen K. The kappa statistic was representative of empirically observed inter-rater agreement for physical findings. Journal of Clinical Epidemiology 2006;59:859-61.
205. Majowicz SE, McNab WB, Sockett P, et al. Burden and cost of gastroenteritis in a Canadian community. J Food Prot 2006;69:651-9.
206. Alessandrini EA, Alpern ER, Chamberlain JM, Shea JA, Gorelick MH. A new diagnosis grouping system for child emergency department visits. Acad Emerg Med 2010;17:204-13.
207. Gorelick MH, Atabaki SM, Hoyle J, et al. Interobserver agreement in assessment of clinical variables in children with blunt head trauma. Acad Emerg Med 2008;15:812-8.
208. Liu L, Johnson HL, Cousens S, et al. Global, regional, and national causes of child mortality: an updated systematic analysis for 2010 with time trends since 2000. Lancet 2012;379:2151-61.

209. Alessandrini EA, Alpern ER, Chamberlain JM, Shea JA, Holubkov R, Gorelick MH. Developing a diagnosis-based severity classification system for use in emergency medical services for children. Acad Emerg Med 2012;19:70-8.
210. Scott S, Hartling L, Grimshaw J, et al. Improving outcomes for ill and injured children in emergency departments: protocol for a program in pediatric emergency medicine and knowledge translation science. Implement Sci 2009;4:60.
211. Dayan PS, Osmond M, Kuppermann N, et al. Development of the capacity necessary to perform and promote knowledge translation research in emergency medicine. Acad Emerg Med 2007;14:978-83.
212. Hartling L, Scott-Findlay S, Johnson D, et al. Bridging the gap between clinical research and knowledge translation in pediatric emergency medicine. Acad Emerg Med 2007;14:968-77.
213. Johnson-Henry KC, Hagen KE, Gordonpour M, Tompkins TA, Sherman PM. Surface-layer protein extracts from Lactobacillus helveticus inhibit enterohaemorrhagic Escherichia coli O157:H7 adhesion to epithelial cells. Cell Microbiol 2007;9:356-67.
214. Eckermann S, Coory M, Willan AR. Indirect comparison: relative risk fallacies and odds solution. J Clin Epidemiol 2009;62:1031-6.
215. Eckermann S, Willan AR. Globally optimal trial design for local decision making. Health Econ 2009;18:203-16.
216. Willan AR, Eckermann S. Optimal clinical trial design using value of information methods with imperfect implementation. Health Econ 2009.
217. Emery CA, Kang J, Shrier I, et al. Risk of injury associated with body checking among youth ice hockey players. JAMA 2010;303:2265-72.
218. Hansen G, Joffe AR, Nettel-Aguirre A, et al. Two-year survival and neurodevelopmental outcomes after cardiopulmonary resuscitation in neonatal patients after complex cardiac surgery. Resuscitation 2011;82:313-8.
219. Samuel SM, Foster BJ, Hemmelgarn BR, et al. Incidence and causes of end-stage renal disease among Aboriginal children and young adults. CMAJ 2012;184:E758-64.
220. Emery C, Kang J, Shrier I, et al. Risk of injury associated with bodychecking experience among youth hockey players. CMAJ 2011;183:1249-56.
221. Samuel SM, Foster BJ, Tonelli MA, et al. Dialysis and transplantation among Aboriginal children with kidney failure. CMAJ 2011;183:E665-72.
222. Demirjian A, Finkelstein Y, Nava-Ocampo A, et al. A Randomized Controlled Trial of a Vancomycin Loading Dose in Children. Pediatr Infect Dis J 2013.
223. Huang YF, Liu PY, Chen YY, et al. Three-combination probiotics therapy in children with salmonella and rotavirus gastroenteritis. J Clin Gastroenterol 2014;48:37-42.
224. Freedman SB, Uleryk E, Rumantir M, Finkelstein Y. Ondansetron and the Risk of Cardiac Arrhythmias: A Systematic Review and Postmarketing Analysis. Ann Emerg Med 2013.
225. Finkelstein Y, Moran O, Avitzur Y, et al. Clinical dysentery in hospitalized children. Infection 2002;30:132-5.
226. Hoffer V, Finkelstein Y, Balter J, Feinmesser M, Garty BZ. Ganciclovir treatment in Menetrier's disease. Acta Paediatr 2003;92:983-5.
227. Finkelstein Y, Schechter T, Freedman SB. Surgical masks vs N95 respirators for preventing influenza. JAMA 2010;303:938; author reply -9.
228. Finkelstein Y, Hutson JR, Freedman SB, Wax P, Brent J, Toxicology Investigators Consortium Case R. Drug-induced seizures in children and adolescents presenting for emergency care: current and emerging trends. Clin Toxicol (Phila) 2013;51:761-6.
229. Freedman SB, Powell EC, Nava-Ocampo AA, Finkelstein Y. Ondansetron dosing in pediatric gastroenteritis: a prospective cohort, dose-response study. Paediatr Drugs 2010;12:405-10.
230. Lee BE, Pang XL, Robinson JL, Bigam D, Monroe SS, Preiksaitis JK. Chronic norovirus and adenovirus infection in a solid organ transplant recipient. Pediatr Infect Dis J 2008;27:360-2.

231. Mattison K, Grudeski E, Auk B, et al. Analytical performance of norovirus real-time RT-PCR detection protocols in Canadian laboratories. J Clin Virol 2011;50:109-13.
232. Pang X, Lee B, Chui L, Preiksaitis JK, Monroe SS. Evaluation and validation of real-time reverse transcription-pcr assay using the LightCycler system for detection and quantitation of norovirus. J Clin Microbiol 2004;42:4679-85.
233. Pang XL, Fox JD, Fenton JM, et al. Interlaboratory comparison of cytomegalovirus viral load assays. Am J Transplant 2009;9:258-68.
234. Preiksaitis JK, Pang XL, Fox JD, et al. Interlaboratory comparison of epstein-barr virus viral load assays. Am J Transplant 2009;9:269-79.
235. Chui L, Lee MC, Allen R, Bryks A, Haines L, Boras V. Comparison between ImmunoCard STAT!((R)) and real-time PCR as screening tools for both O157:H7 and non-O157 Shiga toxin-producing Escherichia coli in Southern Alberta, Canada. Diagn Microbiol Infect Dis 2013;77:8-13.
236. Chui L, Lee MC, Malejczyk K, Lim L, Fok D, Kwong P. Prevalence of shiga toxin-producing Escherichia coli as detected by enzyme-linked immunoassays and real-time PCR during the summer months in northern Alberta, Canada. J Clin Microbiol 2011;49:4307-10.
237. Couturier MR, Lee B, Zelyas N, Chui L. Shiga-toxigenic Escherichia coli detection in stool samples screened for viral gastroenteritis in Alberta, Canada. J Clin Microbiol 2011;49:574-8.
238. Chui L, Couturier MR, Chiu T, et al. Comparison of Shiga toxin-producing Escherichia coli detection methods using clinical stool samples. J Mol Diagn 2010;12:469-75.
239. Gilmour MW, Olson AB, Andrysiak AK, Ng LK, Chui L. Sequence-based typing of genetic targets encoded outside of the O-antigen gene cluster is indicative of Shiga toxin-producing Escherichia coli serogroup lineages. J Med Microbiol 2007;56:620-8.
240. Gilmour MW, Chui L, Chiu T, et al. Isolation and detection of Shiga toxin-producing Escherichia coli in clinical stool samples using conventional and molecular methods. J Med Microbiol 2009;58:905-11.
241. Chui L, Chiu T, Kakulphimp J, Tyrrell GJ. A comparison of three real-time PCR assays for the confirmation of Neisseria gonorrhoeae following detection of N. gonorrhoeae using Roche COBAS AMPLICOR. Clin Microbiol Infect 2008;14:473-9.
242. Louie M, Read S, Simor AE, et al. Application of multiplex PCR for detection of non-O157 verocytotoxin-producing Escherichia coli in bloody stools: identification of serogroups O26 and O111. J Clin Microbiol 1998;36:3375-7.
243. Pearl DL, Louie M, Chui L, et al. A multi-level approach for investigating socio-economic and agricultural risk factors associated with rates of reported cases of Escherichia coli O157 in humans in Alberta, Canada. Zoonoses Public Health 2009;56:455-64.
244. Krief WI, Levine DA, Platt SL, et al. Influenza virus infection and the risk of serious bacterial infections in young febrile infants. Pediatrics 2009;124:30-9.
245. Kanegaye JT, Nigrovic LE, Malley R, et al. Diagnostic value of immature neutrophils (bands) in the cerebrospinal fluid of children with cerebrospinal fluid pleocytosis. Pediatrics 2009;123:e967-71.
246. Glaser N, Barnett P, McCaslin I, et al. Risk factors for cerebral edema in children with diabetic ketoacidosis. The Pediatric Emergency Medicine Collaborative Research Committee of the American Academy of Pediatrics. The New England journal of medicine 2001;344:264-9.
247. Kuppermann N, Holmes JF, Dayan PS, et al. Identification of children at very low risk of clinically-important brain injuries after head trauma: a prospective cohort study. Lancet 2009;374:1160-70.
248. Nigrovic LE, Malley R, Macias CG, et al. Effect of antibiotic pretreatment on cerebrospinal fluid profiles of children with bacterial meningitis. Pediatrics 2008;122:726-30.
249. Moler FW, Meert K, Donaldson AE, et al. In-hospital versus out-of-hospital pediatric cardiac arrest: a multicenter cohort study. Crit Care Med 2009;37:2259-67.

250. Meert KL, Donaldson A, Nadkarni V, et al. Multicenter cohort study of in-hospital pediatric cardiac arrest. Pediatr Crit Care Med 2009;10:544-53.

251. Carcillo J, Holubkov R, Dean JM, et al. Rationale and design of the pediatric critical illness stress-induced immune suppression (CRISIS) prevention trial. JPEN J Parenter Enteral Nutr 2009;33:368-74.

252. Holubkov R, Dean JM, Berger J, et al. Is "rescue" therapy ethical in randomized controlled trials? Pediatr Crit Care Med 2009;10:431-8.

253. Guthery SL, Dong L, Dean JM, Holubkov R. US estimates of hospitalized pediatric patients with ulcerative colitis: implications for multicenter clinical studies. Inflamm Bowel Dis 2008;14:1253-8.
