# Supplementary material for: Variables Associated With Intravenous Rehydration and Hospitalization in Children With Acute Gastroenteritis: A Secondary Analysis of 2 Randomized Clinical Trials
Source: JAMA Netw Open. 2021 Apr 19;4(4):e216433. doi: 10.1001/jamanetworkopen.2021.6433 (PMC8056281; doi:10.1001/jamanetworkopen.2021.6433)
Supplement: Supplement 2. — PECARN Protocol [file jamanetwopen-e216433-s002.pdf]

Impact of Emergency Department Probiotic Treatment of Pediatric Gastroenteritis (Probiotics Study)
PECARN Protocol Number 032

Pediatric Emergency Care Applied Research Network
National Institute for Child Health and Human Development (NICHD)

Protocol Version 3.00
Version Date: February 13, 2017
Printing Date: February 13, 2017

Copyright c 2014. University of Utah School of Medicine on behalf of the Principal Investigator, David Schnadower, M.D. and the Pediatric Emergency Care Applied Research Network (PECARN). All rights reserved.

This protocol is PECARN Protocol Number 032, and has been authored by David Schnadower, M.D., Washington University, for implementation with the PECARN investigators. This study is supported by R01-HD071915 awarded to Washington University (PI:David Schnadower, M.D.) by the Eunice Kennedy Shriver National Institute for Child Health and Human Development (NICHD).

The PECARN research node centers are Washington University, University of Michigan at Ann Arbor, Cincinnati Children's Hospital Medical Center, Columbia University, University of California at Davis Medical Center, Children's Hospital of Pittsburgh, and Children's National Medical Center and are supported by Cooperative Agreements U03-MC00003, U03-MC22684, U03-MC00007, U03-MC00001, U03MC22685, and U03-MC00006 from the Emergency Medical Services for Children (EMSC) Program, Maternal and Child Health Bureau, Health Resources and Services Administration.

This document was prepared by the PECARN EMSC Data Coordinating Center (DCC) located at the University of Utah School of Medicine, Salt Lake City, Utah. The document was written and typeset using LaTeX 2ε. The DCC at the University of Utah is supported by Cooperative Agreement U03-MC00008 from the Emergency Medical Services for Children (EMSC) Program, Maternal and Child Health Bureau, Health Resources and Services Administration.

Protocol 032 (Schnadower) Page 3 of 49

PROTOCOL TITLE:

Impact of Emergency Department Probiotic Treatment of Pediatric Gastroenteritis

Short Title: Probiotics Study
PECARN Protocol Number: 032

Lead Investigator and Author:
David Schnadower, M.D.
Washington University

Protocol Version: 3.00
Version Date: February 13, 2017

I confirm that I have read this protocol, I understand it, and I will conduct the study according to the protocol. I will also work consistently with the ethical principles that have their origin in the Declaration of Helsinki and will adhere to the Ethical and Regulatory Considerations as stated. I confirm that if I or any of my staff are members of the Institutional Review Board, we will abstain from voting on this protocol, its future renewals, and its future amendments.

Principal Investigator Name: ______________________________

Principal Investigator Signature: ______________________________

Date: ______________________________

Protocol 032 (Schnadower) Page 4 of 49

THIS PAGE IS INTENTIONALLY BLANK.

Contents

1 Study Summary 9
1.1 Hypotheses . . . 9
1.2 Specific Aims . . . 9
1.3 Trial Endpoints . . . 10
1.4 Subject Eligibility, Accrual and Study Duration . . . 10

2 Rationale and Background 11
2.1 Rationale for Probiotic E cacy . . . 12
2.2 Limitations of Research to Date . . . 13
2.3 Validation of the Modified Vesikari Score . . . 14

3 Study Design and Data Collection 15
3.1 Study Design Overview . . . 15
3.2 Participant Screening and Consent . . . 15
3.3 Baseline Data Collection . . . 16
3.4 Follow Up Data Collection . . . 16

4 Study Procedures 17
4.1 Randomization (Enrollment) . . . 17
4.2 Study Drug Administration . . . 17
4.3 Discontinuation of Study Drug . . . 19
4.4 Withdrawal from Study . . . 20
4.5 Stool Sample Testing . . . 20

5 Data Analysis 21
5.1 Specific Aim Analyses . . . 21
5.2 Secondary Study Outcomes . . . 23
5.3 Sample Size Calculations and Statistical Power . . . 24
5.3.1 Enrichment Design . . . 25
5.3.2 Sub-group Analyses . . . 26
5.4 Recruitment Estimates and Attrition . . . 26
5.4.1 Recruitment . . . 26
5.4.2 Potential for Bias . . . 26
5.4.3 Compliance . . . 27
5.4.4 Loss to Follow-up . . . 27
5.4.5 Compensation . . . 27

6 Data Management 27
6.1 Clinical Sites . . . 27
6.2 PECARN Data Coordinating Center (Utah) . . . 28
6.3 Data Confidentiality . . . 29
6.4 Data Quality Management and Monitoring . . . 29
6.5 Record Access . . . 30

7 Protection of Human Subjects 30
7.1 Institutional Review Board (IRB) Approval . . . 30
7.2 Informed Consent . . . 30
7.3 Potential Risks . . . 30
7.4 Protections Against Potential Risks . . . 31
7.5 Potential Benefits . . . 31

8 Data and Safety Monitoring Plan 32
8.1 Data Safety Monitoring Board (DSMB) . . . 32
8.2 Frequency of Interim Analysis: . . . 33
8.3 Adverse Event Reporting . . . 33
8.3.1 Definitions, Relatedness, Severity and Expectedness . . . 33
8.3.2 Time Period for Adverse Events . . . 35
8.3.3 Data Collection Procedures for Adverse Events . . . 35
8.3.4 Unanticipated Problems (UP) . . . 36
8.3.5 Monitoring Serious Adverse Events . . . 36
8.3.6 Follow-up of Serious, Unexpected and Related Adverse Events . . . 37

9 Study Training and Monitoring 37
9.1 Study Training . . . 37
9.2 Study Monitoring . . . 38
9.2.1 Site Monitoring Plan . . . 38
9.2.2 Clinical Site Monitoring . . . 38
9.2.3 Remote Monitoring . . . 39
9.2.4 Pharmacy Monitoring . . . 39

10 Regulatory Issues 39
10.1 Food and Drug Administration . . . 39
10.2 Health Insurance Portability and Accountability Act . . . 39
10.3 Inclusion of Women and Minorities . . . 40
10.4 ClinicalTrials.gov Requirements . . . 40
10.5 Retention of Records . . . 40

Bibliography 40

List of Tables

1 Modified Vesikari Score 15
2 Power Analysis 25

Abstract

Practice guidelines do not endorse routine use of probiotics in pediatric acute gastroenteritis. However, probiotic manufacturers aggressively purvey probiotics using health claims that have not been supported by rigorous research, and the world-wide market for probiotic products is equivalent to $32.6 billion per year. The Food and Drug Administration and the European Food Safety Authority remain concerned about probiotic value and safety.[1–3] Some institutions now recommend the routine use of probiotics based on potentially flawed or limited evidence,[4] and parents of patients with acute gastroenteritis often administer probiotics to their children without guidance from medical professionals.[5] Since the use of probiotics is increasing without adequate evidence to support its use in the United States, the study described in this protocol was designed and successfully submitted to the National Institute for Child Health and Human Development (NICHD) for funding.

The overall objective of this randomized, placebo-controlled, double-blind study is to determine if probiotic administration reduces the severity of acute gastroenteritis episodes in children aged 3 to 48 months. The probiotic agent that will be used in this trial is Lactobacillus rhamnosus GG, ATCC 53103 (LGG).

Participants will be randomized to receive a five-day course of probiotic or placebo. The trial is anticipated to enroll 970 children, age 3-48 months, treated in participating emergency departments for acute gastroenteritis. We define acute gastroenteritis as the presence of three or more watery stools in a 24-hour period. Children with underlying illness that pose potential increased risk from probiotic therapy (e.g. indwelling vascular access line, structural heart disease, immunosuppressive therapy or known immunodeficiency, chronic gastrointestinal disease such as inflammatory bowel disease, or household contact with immunodeficiency or immunosuppressive therapy) will be excluded. The age range of 3-48 months was selected because this age group has the highest incidence of acute gastroenteritis and the greatest morbidity. Subjects will be recruited at the time of an emergency department visit. All eligible subjects who present for treatment when study personnel are available will be approached for participation. Those agreeing to participate will be randomized, using block randomization stratified by study site, to treatment groups. The study will be conducted at multiple sites within the Pediatric Emergency Care Applied Research Network (PECARN). We will also collect and freeze pre- and post-treatment bulk stool specimens at the lead study

Protocol 032 (Schnadower) Page 9 of 49

site to assess the mechanism of action of the specific probiotic agent, Lactobacillus rhamnosus GG, ATCC 53103 (LGG).

Probiotics Study Protocol Version 3.00
Protocol Version Date: February 13, 2017
The EMSC Pediatric Emergency Care Applied Research Network

1 Study Summary

This randomized, double-blind, placebo-controlled trial will quantify the benefits and potential side effects associated with probiotic administration in ambulatory children presenting to the emergency department with acute gastroenteritis. This will provide the first definitive evidence in the United States for, or against, using probiotic therapy for this condition. This study will use the most commonly employed probiotic agent (Lactobacillus rhamnosus GG, ATCC 53103 (LGG)) approved for use in the United States. The results of this multicenter study will guide the standard of care: if probiotic administration is associated with benefit, it offers an inexpensive, safe and easy way to administer treatment to reduce morbidity from acute gastroenteritis. If the trial does not demonstrate probiotic efficacy, healthcare, caregiver and societal resources may be refocused on alternative therapeutic interventions.

1.1 Hypotheses

The hypotheses of this study are:

1. In children with acute gastroenteritis, probiotic administration in the emergency department will be associated with a clinically-important decrease in the proportion of children suffering from moderate-severe disease, defined by a validated Modified Vesikari Score [illegible] 9, compared to placebo.

2. In children with acute gastroenteritis, probiotic administration will not be associated with serious adverse events, and will have a similar rate of side effects (e.g. bloating, fever) as compared to placebo-treated children.

1.2 Specific Aims

This project has the following Specific Aims:

Specific Aim 1. Determine the clinical effectiveness of probiotic administration to reduce morbidity in children presenting to the emergency department with acute gastroenteritis.

Protocol 032 (Schnadower) Page 11 of 49

Specific Aim 2. Determine the safety and side effect profiles of Lactobacillus rhamnosus GG, ATCC 53103 (LGG) in children presenting to the emergency department with acute gastroenteritis.

1.3 Trial Endpoints

Primary Endpoints

- The primary efficacy endpoint of this study is the presence of moderate-severe acute gastroenteritis, as defined by a total post-enrollment Modified Vesikari Score 9 during the 2-week follow-up period.

- The primary safety endpoint of this study is the occurrence of invasive disease (including meningitis and bacteremia) from Lactobacillus rhamnosus.

Secondary Endpoints

1. Diarrhea duration: Time from randomization until the appearance of the last watery stool as reported during daily surveys.

2. Vomiting duration: Time from randomization until the last vomiting episode as reported during daily surveys. Vomiting duration is only evaluated in children who vomited 3 times during the 24 hours prior to the emergency department visit.

3. Return visits: Return visits for unscheduled care to a healthcare provider related to vomiting, diarrhea, dehydration, fever, or fluid refusal, within 2 weeks of the index visit. Scheduled visits (e.g. reassessment, vaccinations, unrelated issues) will not be included.

4. Missed daycare: Days of daycare missed by subjects who attend daycare.

5. Missed work: Days of work missed by caregivers who work outside of the home.

6. Household transmission rate: A household census will be obtained at the time of enrollment, and information about household contacts symptoms will be obtained during daily surveys to determine household transmission rate.

7. Side Effects: Occurrence of side effects such as weakness, bloating, gas, intestinal rumbling, diarrhea, blood in stool, abdominal pain, abdominal cramps, nausea, vomiting, loss of appetite, heartburn, constipation, skin rash, fever, nasal congestion, sore throat, cough, headache, muscle aches, chills, and diaper rash.

1.4 Subject Eligibility, Accrual and Study Duration

Eligible participants will be identified by on-site study staff. Inclusion criteria are:

Protocol 032 (Schnadower) Page 13 of 49

1. Age 3-48 months (have not yet reached their fourth birthday); AND
2. Presence of 3 or more watery stools within 24 hours of screening; AND
3. Duration of vomiting or diarrhea less than 7 days; AND
4. Symptoms consistent with acute intestinal infectious process.

We will exclude subjects with any of the following:

1. Presence of an indwelling vascular access line; OR
2. Presence of structural heart disease excluding non-pathological heart murmurs; OR
3. Receiving immunosuppressive therapy or history of immunodeficiency; OR
4. Hematochezia in the preceding 48 hours; OR
5. Chronic gastrointestinal problems (e.g. short gut syndrome, inflammatory bowel disease); OR
6. Patients with known pancreatitis; OR
7. History of abdominal surgery; OR
8. Critically ill patients; OR
9. Family member with an indwelling vascular access line, or on immunosuppressive therapy, or with a known immunodeficiency; OR
10. Bilious emesis; OR
11. Probiotic use (supplement) in the preceding 2 weeks; OR
12. Oral or intravenous steroid use in the preceding six months; OR
13. Previously enrolled in this trial; OR
14. Allergy to lactobacillus or Microcrystalline Cellulose (MCC); OR
15. Allergy to erythromycin, clindamycin, AND -lactam antibiotics (all); OR
16. Not available for daily follow-up while symptomatic; OR
17. Parent/guardian not speaking English or Spanish; OR
18. Under 6 months old AND premature (<37 weeks).

The trial is anticipated to enroll 970 subjects over four years, which will include four acute gastroenteritis seasons. An interim safety analysis will be prepared for Data Safety Monitoring Board (DSMB) review after the first 80 subjects (half of whom will be under 1 year of age) have been followed for 1 month. At the time of this review, the DSMB may alter the subsequent sample size based on overall event rates.

2 Rationale and Background

Acute gastroenteritis is a leading cause of malnutrition and death worldwide and affects millions of children in the United States each year.[6, 7] Acute gastroenteritis exerts high

impact burdens on children and their families: each episode causes on average 6-8 days of diarrhea, 2-4 days of vomiting, and 2-4 days of fever.[8–11] In two prospective studies conducted in 11 Canadian and 5 United States pediatric emergency departments, 433/683 (63%) children 3-48 months of age with acute gastroenteritis had moderate to-severe symptoms at presentation, resulting in substantial daycare and parental work absenteeism.[12, 13] The economic burden associated with such cases is considerable.[14] Unfortunately, current treatment options are limited to rehydration, symptomatic management and supportive care, prevention of severe dehydration and secondary infection control.[15–17]

Probiotic agents represent a novel approach to management of pediatric acute gastroenteritis. Probiotics are viable microbes that are generally considered to be safe and are relatively inexpensive, easily administered, and hypothesized to modulate disease processes.[18] They may also reduce symptoms (i.e. diarrhea, vomiting, fever)[19–22] and morbidity, thereby diminishing acute gastroenteritis-associated resource consumption (e.g. physician visits, hospitalization) and societal costs. Numerous studies report promising results in children with acute gastroenteritis treated with probiotics, but these studies have been inadequate to change clinical practice, because of flaws in methodology including small sample sizes, lack of probiotic quality control, and outcomes that are of minimal relevance to patients and their families.[19] Remarkably, few studies of probiotics have evaluated outpatients, a group representing >90% of acute gastroenteritis episodes in the United States.[23, 24] Moreover, only a single emergency department study of probiotic e cacy in acute gastroenteritis has been reported.[20]

To date, practice guidelines do not endorse routine use of probiotics in pediatric acute gastroenteritis.[5, 19, 25–27] However, current trends provide the motivations for the study described in this protocol. First, probiotic manufacturers aggressively purvey probiotics using health claims that have not been supported by rigorous research,[28–31] and the world-wide market for probiotic products is growing rapidly (projected growth 2009-2014 12.6%, now equivalent to $32.6 billion per year).[32] Second, the United States Food and Drug Administration and the European Food Safety Authority remain concerned about probiotic value and safety.[1–3] Third, some institutions now recommend the routine use of probiotics based on potentially flawed or limited evidence.[4] Fourth, parents of patients with acute gastroenteritis often administer probiotics to their children without guidance from medical professionals.[5]

2.1 Rationale for Probiotic E cacy

Protocol 032 (Schnadower) Page 16 of 49

Probiotics, defined as viable microbial preparations with beneficial effects on the health of the host,[33] are regarded as safe, and are generally affordable and convenient (i.e., as over-the-counter food supplements). Probiotics, and Lactobacillus rhamnosus GG, ATCC

Protocol 032 (Schnadower) Page 17 of 49

53103 (LGG) in particular, promote colonization resistance and modulate immune responses in the host, and can be directly antimicrobial. Over 100 studies have studied how probiotics can improve immune function in humans.[34] At the mucosal surface, probiotics:

- compete with pathogenic bacteria for nutrients and adhesion binding sites;[35–38]
- produce antimicrobial substances;[37, 39, 40]
- provide nutrients to colonocytes;[41]
- alter epithelial gene expression;[42] and
- reduce intestinal permeability.[43]

Additional mechanisms of action include enhancing phagocyte[44] and natural killer cell activity,[45] and increasing fecal,[46] salivary,[47] and systemic[48] IgA levels.

Preliminary probiotic studies in humans, including over 56 trials in children with gastroenteritis (17 of which used some form of Lactobacillus rhamnosus GG, ATCC 53103 (LGG) , the agent employed in this trial) suggest that probiotics may improve the course of acute gastroenteritis. Meta-analyses[19, 21, 49–51] report reduced mean duration of diarrhea (by 25 hours), reduced proportion of diarrhea lasting four days (risk ratio 0.41), and reduced stool frequency on Day 2 (mean difference 0.8).[19] This has prompted some physicians and institutions to recommend probiotics in children with acute gastroenteritis. Careful examination of the data, however, shows significant methodological flaws in these studies. For these reasons, experts and agencies have continued to recommend large, unbiased, well designed definitive trials to inform practice.[19, 26]

2.2 Limitations of Research to Date

Most trials of probiotics for acute gastroenteritis have been methodologically flawed.[19] Published studies have used small sample sizes and non-standardized treatment regimens, probiotic preparations with variable production quality and non-verified colony counts. Outcomes have been focused almost exclusively on diarrhea duration, and use inconsistent (or unreported) definitions,[19, 52] without considering the more global (and relevant) assessment of the illness (e.g. fever, vomiting, repeat emergency department visits, hospitalization). Because the outcomes sought are heterogeneous, and/or not validated, and/or of minimal importance to participants,[53] and have not addressed clinical and socioeconomic efficacy,[49, 50] the value of the existing data has been questioned.[54]

Protocol 032 (Schnadower) Page 18 of 49

Moreover, few studies address side effects and adverse events systematically.[1–3]

Although >90% of children with acute gastroenteritis are treated as outpatients,[23]

Protocol 032 (Schnadower) Page 19 of 49

hospitalized children have been the main focus of most research in this area. Extrapolation to outpatients is problematic because hospitalized children are more likely to have rotavirus infections,[55] severe disease, and to benefit from probiotics.[21, 52, 56] Emergency department patients are ideal for outpatient probiotic trials, as their acute gastroenteritis disease severity represents the entire spectrum in a normal distribution,[13] o↵ering distinct advantages over primary care populations. Emergency department patients are at risk for more severe disease, there is a greater potential for benefit because of the disease acuity and the large sample size, and disease spectrum will enable identification of subgroups most likely to benefit. Emergency departments are also a high venue of presentation for acute gastroenteritis, enabling uniform trial design and compliance compared to non-emergency department ambulatory settings.[24, 57–59] Only one emergency department study has been conducted previously, but the study was underpowered and did not explore side e↵ects.[20]

2.3 Validation of the Modified Vesikari Score

The Vesikari Score, a composite measure of seven items first described in 1990,[60] has been used in many clinical trials for treating acute gastroenteritis, particularly those evaluating rotavirus vaccines in infants, and in large international epidemiological studies.[8, 61–65] The score incorporates features of acute gastroenteritis that are important to parents, children and physicians, including

- diarrhea duration
- diarrhea frequency,
- vomiting duration,
- vomiting frequency,
- maximum daily temperature,
- medical interventions required, and
- degree of dehydration.

In outpatient studies, the degree of dehydration, measured as weight loss, is problematic because it requires in-person re-evaluations, can vary based on recent voiding, defecation, and liquid intake, and also might reflect inadequate caloric intake or catabolism during acute illness. In fact, the assessment of dehydration using signs or scales designed based on the use of post-illness weight as a gold standard has been questioned.[66] Because of these limitations, modifications to the original Vesikari score have been tested.[67, 68]

Protocol 032 (Schnadower) Page 20 of 49

The modification replaces percent dehydration with the need for an unscheduled future healthcare visit within the two week follow-up period (Table 1 on the facing page). This

Protocol 032 (Schnadower) Page 21 of 49

Points	0	1	2	3
Diarrhea duration	0	1-96 hrs	97-120 hrs	121 hours
Max # of diarrheal stools/24 hrs	0	1-3	4-5	6
Vomiting duration	0	1-24 hrs	25-48 hrs	49 hours
Max # of vomiting episodes/24 hrs	0	1	2-4	5
Max recorded fever	□ 37 C	37.1-38.4 C	38.5-38.9 C	39 C
Unscheduled healthcare visit	0	–	Primary Care	Emergency Department
Treatment	None	Rehydration	Hospital Admission	–

Table 1: Modified Vesikari Score

modification is supported by evidence that the demand for professional medical care correlates with disease severity.[67] The Modified Vesikari Score has been validated in two multicenter studies[12, 13] conducted during the planning of the current trial described in this protocol.

3 Study Design and Data Collection

3.1 Study Design Overview

This is a double-blind randomized placebo controlled trial of probiotic therapy for acute gastroenteritis. Children 3 months of age to 48 months of age who present to a participating emergency department with acute gastroenteritis will be assessed for eligibility. Children for whom parents provide permission to participate in the trial will be randomized to receive probiotic therapy (Lactobacillus rhamnosus GG, ATCC 53103 (LGG)) or placebo. The trial will be analyzed as an intention-to-treat study.

3.2 Participant Screening and Consent

Potentially eligible patients will be identified and screened for eligibility. The treating physician or other study personnel will discuss the details of the study with the patient's caregivers to explain the study and obtain parental permission.

Protocol 032 (Schnadower) Page 22 of 49

For patients who are actively screened in real time and meet all inclusion criteria, data will be recorded that include yes/no answers to each of the exclusion criteria. If a patient is ineligible at this point, no further data will be recorded, and the patient's parents will

not be approached for participation in the study. If a patient is eligible, the parents will be approached to request permission for their child to participate in the study. If parents are not approached for permission, the reason the parents were not approached will be recorded. If the parents decline permission to participate, the reason for refusal will be documented (if offered by the parent). For all eligible patients who are not randomized, study personnel will record whether the patient was prescribed probiotics. The recorded exclusion criteria, reasons for declining permission, and off-study probiotic use will enable the investigators to assess barriers to trial enrollment and identify potential biases in the study.

3.3 Baseline Data Collection

Trained study staff at each site will collect baseline demographic information (e.g. birthdate, gender, race, ethnicity) and relevant clinical variables, record the data on worksheets, and enter the data into the electronic data capture system provided by the Data Coordinating Center. Baseline clinical dehydration scale[69] and disease severity scores (Modified Vesikari Score)[13] will be assigned to enable baseline comparisons between treatment arms.

3.4 Follow Up Data Collection

Successful follow up will be maximized by obtaining multiple phone numbers for families and emergency contacts, scheduling calls and sending phone text reminders, allowing for electronic completion and centralizing all follow-up procedures at the lead institution (Washington University). Trained, experienced study staff will contact the family daily (including weekends) until both the diarrhea and vomiting have resolved and the treatment has been completed on day 5. Additional contact will be made on day 14 for outcome assessment, and months 1, 3, 6, 9 and 12 for long term safety outcomes. Study staff will use a standardized data collection form, and will inquire about ongoing symptoms, medical evaluations, treatments, child care, work absenteeism, and side effects. Detailed questioning will follow positive responses. A study diary will be provided to caregivers to use as a note-taking tool. Caregivers may also be provided an option of completing the follow-up collection electronically. Patient compliance with study drug administration will be assessed on day 5. To maximize compliance, caregivers will be reminded of the importance and method of administering the study drug. On day 14, the study site staff (not the central staff conducting the follow up) will perform a chart review for final data

Protocol 032 (Schnadower) Page 24 of 49

collection and identification of recorded adverse events during the 14 day study period. Adverse event reporting is described in detail in Section 8.3 on page 33.

Every effort will be made complete daily follow-up on the day necessary. However, if one or two days of follow-up contact is missed, data for those missed days may be collected on the next contact, if the caregiver used the home diaries or can recall the information. If all contact is missed but then the parent is contacted on day 14, follow-up data for the daily calls will be collected only if the parent diaries were used. For day 5 and 14, contact may be made within + 3 days. For month 1, 3, 6, 9, and 12 follow-up, contact may be made within ± 2 weeks.

4 Study Procedures

4.1 Randomization (Enrollment)

Randomization will be accomplished through the use of an online randomization service. Subjects will be randomized to receive either probiotic (Lactobacillus rhamnosus GG, ATCC 53103 (LGG)) or placebo. Equal allocation randomization tables will be provided by the Data Coordinating Center to the central research pharmacy. The central research pharmacy will prepare consecutively numbered study kits according to the randomization schedule. Study kits will be sent to the clinical sites. Randomization tables will be created at the Data Coordinating Center using permuted-block randomization stratified by clinical site and duration of symptoms. This will ensure that variations (e.g. site specific practice patterns, gastrointestinal patterns) are comparably distributed across treatment arms. The randomization number will be recorded in the database.

4.2 Study Drug Administration

Study Drug Description

Lactobacillus rhamnosus GG, ATCC 53103 (LGG) is supplied in a gelatin capsule containing 10^{10} colony forming units of Lactobacillus rhamnosus (75 mg). The capsule also contains 250 mg of microcrystalline cellulose (MCC – purified partially depolymerized cellulose), an inert ingredient. Placebo capsules contain only MCC (to a total of 325 mg). Each capsule is wrapped in double foil to protect it against harmful light, air, and

Protocol 032 (Schnadower) Page 26 of 49

moisture. Blister packs are labeled with the lot number. The probiotic and placebo capsules have active Drug Master Files at the Food and Drug Administration (BB-MF 2 #13668 and MF2 # 13646).

Initial Study Drug Administration

Clinical staff in the emergency department will administer the first dose of study drug by sprinkling the capsule's contents into 20-30 mL of room temperature non-carbonated liquid. Capsule contents may also be sprinkled into 1 tablespoon of applesauce as an alternative to liquid. Oral fluid therapy will be encouraged according to sites' existing clinical guidelines.[26] At discharge, parents will also be provided with clinical instructions concerning what and how much fluid to drink, criteria for returning to the emergency department or seeing their physician, and other standardized discharge instructions for acute gastroenteritis. Study research coordinators will provide the parents with instructions concerning study drug administration at home, completion of study forms, and a letter for their pediatrician that explains the study. The importance of administering all doses dispensed and the need to communicate with the study team on a daily basis until symptoms resolve will be stressed.

Home Study Drug Administration

All patients will consume one capsule twice a day for 5 days (total of 9 home doses). Patients will receive the medication mixed with 20-30 mL of a room temperature non-carbonated liquid (or 1 tablespoon of applesauce as an alternative to liquid) and ingested immediately to optimize probiotic viability. One extra dose/day will be provided (i.e. kits will contain 5 extra doses total of 15 capsules) to account for vomiting or wastage. The dose may be repeated once if vomiting occurs within 15 minutes of administration; this rarely happens more than once.[70] Oral fluid therapy will be encouraged according to established guidelines.[26]

Hospital Study Drug Administration

We estimate that only a small proportion (<5%) of enrolled subjects will be hospitalized. If that does occur, subjects will continue on the study protocol as described for home study drug administration, as done in previous studies.[71] Future unscheduled hospitalization at a non-study hospital site is even less common: none of the 274 patients enrolled in our pilot study were admitted to a different institution after evaluation.[12] To minimize the impact that such an event could have on this trial, caregivers will be provided with a letter that they will be instructed to share with their pediatrician and with the admitting

Protocol 032 (Schnadower) Page 28 of 49

physician. The letter will describe the study, the care-plan, and it will include site investigator contact information and the importance of adhering to the study protocol.

 Protocol 032 (Schnadower) Page 29 of 49

4.3 Discontinuation of Study Drug

Study drugs are sometimes discontinued because of intolerance or perception of a clinical adverse event from study drug administration. If any clinical abnormality, laboratory abnormality, intercurrent illness, new immunosuppressive therapy, or other condition occurs such that continued administration of study drug would not be in the best interest of the subject in the opinion of the investigator, study drug administration may be discontinued. When this occurs, regardless of the reason, the subject remains in the intention-to-treat population, all data collection should proceed, and long term follow up for adverse events is carried out. Discontinuation of study drug will be documented and will be reported in the closed session of the next scheduled Data Safety Monitoring Board meeting. Clinical staff and parents should not report the early discontinuation of study drug to research staff who are conducting follow up data collection. The Principal Investigator should not be contacted by the site investigator, as intentional discontinuation of study drug implies a safety outcome, and the Principal Investigator should be blind to all study outcomes. Discontinuation of study drug is not a withdrawal from the study.

If study subjects are hospitalized after discharge to home from the emergency department, this is not a reason for discontinuation of study drug. We estimate that only a small proportion (<5%) of enrolled children will be hospitalized after discharge to home from the emergency department. If that does occur, subjects should continue on the study protocol unless they have developed an exclusion criterion for study drug administration (e.g. critical illness, immunosuppression, etc.). To facilitate continuation of study drug administration during hospitalization, parents are provided with a letter for their pediatrician. The letter will describe the study, the care-plan, and it will include site investigator contact information and the importance of adhering to the study protocol. The parents will be instructed to share this letter with their pediatrician and with the admitting physician.

Breaking the blinding of the study drug should not be necessary, but an unblinding procedure will be available. This will require contacting the Medical Monitor at the Data Coordinating Center. However, unblinding is almost never necessary. The primary anticipated reason for a clinician requesting unblinding is the suspected occurrence of invasive infection (bacteremia/septicemia or meningitis), and the natural desire to know if the subject was receiving active Lactobacillus rhamnosus GG, ATCC 53103 (LGG). The initial recommendation from the Medical Monitor will always be that the clinician should assume that the subject was receiving active drug, and include Lactobacillus

Protocol 032 (Schnadower) Page 30 of 49

rhamnosus in the differential diagnosis (and treatment coverage) of the suspected invasive infection. Lactobacillus rhamnosus is readily susceptible to common antibiotics, and the

Protocol 032 (Schnadower) Page 20 of 49

differential diagnosis of subsequent invasive infection would include organisms other than Lactobacillus rhamnosus. Thus, future clinical treatment should not be dependent on unblinding information.

If the situation requires unblinding, the Medical Monitor and Data Coordinating Center staff will provide the information, and the unblinding will be documented. Study drug will be discontinued, but the subject should not be withdrawn from the study! All study procedures (except study drug administration) should be continued, as the subsequent clinical course of the subject is critical to the science of the trial. All unblinding events will be reported in the closed session of the next scheduled Data Safety Monitoring Board meeting. Clinical staff and parents should not report the unblinding to research staff who are conducting follow up data collection. The Principal Investigator should not be contacted by the site investigator, as intentional unblinding of a subject implies a safety outcome, and the Principal Investigator should be blind to all study outcomes.

4.4 Withdrawal from Study

Parents may completely withdraw their child from participation in this study at any time, including discontinuation of data collection. However, withdrawal from study is a completely different issue from discontinuation of study drug, and true withdrawal from study is an exceedingly rare event. If parents demand withdrawal of their child from the study, research staff will attempt to obtain adverse event information as required by the Food and Drug Administration (all adverse events for 30 days, monthly follow up for long term adverse events).

4.5 Stool Sample Testing

Stool sample swabs will be collected from all study subjects, if possible, for polymerase chain reaction (PCR) analysis. Once obtained, swabs will be frozen and sent to a central laboratory for analysis. A storage and shipping protocol will be provided in the study manual of operations. Samples will be tested with multiplex PCR. Sites will also be asked to collect an optional bulk sample of stool if one is available at the time of enrollment (i.e. fresh diaper, etc). This will be used for future studies.

Protocol 032 (Schnadower) Page 21 of 49

In addition, at the lead site, Washington University, study research personnel will collect and freeze subjects' bulk stool specimens in the acute phase (within 24 hours of presentation) and 14 days after presentation using a previously tested bulk stool specimen collection protocol. Specimens may be collected in the emergency department or the

families will be given a specimen collection kit, gel packs and a metallic envelope. When the specimen is ready for collection, a courier will pick up the specimen and cool pack at the patient's home and deliver it to a logistics collection center at Washington University School of Medicine. This service is available 24 hours a day, year round. The stools will be stored as a part of a future project to assess the potential mechanisms of action of Lactobacillus rhamnosus GG, ATCC 53103 (LGG) in acute gastroenteritis.

5 Data Analysis

The hypotheses of this study are:

1. In children with acute gastroenteritis, probiotic administration in the emergency department will be associated with a clinically-important decrease in the proportion of children suffering from moderate-severe disease, defined by a validated Modified Vesikari Score 9, compared to placebo.

2. In children with acute gastroenteritis, probiotic administration will not be associated with serious adverse events, and will have a similar rate of side effects (e.g. bloating, fever) as compared to placebo-treated children.

All analyses will be undertaken by the intention-to-treat (ITT) principle, except for adverse events, which will use the as-treated principle (compare the subjects based on the treatment regimen that they received). Patients who drop out or inadvertently crossover will be followed and included in the ITT analysis. All statistical tests of hypotheses will be two-sided. Baseline characteristics will be analyzed to determine if there is a need to adjust for differences between groups in exploratory analyses. Sensitivity analyses will be performed to assess the possibility and consequences of non-random loss to follow-up. The proportions of children experiencing an unscheduled healthcare visit or any potential adverse effect, as reported by the caregivers, will be compared between groups using the Mantel-Haenszel test, stratified by site. The analysis will evaluate the presence/absence of pre-specified side effects, as an aggregate outcome variable. A per-protocol analysis will be conducted to provide additional insight as non-compliance may result in an underestimation of the benefits of probiotics in the ITT analysis.[72]

5.1 Specific Aim Analyses

Protocol 032 (Schnadower) Page 23 of 49

Specific Aim 1. Determine the clinical effectiveness of probiotic administration to reduce morbidity in children presenting to the emergency department with acute

Probiotics Study Protocol Version 3.00
Protocol Version Date: February 13, 2017
The EMSC Pediatric Emergency Care Applied Research Network

Protocol 032 (Schnadower) Page 24 of 49

gastroenteritis.

The primary efficacy endpoint of this study is the presence of moderate-severe acute gastroenteritis, as defined by a total post-enrollment Modified Vesikari Score ≥ 9 during the 2-week follow-up period. Each of the 7 items in the score is tabulated individually (maximum of 20 points); the sum of these individual variables represents the total Modified Vesikari Score. At the time of randomization a baseline Modified Vesikari Score will be assigned based on symptoms prior to randomization. This baseline score will serve as a covariate in a secondary analysis of the primary outcome. The post-enrollment Modified Vesikari Score which will be employed to determine the presence/absence of the primary efficacy outcome, is based only on symptoms that occur between randomization and day 14, the conclusion of the study period for this outcome. Only symptoms and outcomes that occur following randomization will be included in the post-enrollment score.

The post-enrollment score is calculated only once, on day 14. At that time, each of the seven variables will be assigned a score for the entire study period (from randomization to day 14). Each variable will be scored by 1 of 3 methods:

1. Worst 24 hour period - maximal number of episodes of vomiting in a 24 hour period, maximal number of episodes of diarrhea in a 24 hour period, and maximal temperature;
2. Total duration of symptoms, including the number of days on which any gastroenteritis-related symptom occurred;
3. Occurrence of a treatment outcome or unscheduled subsequent healthcare utilization. If the patient was admitted directly following their enrollment ED visit and that admission stay is longer than 48 hours, it will be included as treatment in the post enrollment MVS. If the patient's admission stay was less than 48 hours, it will not be included in any MVS calculation.

The primary efficacy endpoint (the presence of moderate or severe disease, as defined by a total Modified Vesikari Score ≥ 9 during the 2 week follow-up period) will be limited to symptoms and outcomes that occur after randomization. If a patient's symptoms of vomiting and diarrhea stop during the same 24 hours, and then recur, that is not included in the post-enrollment score since that will be considered a new illness. In the original score, severe disease was defined as ≥ 11[61, 62, 73–75] and moderate as ≥ 9.[76] In our derivation and validation pilot studies,[12, 13] construct validity was demonstrated and validated by using scores of ≥ 9 to define moderate and ≥ 11 to define severe disease.

Protocol 032 (Schnadower) Page 25 of 49

These cut-points were associated with significant increases in other measures of disease severity such as degree of dehydration, likelihood of admission and daycare ($p = 0.01$)

and parental work absenteeism (p <0.001).[12, 13]

The proportion of children with moderate-to-severe disease (i.e. Modified Vesikari Score 9), the primary outcome, will be analyzed by comparing proportions utilizing a Mantel-Haenszel test, stratified by participating center and by duration of symptoms at enrollment. Significance for this primary outcome measure will be set at 0.05. Secondary analyses of the primary outcome will use logistic regression methods to adjust for covariates that may be imbalanced between groups, (e.g. age, pre-enrollment Modified Vesikari Score, hydration assessment, need for hospitalization at index visit). We will also analyze the outcome using Modified Vesikari Score as a continuous variable through a stratified Wilcoxon rank-sum test and compare the results with the primary analysis.

Specific Aim 2. Determine the safety and side effect profiles of Lactobacillus rhamnosus GG, ATCC 53103 (LGG) in children presenting to the emergency department with acute gastroenteritis.

The primary safety endpoint of this study is the occurrence of invasive disease (including meningitis and bacteremia) from Lactobacillus rhamnosus. The secondary safety outcome will be the presence of any adverse events. For more information on adverse events, see Section 8.3 on page 33. Adverse events (including serious) will be tabulated by study arm for DSMB reporting and for final analysis of the safety of Lactobacillus rhamnosus GG, ATCC 53103 (LGG) in this setting. Adverse events will also be coded using the MedDRA vocabulary.

Side Effects: Occurrence of side effects such as weakness, bloating, gas, intestinal rumbling, diarrhea, blood in stool, abdominal pain, abdominal cramps, nausea, vomiting, loss of appetite, heartburn, constipation, skin rash, fever, nasal congestion, sore throat, cough, headache, muscle aches, chills, and diaper rash. These will be tabulated and compared by study arm.

5.2 Secondary Study Outcomes

Secondary outcomes will include three of the individual components of the Modified Vesikari Score considered independently and one additional measure of burden of illness. Specifically, they are:

1. Diarrhea duration: Time from randomization until the appearance of the last watery stool as reported during daily surveys.

Protocol 032 (Schnadower) Page 27 of 49

2. Vomiting duration: Time from randomization until the last vomiting episode as reported during daily surveys. Vomiting duration is only evaluated in children who vomited 3 times during the 24 hours prior to the emergency department visit.

Protocol 032 (Schnadower) Page 28 of 49

3. **Return visits:** Return visits for unscheduled care to a healthcare provider related to vomiting, diarrhea, dehydration, fever, or fluid refusal, within 2 weeks of the index visit. Scheduled visits (e.g. reassessment, vaccinations, unrelated issues) will not be included.

4. **Missed daycare:** Days of daycare missed by subjects who attend daycare.

5. **Missed work:** Days of work missed by caregivers who work outside of the home.

6. **Household transmission rate:** A household census will be obtained at the time of enrollment, and information about household contacts symptoms will be obtained during daily surveys to determine household transmission rate.

The overall significance level for statistical tests on the secondary outcomes will be set at 0.05. Holm's method will be used to adjust for multiple comparisons.[77] The continuous variables of duration of diarrhea and duration of vomiting (measured in hours and analyzed with a Van Elteren test),[78] will be stratified by clinical center. Similarly, the number of days the child is absent from daycare and the caregiver is absent from work will be analyzed with a Van Elteren test, stratified by clinical center and etiology.

Dichotomous outcomes to be evaluated include emergency department acute gastroenteritis-related revisits, intravenous rehydration, and hospitalization. Additional analyses involving these outcomes will include linear and logistic regression models that adjust for possible e↵ects of baseline characteristics.

5.3 Sample Size Calculations and Statistical Power

The primary analysis will be performed on a binary outcome: development of moderate-to-severe disease. The power of this analysis is based on the proportion of patients with moderate-to-severe disease. Our pilot data[12, 13] indicate that 25% of patients will have moderate to severe disease during the course of their illness. Furthermore expert surveys indicated that an absolute risk reduction of 10% would constitute a minimal clinically-important di↵erence (MCID). Therefore our sample size calculation assumed a 25% event rate in the control group for which we desire to detect an absolute beneficial treatment e↵ect of 10% with 90% power. Using a two-sided type I error (↵) of 0.05 and the hypothesized proportions yields a required total sample size of 670 patients.[79] Our expected power, should we find di↵erent event rates in our 2 groups, is displayed in Table 2 on the next page.

Protocol 032 (Schnadower) Page 29 of 49

Based on previous work by our group,[70, 71, 80] we assumed a 10% loss to follow up (670/0.90 = 744), 5% drop out, and 3% drop in (caregivers who buy a probiotic agent

Probiotics Study Protocol Version 3.00
Protocol Version Date: February 13, 2017
The EMSC Pediatric Emergency Care Applied Research Network

Protocol 032 (Schnadower) Page 30 of 49

Outcome Control	Outcome Intervention	% Difference	Power
0.30	0.21	9%	0.76
0.30	0.20	10%	0.85
0.25	0.15	10%	0.90
0.25	0.16	9%	0.82
0.25	0.17	8%	0.72
0.20	0.10	10%	0.95
0.20	0.12	8%	0.81
0.20	0.13	7%	0.69

Table 2: Power Analysis

to administer to their child) rate ($744/(0.92)^2 = 879$). Adjustment for O'Brien-Fleming monitoring boundaries requires a further 2% increase. Thus, the total number randomized (final sample size) will be 897.

5.3.1 Enrichment Design

The above study design and power analysis are based on the assumption of homogeneous treatment effect. We may also incorporate an enrichment design[81, 82] to restore the statistical power in case the presence of a subpopulation with a substantially low treatment effect is identified. We are particularly interested in two potential subpopulations: participants with <2 days of symptoms and those with ≥2 days of symptoms. Based on our pilot data, each subpopulation accounts for approximately 50% of the total population. The decision for enrollment modification will be made at the first interim analysis for efficacy. Specifically, three statistics (based on a normal approximation of binomial distribution, or z-statistics) will be calculated to compare the primary efficacy endpoint between treatment and control groups for subjects in the total population and the two subpopulations, respectively. If the z-statistic from a subpopulation is <0.3 and also smaller than that in the total population, subjects from this subpopulation will no longer be considered in the subsequent enrollment. All subjects, regardless of symptom duration will be included in the final analyses. Our simulation studies have shown that such an enrichment design can increase the power considerably when the treatment effects

Protocol 032 (Schnadower) Page 31 of 49

are different across subpopulations, while it will have little impact on power when the treatment effects are similar.

5.3.2 Sub-group Analyses

The presence of a Modified Vesikari Score 9 will be analyzed by age <1 year, duration of symptoms, breast-feeding status, antibiotic usage, rotavirus and norovirus positivity. A subgroup effect will be considered significant if the interaction between subgroup and treatment in a logistic regression model is significant at a Bonferroni-corrected level.

5.4 Recruitment Estimates and Attrition

5.4.1 Recruitment

To estimate the number of subjects with acute gastroenteritis of eligible ages that would meet all inclusion criteria and be enrolled annually, we obtained discharge diagnoses from all study sites for 2005-2011. In 2011, the study sites evaluated 14,048 potentially eligible subjects (patients 3-48 months with ICD9 codes for diarrhea) out of 507,039 total emergency department visits (2.8%). In the past 7 years the lowest proportion of such patients per total emergency department volume was 2.3% (2008). Therefore, using 2011 emergency department volumes and lowest proportion of patients with diarrhea our conservative estimate for eligible patients would be 11,150 patients 3-48 months with diarrhea per year (or 30 patients per day network wide). Our best estimate is that 4.5% of children with acute gastroenteritis aged 3-48 months will be enrolled. We plan to enroll 225 patients/year and 970 subjects over 4 acute gastroenteritis seasons.

5.4.2 Potential for Bias

Reporting bias will be minimized by adhering to CONSORT recommendations including the use of third-party assignment.[83] Placebo capsules and active drug will be provided by I-Health Inc. The total weight of all capsules is 325 mg. The probiotic and placebo capsules and powder are identical in appearance, taste, texture, and odor. Participants, families, healthcare providers, data collectors, outcome adjudicators, and data analysts will be blinded as to intervention arm, thereby preventing bias in outcome assessment. Two DCC statisticians will be partially unblinded (with knowledge of group assignments, but not group identities) in order to present interim results to the DSMB. An intention- to-treat analysis will be performed to minimize bias associated with poor compliance and non-random loss of participants.[84] Co-interventions (e.g. antiemetic administration, intravenous rehydration) and other potential sources of

confounding will be recorded. Our use of a published validated score as an outcome measure will protect against the introduction of bias in the assessment of treatment effects.[85]

Protocol 032 (Schnadower) Page 34 of 49

5.4.3 Compliance

Noncompliance with probiotics is rarely reported in previous studies, and not expected to be problematic in this cohort.[86, 87] Participant withdrawal has been related mostly to the primary illness.[19] In the Canadian pilot study, compliance was 91%. We will track compliance by obtaining information from parents during the day 5 follow-up contact. If there are any unused capsules, we will request their return (a pre-stamped, pre-addressed envelope will be provided).

5.4.4 Loss to Follow-up

Our previous emergency department pediatric acute gastroenteritis research studies achieved telephone follow-up rates of 98% on day 3 and 96% on day 7.[70] Similar success has been documented in other PECARN multicenter studies (91%).[80] We estimate a 10% loss to follow-up. If daily contact does not occur, we will collect data from missed days on subsequent days when caregivers are contacted. The use of patient diaries (paper and/or electronic) and chart review will supplement parent contact. Based on our pilot trial we have devised the following strategies to maximize follow-up: all follow-up procedures will be centralized at the lead institution and conducted by experienced study staff; we will obtain multiple phone numbers from caregivers as well as emergency contacts, and we will schedule calls and send reminders prior to the call if preferred. Finally, electronic diary filing and transmission may be available for interested families.

5.4.5 Compensation

Financial compensation may be provided to compensate for parent's time completing follow-up. This compensation must be approved by each site's Institutional Review Board.

6 Data Management

6.1 Clinical Sites

Study data will be recorded on paper work forms or by direct data entry into an electronic data capture (EDC) system. If paper work forms are used they will be retained at the clinical site. The data from the work forms will then be entered into the EDC system

Protocol 032 (Schnadower) Page 35 of 49

provided by the Data Coordinating Center at the University of Utah School of Medicine.

The clinical investigator at each participating site will complete a Form FDA 1572, Statement of Investigator. That individual is responsible for all aspects of study imple-

Probiotics Study Protocol Version 3.00
Protocol Version Date: February 13, 2017
The EMSC Pediatric Emergency Care Applied Research Network

mentation, including administration of study drug, collection of accurate study data, and correct entry of the data into the EDC. These tasks may be specifically delegated to other individuals at the clinical site, but the clinical investigator is responsible to supervise all aspects of the study, and is responsible to assure that all staff involved in this study are adequately trained to perform the delegated tasks. All local research records will remain in a locked file in a secure room unless being used by research staff, and all computerized information will be maintained on password protected computers.

6.2 PECARN Data Coordinating Center (Utah)

In addition to locally secured, identifiable information, partially identifiable information for all sites will be maintained at the PECARN Data Coordinating Center, located at the University of Utah in Salt Lake City, Utah. The Data Coordinating Center has a state-of-the-art computer infrastructure with a dedicated server room with a fire suppression system, air conditioning, cooling system and separate air filtering. The server facility is locked separately from the remainder of the Data Coordinating Center and access to the building is monitored by security personnel year round. The Data Coordinating Center coordinates its network infrastructure and security with the Health Sciences Campus (HSC) information systems at the University of Utah. This provides the Data Coordinating Center with effective firewall hardware, automatic network intrusion detection, and the expertise of dedicated security experts working at the University.

Network equipment includes four high-speed switches. User authentication is centralized with two Windows 2008 domain servers. Communication over public networks is encrypted with virtual point-to-point sessions using SSL or VPN technologies, both of which provide at least 128 bit encryption. OpenClinica (Web-based clinical studies data management system), eRoom™ (Web-based collaborative workspace) and other web applications use the SSL protocol to transmit data securely over the Internet. Direct access to Data Coordinating Center machines is only available while physically located inside the Data Coordinating Center offices, or via a VPN client. All network traffic is monitored for intrusion attempts, security scans are regularly run against our servers, and IT staff are notified of intrusion alerts.

Production servers running mission critical applications are clustered and configured for failover events. Servers are backed up with encryption through a dedicated backup server that connects across an internal 10 gigabit network to a tape drive. Storage area

Protocol 032 (Schnadower) Page 37 of 49

networking (SAN) applications, clusters, and switch-to-switch links are also on a 10 gigabit network. Incremental backups occur hourly during the week. Incremental backups

Protocol 032 (Schnadower) Page 38 of 49

also are performed nightly with full system backups occurring every week. Tapes are stored in a fireproof safe inside the data center facility, and full backups are taken to an off-site commercial storage facility. Security is maintained with Windows 2008 user/group domain-level security.

Users are required to change their passwords every 90 days, and workstations time out after 5 minutes of inactivity. All files are protected at group and user levels; database security is handled in a similar manner with group level access to databases, tables, and views in Microsoft SQL Server. All portable computers are whole-disk-encrypted.

6.3 Data Confidentiality

The PI and other research personnel have all completed training and received certification in Human Subjects Research Protection and HIPAA. All project staff hired will also successfully complete this training prior to engaging in any research or treatment with study participants and renew this training as required by their institution.

The investigators and staff of the Data Coordinating Center are fully committed to the security and confidentiality of all data collected for PECARN studies. All Data Coordinating Center personnel at the University of Utah have signed confidentiality agreements concerning all data encountered in the center. Violation of these agreements may result in termination from employment at the University of Utah. In addition, all personnel involved with data coordinating center data systems have received Human Subjects Protection and HIPAA education.

The staff, reviewers and investigators involved with this study will be required to sign agreements from the Data Coordinating Center that relate to maintenance of passwords, information system security, and data confidentiality.

6.4 Data Quality Management and Monitoring

Protocol 032 (Schnadower) Page 39 of 49

The Data Coordinating Center monitors PECARN studies on behalf of the investigators and the funding agency. The purposes of monitoring include demonstration of adherence to human subjects protection requirements and assurance of high quality study data. Monitoring is usually done remotely and may also involve physical site monitoring visits. Site monitoring is described in more detail in Section 9.2.

Protocol 032 (Schnadower) Page 30 of 49

6.5 Record Access

The medical record and study files (including informed consent, permission, and assent documents) must be made available to authorized representatives of the Data Coordinating Center, upon request, for source verification of study documentation. In addition, medical information and data generated by this study must be available for inspection upon request by representatives of the the National Institutes of Health, Food and Drug Administration, and the Institutional Review Board (IRB) for each study site.

7 Protection of Human Subjects

7.1 Institutional Review Board (IRB) Approval

The Data Coordinating Center and each clinical center must obtain approval from their respective IRB prior to participating in the study. The Data Coordinating Center will track IRB approval status at all participating centers and will not permit subject enrollment without documentation of initial IRB approval and maintenance of that approval throughout subsequent years of the project.

7.2 Informed Consent

This protocol requires that parents or other legally empowered guardians sign a parental permission form. The parent or legal guardian will be informed about the objectives of the study and the potential risks. Parents or legal guardians of eligible children with symptoms of gastroenteritis will be approached to provide permission for their child's participation in the study. Parental or guardian permission will be obtained prior to initiation of study activities. Documentation of parental permission will be maintained at the study site. As the maximum eligible age is 48 months, child assent is not applicable.

7.3 Potential Risks

Protocol 032 (Schnadower) Page 31 of 49

Lactobacilli are ubiquitous in the human diet and are a large part of the over 1 trillion live bacteria that reside in the gastrointestinal tract of healthy individuals. Overall, Lactobacillus rhamnosus GG, ATCC 53103 (LGG) is well tolerated in the pediatric patient population. Infections (bacteremia, endocarditis, pneumonia, deep abdominal abscesses) have been reported in sick neonates, severely debilitated and immune-compromised individuals given probiotics. The use of probiotics in these individuals continues to be controversial; however prospective studies have been conducted in adults and children

Protocol 032 (Schnadower) Page 32 of 49

with HIV as well as preterm neonates, with no reported systemic infections. Finally, there are 7 case reports of invasive disease after administration of Lactobacillus rhamnosus GG, ATCC 53103 (LGG). These occurred in patients in intensive care units, in patients with central venous catheters, or in patients who are on immunosuppressive therapy, have short gut syndrome, or are at risk for endocarditis. These risk factors are exclusion criteria from our study population.

The clinical risks of invasive infection from Lactobacillus rhamnosus are related to receiving Lactobacillus rhamnosus GG, ATCC 53103 (LGG). There are risks associated with acute gastroenteritis that are not related to study participation, including dehydration, electrolyte abnormalities, systemic infection and co-infection, and hospital admission. All subjects will be clinically treated for acute gastroenteritis in accordance with local site treatment protocols. There may be other unknown risks of Lactobacillus rhamnosus GG, ATCC 53103 (LGG) in children with acute gastroenteritis.

Loss of confidentially is always a risk in a study, but safeguards are in place to protect against this.

7.4 Protections Against Potential Risks

Several steps will be taken to minimize the risks of participation in the study. All of the participating clinical centers are tertiary pediatric hospitals with highly trained pediatric staff. Subjects will be closely followed after discharge. Families will be contacted daily until both the diarrhea and vomiting have resolved and the treatment has been completed. Additional contact will be made on day 14, and months 1, 3, 6, 9 and 12 for long term safety outcomes. Parents or legally authorized representatives will also receive specific instructions as to when to see a health care provider. If an adverse event is reported at the time of follow-up, research personnel will refer subjects for appropriate medical care, when applicable.

Loss of confidentiality will be mitigated by the use of the PECARN Data Coordinating Center which has a highly secure IT infrastructure, and by the existence of trained research staff at participating sites. Data security is described in Section 6.

Protocol 032 (Schnadower) Page 33 of 49

7.5 Potential Benefits

Subjects participating in the study may benefit directly by experiencing less severe disease, shorter duration of symptoms, and decreased need for further health care utilization.

Protocol 032 (Schnadower) Page 34 of 49

There may also be reduced symptoms (i.e. diarrhea, vomiting, fever) and morbidity, thereby diminishing acute gastroenteritis-associated resource consumption (e.g. physician visits, hospitalization) and societal costs. Additional benefits in the study include closer monitoring of symptoms and potentially earlier recognition of any worsening of acute gastroenteritis due to the frequency of telephone follow up and communication with the family. The knowledge gained in this study may lead to improved treatment options for gastroenteritis for other children and lead to new therapeutic options for future patients. Subjects and their families may therefore benefit indirectly by participating in research with the potential to provide subsequent benefit to others. Finally, future analysis of bulk stool specimens will enable a better understanding of potential mechanisms of action of Lactobacillus rhamnosus GG, ATCC 53103 (LGG), which will likely provide benefit to future patients.

8 Data and Safety Monitoring Plan

8.1 Data Safety Monitoring Board (DSMB)

This study will have a Data Safety Monitoring Board (DSMB) appointed in accordance with instructions from the NICHD program officer. The DSMB will have a charter, will approve the protocol prior to implementation, and will review interim analyses for safety and efficacy.

The purpose of the DSMB is to advise the Federal funding agency (NICHD), the study Principal Investigator (Dr. Schnadower), and the PECARN Steering Committee regarding the continuing safety of study subjects and the continuing validity and scientific merit of the study. The DSMB is responsible for monitoring accrual of study subjects, adherence to the study protocol, assessments of data quality, performance of individual clinical sites, review of serious adverse events and other subject safety issues, and review of formal interim statistical analyses of treatment efficacy.

The Data Coordinating Center will send reports relating to these topics to DSMB members prior to each DSMB meeting. The Data Coordinating Center will staff DSMB meetings. The production and approval of DSMB minutes will be done in accordance with requirements of the NICHD. Each DSMB meeting will have a summary recommendation that will be provided to each participating clinical site for submission to the local

Protocol 032 (Schnadower) Page 35 of 49

Institutional Review Board (IRB). More detailed information from the DSMB meetings will not be routinely provided for local IRB submission.

Protocol 032 (Schnadower) Page 36 of 49

8.2 Frequency of Interim Analysis:

The Data Safety Monitoring Board (DSMB) will meet after 80 patients (safety end points for 1 month), approximately 350 patients (end of 2nd year of enrollment) and 620 patients (end of 3rd year of enrollment) to review enrollment, study procedures, loss to follow-up, drop-in rate, and interim safety and efficacy results. The analyses will test the hypothesis that the probability of developing moderate-to-severe acute gastroenteritis in the probiotic arm is equal to that in the placebo arm. An analysis will also be conducted after 20 subjects under 6 months of age have been enrolled. The DSMB is to review interim data and make recommendations regarding continuation or modification of the study based on safety in this age group. Conservative O'Brien-Fleming monitoring boundaries, implemented using the Lan-DeMets α-spending function approach, will be used as guidelines for early stopping for efficacy.

8.3 Adverse Event Reporting

8.3.1 Definitions, Relatedness, Severity and Expectedness

Definition: An adverse event (AE) is any untoward medical occurrence experienced by a subject. An event constitutes a disease, a set of related signs or symptoms, or a single sign or symptom. On each study day, the site investigators will evaluate adverse events. Study staff will obtain information on symptoms and adverse events on scheduled follow up calls. Adverse events not previously documented in the study will be recorded on the adverse event record form. The nature of each experience, date and time (where appropriate) of onset, outcome, course, and relationship to treatment should be established.

Side Effects: Occurrence of side effects such as weakness, bloating, gas, intestinal rumbling, diarrhea, blood in stool, abdominal pain, abdominal cramps, nausea, vomiting, loss of appetite, heartburn, constipation, skin rash, fever, nasal congestion, sore throat, cough, headache, muscle aches, chills, and diaper rash. Study staff will obtain information about side effects on scheduled follow up contacts.

Relatedness: The suspected relationship between study interventions and any adverse event will be determined by the site investigator using the following criteria. Relatedness may **not** be assessed by a research coordinator, and must be assessed by an investigator.

Protocol 032 (Schnadower) Page 37 of 49

Not Related: The event is clearly related to other factors, such as the subject's clinical state, therapeutic interventions, or concomitant drugs administered to the subject.

Possibly Related: The event follows compatible temporal sequence from the time of beginning the assigned study intervention, but could have been produced by other factors such as the subject's clinical state, therapeutic interventions, or concomitant drugs administered to the subject.

Probably Related: The event follows a reasonable temporal sequence from the time of beginning the assigned study intervention, and cannot be reasonably explained by other factors such as the subject's clinical state, therapeutic interventions, or concomitant drugs administered to the subject.

Some toxicities will be difficult to distinguish from abdominal symptoms related to acute gastroenteritis (such as bloating, abdominal pain, diarrhea, fever and diaper rash), and only at the time of analysis will we be able to determine whether these signs and symptoms are different between the groups.

Seriousness: The severity of clinical adverse events and laboratory abnormalities will be recorded by the site investigator and categorized. A serious adverse event (SAE) is an adverse event that:

- results in death; or
- is life-threatening (the patient was, in the view of the site investigator, in immediate danger of death from the event as it occurred); or
- requires inpatient hospitalization or prolongs an existing hospitalization; or
- results in persistent or significant disability or incapacity; or
- results in congenital anomaly/birth defect; or
- any other event that, based upon appropriate medical judgment, may jeopardize the subject's health and may require medical or surgical intervention to prevent one of the other outcomes listed in this definition.

Expectedness of the Event: All adverse events, including serious adverse events, will be evaluated as to whether their occurrence was expected or unexpected. An adverse event is considered expected if it is known to be associated with acute gastroenteritis (acute gastroenteritis), other underlying medical conditions of the subject, is directly related to study outcome, or is otherwise mentioned in the protocol, informed consent, investigator brochure, or other study documents. Expected complications of acute gastroenteritis include abnormal taste, malaise, abdominal distention, foul smelling stools, dehydration,

Protocol 032 (Schnadower) Page 39 of 49

electrolyte and other laboratory abnormalities, other symptoms associated with viral syndromes, systemic infection and co-infection, seizures, and hospital admission.

Probiotics Study Protocol Version 3.00
Protocol Version Date: February 13, 2017
The EMSC Pediatric Emergency Care Applied Research Network

Protocol 032 (Schnadower) Page 40 of 49

Treatment or Action Taken: For each adverse event, the site investigator will record whether an intervention was required:

- Intervention: Surgery or procedure
- Other Treatment: Medication initiation, change, or discontinuation
- None: No action taken

Outcome of Event: Finally, the site investigator will record the clinical outcome of each adverse event as follows:

- Death
- Recovered and the patient returned to baseline status
- Recovered with permanent sequelae
- Symptoms continue

8.3.2 Time Period for Adverse Events

For purposes of this study, adverse events occur following randomization through 30 days after the last study drug dose will be recorded. Serious adverse events, unexpected medically attended events, and new onset chronic illnesses will be recorded from randomization through twelve months after the last study dose. Specifically, events that occur following parental permission to participate in the study, but prior to actual randomization, are not adverse events. These should be recorded as baseline conditions.

8.3.3 Data Collection Procedures for Adverse Events

After patient randomization, all adverse events (including serious adverse events), whether anticipated or unanticipated, will be recorded according to the date of first occurrence, severity, and their duration, as well as any treatment prescribed. Any medical condition present at the time of randomization, recorded in the patient's baseline history at study entry, which remains unchanged or improves, will not be recorded as an adverse event at subsequent evaluations. However, worsening of a medical condition that was present at the time of randomization will be considered a new adverse event and reported.

Protocol 032 (Schnadower) Page 41 of 49

Abnormal laboratory values that are clinically significant will be recorded as adverse events and the site investigator will assess the severity and relationship to the study. Laboratory values that are abnormal at the time of randomization and that do not worsen

Protocol 032 (Schnadower) Page 42 of 49

will not be recorded as adverse events.

Adverse events will be coded using the MedDRA coding vocabulary. Coding will be done centrally at the Data Coordinating Center because this requires specific training.

8.3.4 Unanticipated Problems (UP)

Unanticipated problems (UP) are defined as incidents, experiences, or outcomes that are unexpected, related to participation in the study, and suggest that the research places subjects at a greater risk of harm than was previously known or recognized. The site investigator will report unanticipated problems to the Data Coordinating Center within 24 hours. A detailed completed report will be required to be sent to the Data Coordinating Center within 3 working days of the event. After receipt of the complete report, the Data Coordinating Center will report these unanticipated problems to the NICHD Program Official or Project Officer in an expedited manner (within 24 hours). In accordance with local IRB requirements, the site investigator may be required to report such unanticipated problems to the IRB in addition to notifying the Data Coordinating Center. In the event that the medical monitor believes that such an event warrants emergent suspension of enrollment in the trial, and NICHD staff cannot be reached expeditiously, the Data Coordinating Center will notify the study investigator (Dr.Schnadower) and all site investigators to cease enrollment in the trial. Resumption of enrollment will not occur without consent of the NICHD staff after discussion with the DSMB.

8.3.5 Monitoring Serious Adverse Events

The Principal Investigator of the Data Coordinating Center (Dr. Dean) will act as the medical monitor for this study. If Dr. Dean is unavailable, a qualified physician will be designated to fulfill this function. Site investigators and/or research coordinators will report serious adverse events to the Data Coordinating Center within 24 hours. A detailed completed report will be required to be sent to the Data Coordinating Center within 3 working days of the event, and the medical monitor will assess all serious adverse events reported from site investigators.

For each of these serious adverse events, the site investigator will provide sufficient medical history and clinical details for a safety assessment to be made with regard to continuation of the trial. The medical monitor will sign each SAE report after review. All SAE reports will be retained at the Data Coordinating Center, and all SAE reports will be available for review by DSMB members and NICHD staff.

Protocol 032 (Schnadower) Page 43 of 49

In the unlikely event that the medical monitor believes an unexpected and study-related SAE warrants emergent cessation of enrollment in the trial, NICHD staff and the

DSMB chairperson will be immediately consulted. If these individuals concur with the judgment of the medical monitor, or if the NICHD staff and the DSMB chairperson cannot be reached expeditiously, the Data Coordinating Center will notify the study investigator (Dr.Schnadower) and all site investigators to cease enrollment in the trial. Resumption of enrollment will not occur without consent of the NICHD staff after discussion with the DSMB.

In accordance with local IRB requirements, the site investigator may be required to report such events to the IRB in addition to notifying the Data Coordinating Center.

After notification of the NICHD Program Official or Project Officer, and the DSMB chairperson, of serious, unexpected, and study-related adverse events or unanticipated problems (UP), decisions will be made whether to continue the study without change, and whether to convene the entire DSMB for an emergent meeting. If a decision is made to suspend enrollment in the trial, this will be reported to the study investigator (Dr. Schnadower) and all clinical investigators, who will be instructed to report this to their local IRB.

The DSMB will review all adverse events (not necessarily serious, unexpected, and study-related) during scheduled DSMB meetings. The Data Coordinating Center will prepare a Summary Report of Adverse Events for the DSMB meetings, classified with the MedDRA coding system.

8.3.6 Follow-up of Serious, Unexpected and Related Adverse Events

All serious, unexpected and related adverse events, that are unresolved at the time of the patient's termination from the study or discharge from the hospital, will be followed by the Clinical Center investigators until the events are resolved, subject is lost to follow-up, the adverse event is otherwise explained or has stabilized, or 12 months have passed from the time of last study dose.

9 Study Training and Monitoring

9.1 Study Training

A formal training program for investigators and research staff will be held prior to the start of enrollment. The training program will cover regulatory topics and Good Clinical Practice. The training will also provide in depth explanations regarding study procedures, clinical care, adverse event reporting, data entry procedures, quality assurance, site

Protocol 032 (Schnadower) Page 45 of 49

monitoring, and the informed consent process. A manual of operations will be provided to each Clinical Center investigator prior to the start of enrollment. The manual will

detail specific information about the study procedures, regulatory information, safety reporting, and other necessary information. Updates and revisions to the manual will be made available electronically. The Data Coordinating Center, in collaboration with the study investigator (Dr. Schnadower), will be the main contact for study questions.

9.2 Study Monitoring

The investigators recognize the importance of ensuring data of excellent quality. Site monitoring is critical to this process. Site monitoring has been a very effective tool for maintaining data quality in previous PECARN studies, and we will utilize this process to ensure excellent quality data in the proposed study. Our site monitoring plan is designed to identify problems with sites and methods for handling problems that arise. Site monitors must be provided with full access to study materials and the medical records for study subjects. If the medical records are in electronic form, the clinical investigator or an authorized individual must provide any assistance necessary to facilitate the site monitor's review of data in the electronic medical record.

9.2.1 Site Monitoring Plan

A supplemental study-specific monitoring plan, separate from the protocol will be completed which outlines specific criteria for monitoring. This plan will include the number of planned site visits, criteria for focused visits, or additional visits, a plan for chart review and a follow up plan for non-compliant sites. The monitoring plan also describes the type of monitoring that will take place (e.g. sample of all subjects within a site; key data or all data), the schedule of visits, how they are reported and a time frame to resolve any issues found. Remote site monitoring schedules will be determined by the Data Coordinating Center in coordination with the study principal investigator.

9.2.2 Clinical Site Monitoring

Site monitoring visits will be performed by a trained site monitor during the study period to ensure regulatory compliance, patient safety, and to monitor the quality of data collected. Essential document binders, regulatory documents and data collection forms may be reviewed. Interim visits will take place depending on grant budget, site enrollment, and compliance issues identified. The site monitor will provide each site with a written report, and sites will be required to follow up on any deficiencies. It is anticipated that the study monitoring visits for this protocol will consist of a site initiation visit (prior

Protocol 032 (Schnadower) Page 47 of 49

to patient enrollment), interim visits, and a close out visit. The site initiation may take place as group training made up of site investigators and research assistants.

Probiotics Study Protocol Version 3.00
Protocol Version Date: February 13, 2017
The EMSC Pediatric Emergency Care Applied Research Network

9.2.3 Remote Monitoring

The Data Coordinating Center may supplement on-site monitoring with remote monitoring activities. Remote monitoring involves detailed review of the data entered by the Clinical Center and consultations with the Clinical Center investigator and/or research coordinator to review safety and data quality. This may require uploading de-identified copies of specific parts of the medical record, patient study file, regulatory documentation, or other source documents to the Data Coordinating Center staff, who review those materials against the data recorded in the electronic data capture system. This helps assure protocol compliance and accurate data collection. The Data Coordinating Center may conduct more remote monitoring activities early in the trial to assure protocol compliance and identify any training issues that may exist. Remote monitoring the documents will be retained in accordance with federal requirements. Safety of subjects will be monitored and ensured in accordance with the Data and Safety Monitoring Board (DSMB) plan.

9.2.4 Pharmacy Monitoring

The Clinical Center pharmacy must maintain adequate records of all dispensed study drug. Each pharmacy may be monitored, either in person or remotely, and may be requested to send copies of these documents to the Data Coordinating Center. Since this study will use a central pharmacy, that pharmacy must also maintain adequate records and may also be monitored.

10 Regulatory Issues

10.1 Food and Drug Administration

This trial is being conducted under an Investigational New Drug application approved by the Food and Drug Administration (Investigational New Drug application #15371). The clinical investigator at each participating site will complete a Form FDA 1572, “Statement of Investigator.”

10.2 Health Insurance Portability and Accountability Act

The abstracted data will include limited identifiers as defined by the Health Insurance Portability and Accountability Act, and specific contact information will be provided to research staff conducting follow up with parents. Abstracted data will be retained and archived at the Data Coordinating Center in accordance with record retention requires of the Food and Drug Administration and the NIH. Contact information will not be

provided to the Data Coordinating Center (it will be provided directly to the central follow up research staff). For data analysis outside the Data Coordinating Center (e.g., when a public access database is made available), the Data Coordinating Center will create a completely de-identified analytical database for use by the study investigators, and for final archiving. All study sites have been or will be offered Business Associate Agreements with the University of Utah. Copies of signed Business Associate Agreements are maintained at the Data Coordinating Center.

10.3 Inclusion of Women and Minorities

The gender, ethnic and racial composition of patients enrolled in all PECARN studies is a function of the underlying referral population at each PECARN site participating in this trial. There will be no exclusion of patients based on gender, race, or ethnicity.

10.4 ClinicalTrials.gov Requirements

This trial has been registered at ClinicalTrials.gov (NCT #01773967). The title is "Impact of Emergency Department Probiotic (LGG) Treatment of Pediatric Gastroenteritis."

10.5 Retention of Records

For federally funded studies subject to the Common Rule, records relating to the research conducted shall be retained for at least 3 years after completion of the research. Completion of the research for this protocol should be anticipated to include planned primary and secondary analyses, as well as subsequent derivative analyses. Completion of the research also entails completion of all publications relating to the research. All records shall be accessible for inspection and copying by authorized representatives of the regulatory authorities at reasonable times and in a reasonable manner [45 CFR §46.115(b)].

Bibliography

[1] P. Coppens, M. F. da Silva, and S. Pettman. European regulations on nutraceuticals, dietary supplements and functional foods: a framework based on safety. Toxicology,

Protocol 032 (Schnadower) Page 41 of 49

221(1):59–74, 2006.

[2] L. G. Saldanha. US Food and Drug Administration regulations governing label claims for food products, including probiotics. Clin Infect Dis, 46 Suppl 2:S119–21; discussion S144–51, 2008.

[3] A. von Wright. Regulating the safety of probiotics–the European approach. Curr Pharm Des, 11(1):17–23, 2005.

[4] Cincinnati Children's Hospital. Use of Lactobacillus GG in children with acute gastroenteritis, 2011.

[5] S. T. Li, E. J. Klein, P. I. Tarr, and D. M. Denno. Parental management of childhood diarrhea. Clin Pediatr (Phila), 48(3):295–303, 2009.

[6] WHO. Diarrhoeal Disease Fact Sheet, 2009.

[7] CDC. CDC 2011 Estimates of Foodborne Illnesses in the United States, 2011.

[8] M. Senecal, M. Brisson, M. H. Lebel, J. Yaremko, R. Wong, L. A. Gallant, H. A. Garfield, D. J. Ableman, R. L. Ward, J. S. Sampalis, and J. A. Mansi. Measuring the Impact of Rotavirus Acute Gastroenteritis Episodes (MIRAGE): A prospective community-based study. Can J Infect Dis Med Microbiol, 19(6):397–404, 2008.

[9] CDC. Viral Gastroenteritis, 2011.

[10] CDC. Clinical Overview Norovirus Infection, 2013.

[11] D. Schnadower, S. B. Freedman, and P.I. Tarr. Unpublished Pilot Data for the Probiotic Study in 5 US EDs and 11 Canadian EDs, 2013.

[12] D. Schnadower, P. Tarr, M. Gorelick, K. O'Connell, C. Roskind, E. Powell, J. Rao, S. Bhatt, and S. Freedman. Validation of the Modified Vesikari Score in Children with Gastroenteritis in 5 U.S. Emergency Departments. Submitted to Journal of Pediatric Gastroenterology and Nutrition (manuscript in appendix), 2013.

[13] S. B. Freedman, M. Eltorky, and M. Gorelick. Evaluation of a gastroenteritis severity score for use in outpatient settings. Pediatrics, 125(6):e1278–85, 2010.

[14] A. E. Carroll and S. M. Downs. Improving decision analyses: parent preferences (utility values) for pediatric health outcomes. J Pediatr, 155(1):21–5, 25 e1–5, 2009.

[15] EJ Elliott. Acute gastroenteritis in children. BMJ, 334:7583, 2007.

[16] S. B. Freedman, S. Gouin, M. Bhatt, K. J. Black, D. Johnson, C. Guimont, G. Joubert, R. Porter, Q. Doan, R. van Wylick, S. Schuh, E. Atenafu, M. Eltorky, D. Cho, and A. Plint. Prospective assessment of practice pattern variations in the treatment of pediatric gastroenteritis. Pediatrics, 127(2):e287–95, 2011.

[17] S. B. Freedman, V. Sivabalasundaram, V. Bohn, E. C. Powell, D. W. Johnson, and K. Boutis. The treatment of pediatric gastroenteritis: a comparative analysis of pediatric emergency physicians′ practice patterns. Acad Emerg Med, 18(1):38–45, 2011.

[18] M. G. Gareau, P. M. Sherman, and W. A. Walker. Probiotics and the gut microbiota in intestinal health and disease. Nat Rev Gastroenterol Hepatol, 7(9):503–14, 2010.

[19] S. J. Allen, E. G. Martinez, G. V. Gregorio, and L. F. Dans. Probiotics for treating acute infectious diarrhoea. Cochrane Database of Systematic Reviews, 11:CD003048, 2010.

[20] Abigail F. Nixon, Sandra J. Cunningham, Hillel W. Cohen, and Ellen F. Crain. The e↵ect of Lactobacillus GG on acute diarrheal illness in the pediatric emergency department. Pediatr Emerg Care, 28(10):1048–1051, Oct 2012.

[21] H.: Szajewska, A. Skorka, M. Ruszczynski, and D. Gieruszczak-Bialek. Meta-analysis: Lactobacillus GG for treating acute diarrhoea in children. Aliment Pharmacol Ther, 25(8):871–81, 2007.

[22] Y. Vandenplas, S. G. De Hert, and PROBIOTICAL Study Group. Randomised clinical trial: the synbiotic food supplement Probiotical vs. placebo for acute gastroenteritis in children. Aliment Pharmacol Ther, 34(8):862–7, 2011.

[23] S. E. Co n, J. Elser, C. Marchant, M. Sawyer, B. Pollara, R. Fayorsey, L. Nelson, D. Lawley, M. Goveia, J. Stek, D. Hille, and M. J. DiNubile. Impact of acute rotavirus gastroenteritis on pediatric outpatient practices in the United States. Pediatr Infect Dis J, 25(7):584–9, 2006.

[24] E. J. Klein, D. R. Boster, J. R. Stapp, J. G. Wells, X. Qin, C. R. Clausen, D. L. Swerdlow, C. R. Braden, and P. I. Tarr. Diarrhea etiology in a Children’s Hospital Emergency Department: a prospective cohort study. Clin Infect Dis, 43(7):807–13, 2006.

[25] A. L. D’Souza, C. Rajkumar, J. Cooke, and C. J. Bulpitt. Probiotics in prevention of antibiotic associated diarrhoea: meta-analysis. BMJ (Clinical Research Ed.), 324(7350):1361, 2002.

Protocol 032 (Schnadower) Page 45 of 49

[26] C. K. King, R. Glass, J. S. Bresee, and C. Duggan. Managing acute gastroenteritis among children: oral rehydration, maintenance, and nutritional therapy. MMWR Recomm Rep, 52(RR-16):1–16, 2003.

[27] R. Khanna, M. Lakhanpaul, S. Burman-Roy, M. S. Murphy, Group Guideline Development, and team the technical. Diarrhoea and vomiting caused by gastroenteritis in children under 5 years: summary of NICE guidance. BMJ, 338:b1350, 2009.

[28] M. B. Katan. Why the European Food Safety Authority was right to reject health claims for probiotics. Benef Microbes, 3(2):85–9, 2012.

[29] V. Vero and A. Gasbarrini. The EFSA health claims 'learning experience'. Int J Food Sci Nutr, 63 Suppl 1:14–6, 2012. Vero, Vittoria Gasbarrini, Antonio England International journal of food sciences and nutrition Int J Food Sci Nutr. 2012 Mar;63 Suppl 1:14-6. Epub 2011 Nov 16.

[30] A. Flynn. Scientific substantiation of health claims in the EU. Proc Nutr Soc, 71(1):120–6, 2012.

[31] J. T. Heimbach. Health-benefit claims for probiotic products. Clin Infect Dis, 46 Suppl 2:S122–4; discussion S144–51, 2008.

[32] Markets and Markets. Global probiotics market worth us $32.6 billion by 2014, 2012.

[33] S Salminen, A Owehand, Y Benno, and YK Lee. Probiotics: how should they be defined? Trend Food Sci Technol, 10:3, 1999.

[34] A. R. Lomax and P. C. Calder. Probiotics, immune function, infection and inflammation: a review of the evidence from studies conducted in humans. Curr Pharm Des, 15(13):1428–518, 2009.

[35] L. J. Fooks and G. R. Gibson. Probiotics as modulators of the gut flora. Br J Nutr, 88 Suppl 1:S39–49, 2002.

[36] D. R. Mack, S. Michail, S. Wei, L. McDougall, and M. A. Hollingsworth. Probiotics inhibit enteropathogenic E. coli adherence in vitro by inducing intestinal mucin gene expression. Am J Physiol, 276(4 Pt 1):G941–50, 1999.

[37] S. Hudault, V. Lievin, M. F. Bernet-Camard, and A. L. Servin. Antagonistic activity exerted in vitro and in vivo by Lactobacillus casei (strain GG) against Salmonella typhimurium C5 infection. Appl Environ Microbiol, 63(2):513–8, 1997.

Protocol 032 (Schnadower) Page 47 of 49

[38] P. Naaber, R. H. Mikelsaar, S. Salminen, and M. Mikelsaar. Bacterial translocation, intestinal microflora and morphological changes of intestinal mucosa in experimental models of Clostridium di cile infection. J Med Microbiol, 47(7):591–8, 1998.

[39] G. R. Gibson and X. Wang. Enrichment of bifidobacteria from human gut contents by oligofructose using continuous culture. FEMS Microbiol Lett, 118(1-2):121–7, 1994.

[40] M. Miettinen, A. Lehtonen, I. Julkunen, and S. Matikainen. Lactobacilli and Streptococci activate NF-kappa B and STAT signaling pathways in human macrophages. J Immunol, 164(7):3733–40, 2000.

[41] J. M. Wong, R. de Souza, C. W. Kendall, A. Emam, and D. J. Jenkins. Colonic health: fermentation and short chain fatty acids. J Clin Gastroenterol, 40(3):235–43, 2006.

[42] I. R. Sanderson. Dietary modulation of GALT. J Nutr, 137(11 Suppl):2557S–2562S, 2007.

[43] V. Rosenfeldt, E. Benfeldt, N. H. Valerius, A. Paerregaard, and K. F. Michaelsen. Effect of probiotics on gastrointestinal symptoms and small intestinal permeability in children with atopic dermatitis. Journal of Pediatrics, 145(5):612–616, 2004.

[44] M. Olivares, M. P. Diaz-Ropero, N. Gomez, F. Lara-Villoslada, S. Sierra, J. A. Maldonado, R. Martin, J. M. Rodriguez, and J. Xaus. The consumption of two new probiotic strains, Lactobacillus gasseri CECT 5714 and Lactobacillus coryniformis CECT 5711, boosts the immune system of healthy humans. Int Microbiol, 9(1):47–52, 2006.

[45] B. L. Chiang, Y. H. Sheih, L. H. Wang, C. K. Liao, and H. S. Gill. Enhancing immunity by dietary consumption of a probiotic lactic acid bacterium (Bifidobacterium lactis HN019): optimization and definition of cellular immune responses. Eur J Clin Nutr, 54(11):849–55, 2000.

[46] C. Mullie, A. Yazourh, H. Thibault, M. F. Odou, E. Singer, N. Kalach, O. Kremp, and M. B. Romond. Increased poliovirus-specific intestinal antibody response coincides with promotion of Bifidobacterium longum-infantis and Bifidobacterium breve in infants: a randomized, double-blind, placebo-controlled trial. Pediatr Res, 56(5):791–5, 2004.

Protocol 032 (Schnadower) Page 49 of 49

[47] E. Tiollier, M. Chennaoui, D. Gomez-Merino, C. Drogou, E. Filaire, and C. Y. Guezennec. Effect of a probiotics supplementation on respiratory infections and immune and hormonal parameters during intense military training. Mil Med, 172(9):1006–11, 2007.

Probiotics Study Protocol Version 3.00
Protocol Version Date: February 13, 2017
The EMSC Pediatric Emergency Care Applied Research Network

[48] M. Rinne, M. Kalliomaki, H. Arvilommi, S. Salminen, and E. Isolauri. Effect of probiotics and breastfeeding on the bifidobacterium and lactobacillus/enterococcus microbiota and humoral immune responses. J Pediatr, 147(2):186–91, 2005.

[49] J. Z. Szajewska, H.: Mrukowicz. Probiotics in the treatment and prevention of acute infectious diarrhea in infants and children: a systematic review of published randomized, double-blind, placebo-controlled trials. J Pediatr Gastroenterol Nutr, 33 Suppl 2:S17–25, 2001.

[50] C. W. Van Niel, C. Feudtner, M. M. Garrison, and D. A. Christakis. Lactobacillus therapy for acute infectious diarrhea in children: a meta-analysis. Pediatrics, 109(4):678–84, 2002.

[51] J. S. Huang, A. Bousvaros, J. W. Lee, A. Diaz, and E. J. Davidson. Efficacy of probiotic use in acute diarrhea in children: a meta-analysis. Dig Dis Sci, 47(11):2625–34, 2002.

[52] S. J. Allen, B. Okoko, E. Martinez, G. Gregorio, and L. F. Dans. Probiotics for treating infectious diarrhoea. Cochrane Database Syst Rev, (2):CD003048, 2004.

[53] B. C. Johnston, L. Shamseer, B. R. da Costa, R. T. Tsuyuki, and S. Vohra. Measurement issues in trials of pediatric acute diarrheal diseases: a systematic review. Pediatrics, 126(1):e222–31, 2010.

[54] S. Guandalini. Probiotics for children with diarrhea: an update. Journal of clinical gastroenterology, 42 Suppl 2:S53–57, 2008.

[55] Daniel C. Payne, Mary Allen Staat, Kathryn M. Edwards, Peter G. Szilagyi, Jon R. Gentsch, Lauren J. Stockman, Aaron T. Curns, Marie Griffin, Geoffrey A. Weinberg,
Caroline B. Hall, Gerry Fairbrother, James Alexander, and Umesh D. Parashar. Active, population-based surveillance for severe rotavirus gastroenteritis in children in the United States. Pediatrics, 122(6):1235–1243, Dec 2008.

[56] H. Szymanski, J. Pejcz, M. Jawien, A. Chmielarczyk, M. Strus, and P. B. Heczko. Treatment of acute infectious diarrhoea in infants and children with a mixture of three Lactobacillus rhamnosus strains - A randomized, double-blind, placebo-controlled trial. Alimentary Pharmacology and Therapeutics, 23(2):247–253, 2006.

[57] Donna M. Denno, Nurmohammad Shaikh, Jenny R. Stapp, Xuan Qin, Carolyn M. Hutter, Valerie Hoffman, Jody C. Mooney, Kelly M. Wood, Harold J. Stevens, Robert

Protocol 032 (Schnadower) Page 51 of 49

Jones, Phillip I. Tarr, and Eileen J. Klein. Diarrhea etiology in a pediatric emergency department: a case control study. Clin Infect Dis, 55(7):897–904, Oct 2012.

[58] L. Vernacchio, R. M. Vezina, A. A. Mitchell, S. M. Lesko, A. G. Plaut, and D. W. Acheson. Diarrhea in American infants and young children in the community setting: incidence, clinical presentation and microbiology. Pediatr Infect Dis J, 25(1):2–7, 2006.

[59] D. M. Denno, J. R. Stapp, D. R. Boster, X. Qin, C. R. Clausen, K. H. Del Beccaro, D. L. Swerdlow, C. R. Braden, and P. I. Tarr. Etiology of diarrhea in pediatric outpatient settings. Pediatr Infect Dis J, 24(2):142–8, 2005.

[60] T. Ruuska and T. Vesikari. Rotavirus disease in Finnish children: use of numerical scores for clinical severity of diarrhoeal episodes. Scand J Infect Dis, 22(3):259–67, 1990.

[61] M. Fruehwirth, U. Heininger, B. Ehlken, G. Petersen, B. Laubereau, I. Moll-Schler, I. Mutz, and J. Forster. International variation in disease burden of rotavirus gastroenteritis in children with community- and nosocomially acquired infection. Pediatr Infect Dis J, 20(8):784–791, Aug 2001.

[62] Noga Givon-Lavi, David Greenberg, and Ron Dagan. Comparison between two severity scoring scales commonly used in the evaluation of rotavirus gastroenteritis in children. Vaccine, 26(46):5798–5801, Oct 2008.

[63] G. M. Ruiz-Palacios, I. Perez-Schael, F. R. Velazquez, H. Abate, T. Breuer, S. C. Clemens, B. Cheuvart, F. Espinoza, P. Gillard, B. L. Innis, Y. Cervantes, A. C. Linhares, P. Lopez, M. Macias-Parra, E. Ortega-Barria, V. Richardson, D. M. Rivera-Medina, L. Rivera, B. Salinas, N. Pavia-Ruz, J. Salmeron, R. Ruttimann, J. C. Tinoco, P. Rubio, E. Nunez, M. L. Guerrero, J. P. Yarzabal, S. Damaso, N. Tornieporth, X. Saez-Llorens, R. F. Vergara, T. Vesikari, A. Bouckenooghe, R. Clemens, B. De Vos, M. O'Ryan, and Group Human Rotavirus Vaccine Study. Safety and e cacy of an attenuated vaccine against severe rotavirus gastroenteritis. N Engl J Med, 354(1):11–22, 2006.

[64] M. Iturriza Gomara, R. Simpson, A. M. Perault, C. Redpath, P. Lorgelly, D. Joshi, M. Mugford, C. A. Hughes, J. Dalrymple, U. Desselberger, and J. Gray. Structured surveillance of infantile gastroenteritis in East Anglia, UK: incidence of infection with common viral gastroenteric pathogens. Epidemiol Infect, 136(1):23–33, 2008.

[65] J. Diez-Domingo, J. M. Baldo, M. Patrzalek, P. Pazdiora, J. Forster, L. Cantarutti, J. Y. Pircon, M. Soriano-Gabarro, and N. Meyer. Primary care-based surveillance

Protocol 032 (Schnadower) Page 53 of 49

to estimate the burden of rotavirus gastroenteritis among children aged less than 5 years in six European countries. Eur J Pediatr, 170(2):213–22, 2011.

[66] I. Pruvost, F. Dubos, E. Chazard, V. Hue, A. Duhamel, and A. Martinot. The value of body weight measurement to assess dehydration in children. PLoS One, 8(1):e55063, 2013.

[67] H. I. Huppertz, J. Forster, U. Heininger, R. Roos, H. U. Neumann, and T. Hammerschmidt. The parental appraisal of the morbidity of diarrhea in infants and toddlers (PAMODI) survey. Clin Pediatr (Phila), 47(4):363–71, 2008.

[68] Eileen L. Yee, Mary Allen Staat, Parvin Azimi, David I. Bernstein, Richard L. Ward, Charles Schubert, David O. Matson, Reina M. Turcios-Ruiz, Umesh Parashar, Marc-Alain Widdowson, and Roger I. Glass. Burden of rotavirus disease among children visiting pediatric emergency departments in Cincinnati, Ohio, and Oakland, California, in 1999-2000. Pediatrics, 122(5):971–977, Nov 2008.

[69] M. H. Gorelick, K. N. Shaw, and K. O. Murphy. Validity and reliability of clinical signs in the diagnosis of dehydration in children. Pediatrics, 99(5):E6, 1997.

[70] S. B. Freedman, M. Adler, R. Seshadri, and E. C. Powell. Oral ondansetron for gastroenteritis in a pediatric emergency department. N Engl J Med, 354(16):1698–705, 2006.

[71] A. C. Plint, D. W. Johnson, H. Patel, N. Wiebe, R. Correll, R. Brant, C. Mitton, S. Gouin, M. Bhatt, G. Joubert, K. J. Black, T. Turner, S. Whitehouse, and T. P. Klassen. Epinephrine and dexamethasone in children with bronchiolitis. New England Journal of Medicine, 360(20):2079–89, 2009.

[72] N. Nagelkerke, V. Fidler, R. Bernsen, and M. Borgdorff. Estimating treatment effects in randomized clinical trials in the presence of non-compliance. Stat Med, 19(14):1849–1864, Jul 2000.

[73] J. Joensuu, E. Koskenniemi, X. L. Pang, and T. Vesikari. Randomised placebo-controlled trial of rhesus-human reassortant rotavirus vaccine for prevention of severe rotavirus gastroenteritis. Lancet, 350(9086):1205–1209, Oct 1997.

[74] Candan Cicek, Tuba Karatas, Imre Altuglu, Guldane Koturoglu, Zafer Kurugol, and Altinay Bilgic. Comparison of ELISA with shell vial cell culture method for the detection of human rotavirus in fecal specimens. New Microbiol, 30(2):113–118, Apr 2007.

[75] T. Vesikari, T. Ruuska, A. Delem, F. E. Andre, G. M. Beards, and T. H. Flewett.

E cacy of two doses of RIT 4237 bovine rotavirus vaccine for prevention of rotavirus diarrhoea. Acta Paediatr Scand, 80(2):173–80, 1991.

[76] Fred N. Binka, Francis K. Anto, Abraham R. Oduro, Elizabeth A. Awini, Alex K. Nazzar, George E. Armah, Richard H. Asmah, Andrew J. Hall, Felicity Cutts, Neal Alexander, David Brown, Jon Green, Jim Gray, Miren Iturriza-GÛmara, and Navrongo Rotavirus Research Group . Incidence and risk factors of paediatric rotavirus diarrhoea in northern Ghana. Trop Med Int Health, 8(9):840–846, Sep 2003.

[77] S. Holm. A simple sequentially rejective multiple test procedure. Scand J Stat, 6:65–70, 1979.

[78] P. Van Elteren. On the combination of independent two-sample tests of Wilcoxon. Bull Internat Stat Inst, 37:351–361, 1960.

[79] J Fleiss. Statistical Methods for Rates and Proportions. John Wiley & Sons, 2nd edition, 1981.

[80] H. M. Corneli, J. J. Zorc, P. Mahajan, K. N. Shaw, R. Holubkov, S. D. Reeves, R. M. Ruddy, B. Malik, K. A. Nelson, J. S. Bregstein, K. M. Brown, M. N. Denenberg, K. A. Lillis, L. B. Cimpello, J. W. Tsung, D. A. Borgialli, M. N. Baskin, G. Teshome, M. A. Goldstein, D. Monroe, J. M. Dean, and N. Kuppermann. A multicenter, randomized, controlled trial of dexamethasone for bronchiolitis. N Engl J Med, 357(4):331–9, 2007.

[81] M. Rosenblum and M. J. Van der Laan. Optimizing randomized trial designs to distinguish which subpopulations benefit from treatment. Biometrika, 98(4):845–860, 2011.

[82] W. J. Meurer, R. J. Lewis, and D. A. Berry. Adaptive clinical trials: a partial remedy for the therapeutic misconception? JAMA, 307(22):2377–8, 2012.

[83] D. Moher, S. Hopewell, K. F. Schulz, V. Montori, P. C. Gotzsche, P. J. Devereaux, D. Elbourne, M. Egger, and D. G. Altman. CONSORT 2010 explanation and elaboration: updated guidelines for reporting parallel group randomised trials. BMJ (Clinical Research Ed.), 340:c869, 2010.

[84] J. A. Lewis and D. Machin. Intention to treat–who should use ITT? Br J Cancer, 68(4):647–50, 1993.

Protocol 032 (Schnadower) Page 57 of 49

[85] M. Marshall, A. Lockwood, C. Bradley, C. Adams, C. Joy, and M. Fenton. Unpublished rating scales: a major source of bias in randomised controlled trials of treatments for schizophrenia. Br J Psychiatry, 176:249–52, 2000.

Protocol 032 (Schnadower) Page 58 of 49

[86] Eduardo Salazar-Lindo, Dante Figueroa-Quintanilla, Maria Isabel Caciano, Victoria Reto-Valiente, Gilles Chauviere, Patrick Colin, and Lacteol Study Group . Eﬀectiveness and safety of Lactobacillus LB in the treatment of mild acute diarrhea in children. J Pediatr Gastroenterol Nutr, 44(5):571–576, May 2007.

[87] N. Dendukuri, V. Costa, M. McGregor, and J. M. Brophy. Probiotic therapy for the prevention and treatment of Clostridium di cile-associated diarrhea: a systematic review. CMAJ, 173(2):167–70, 2005.

Probiotics Study Protocol Version 3.00
Protocol Version Date: February 13, 2017
The EMSC Pediatric Emergency Care Applied Research Network
