# Supplementary material for: Variables Associated With Intravenous Rehydration and Hospitalization in Children With Acute Gastroenteritis: A Secondary Analysis of 2 Randomized Clinical Trials
Source: JAMA Netw Open. 2021 Apr 19;4(4):e216433. doi: 10.1001/jamanetworkopen.2021.6433 (PMC8056281; doi:10.1001/jamanetworkopen.2021.6433)
Supplement: Supplement 3. — Statistical Analysis Plan [file jamanetwopen-e216433-s003.pdf]

PECARN Probiotics Trial
Factors associated with IV rehydration: Analysis Plan

Lead author: Freedman/Schnadower **Document author(s):** Cody Olsen, Charlie Casper

Version no: 3 **Version date:** 4/4/2019

STUDY DESIGN OVERVIEW

Study design: This is a secondary analysis of the PECARN Probiotics Trial and PROGUT Trial datasets. Details of those studies are included in protocols and the statistical analysis plan.

This manuscript will explore the association between IV rehydration during the enrollment ED visit and a list of potential risk factors. This is a cohort study design, as IV rehydration will likely be unaffected by the treatment, and the risk factors are measured prior to the outcome—IV rehydration use.

Research objectives and hypotheses:

- Objective 1: Describe IV rehydration in the ED and univariate associations between ED IV rehydration and patient characteristics/risk factors, including: clinical center, age, dehydration scale, infectious etiology, antiemetic use, SES, vomiting, diarrhea, and fever at presentation.

- Objective 2: Examine multivariable associations between ED IV rehydration and patient characteristics/risk factors.

- Objective 3: examine the relationship between IV fluids received and return visits.

Population definition:

Main study inclusion/exclusion criteria: See the study protocol(s).

Manuscript-specific criteria: All patients randomized into the Probiotics and PROGUT studies will be included. Because the outcome and risk factors are not collected from parental surveys, we will include patients who are lost-to-follow-up for the main study if baseline data are available. Patients for whom it is unknown whether IV fluids were given in the ED will be excluded from analyses.

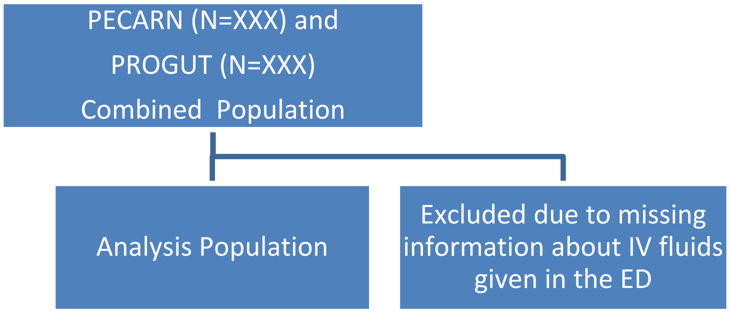

VARIABLES AND DEFINITIONS

Outcomes:

Primary outcome(s):

- IV rehydration: from the question: "Were any IV Fluids administered during the ED enrollment visit?" (day 14 chart review)

Secondary outcome(s):

- Hospitalization: Admitted to the hospital from the index ED (Yes/No)

Variables:

- Site: indicators for each clinical center/site.
- Dehydration scale: sum of the scores from general appearance, eyes, mucous membranes, and tears. This ranges from 0-8. A categorical version will be explored as a predictor of IV rehydration, and incorporated into a baseline severity score: None (0), Mild to Moderate (1-4), and Severe (5-8).
- Infectious etiology: these will be ascertained from PCR analysis of swab samples. Categories will be evaluated, and may be collapsed once initial results are available. We expect categories for isolated virus, isolated bacteria, virus/bacteria co-infection, c-diff alone, c-diff co-infection, other (including parasite), and none.
- Antiemetic use Yes/No: Yes if the patient received ondansetron (Zofran) during the ED enrollment visit. (Note that this may have been given after IV rehydration, possible through IV.)
- Socio-economic status: We will use the zip code to determine the median income for the zip code.
- Baseline duration of vomiting: We will use the MVS categories (0, 1-24 hrs, 25-48 hrs, 49+ hours), with the possibility of collapsing categories due to low prevalence in some.
- Number of vomiting episodes in the previous 24 hours: We will use the MVS categories that are defined for maximum vomiting episodes (0, 1, 2-4, 5+) with the possibility of collapsing categories due to low prevalence in some.
- Baseline duration of diarrhea: We will use the MVS categories (0, 1-96 hrs, 97-120 hrs, 121+ hrs) with the possibility of collapsing categories due to low prevalence in some.

- Number of diarrhea episodes in the previous 24 hours: We will use the MVS categories that are defined for maximum diarrhea episodes (0, 1-3, 4-5, 6+) with the possibility of collapsing categories due to low prevalence in some.
- Fever: Yes if the child has had a fever at any time for this illness; No otherwise (from the baseline information form).
- Maximum Temperature: maximum measured temperature from the baseline information form. When there was no fever, 37.0 will be used. When a fever was measured by tactile method only, this will be missing and not included in the analysis.
- Baseline Severity Score: We will calculate a score similar to the MVS by summing the diarrhea (duration and max/prior 24 hr), vomiting (duration and max/prior 24 hr), fever, and treatment components of the baseline MVS score. Additional points will be added based on dehydration: 0 points for dehydration score of 0, 2 points for a score 1-4, 3 points for a score 5-8. The unscheduled healthcare visit component will not be included. This will range from 0 to 20.
- Distance from the patient's home address to the hospital (km): use the zip/postal codes of the patients to calculate the geodetic distance to the hospital's zip code.

DATA ANALYSIS PLAN

Missing data / Imputation:

- We plan on using complete cases (those with no missing outcomes or predictors) for analyses. The outcome and predictors are taken from the baseline information collected in the ED, and not from the parent survey, so we don't expect much missing information. Imputation may be considered on a variable-by-variable basis as follows:

Data summary and analysis:

- Objective 1: Describe IV rehydration in the ED and univariate associations between ED IV rehydration and patient characteristics/risk factors

We will describe patient characteristics of those patients who received IV rehydration in the ED, and those who did not. Categorical measures will be summarized with counts and percentages, and compared between groups with Mantel-Haenzel tests stratified by clinical center. Continuous measures will be summarized using medians and inter-quartile-ranges (IQR: 25th percentile, 75th percentile) and compared between groups using Van Elteren's test stratified by clinical center.

	IV rehydration (N=XX)	No IV rehydration (N=XX)	P-value
Gender	X (%)	X (%)	0.999
Age	Mean (SD)	Mean (SD)	0.999

Dehydration Scale			
Infectious etiology			0.999
Rotavirus	X (%)	X (%)	
Norovirus	X (%)	X (%)	
Other virus…	X (%)	X (%)	
Bacteria…	X (%)	X (%)	
Parasite..	X (%)	X (%)	
Other/Unidentified	X (%)	X (%)	
Antiemetic given in the ED	X (%)	X (%)	0.999
Diarrhea Duration …			
No. of diarrhea episodes in previous 24 hours …			
Vomit Duration …			
No. of vomiting episodes in previous 24 hours …			
IV rehydration at healthcare visit prior to enrollment ED visit	X (%)	X (%)	0.999
Fever …			
Baseline Vomiting and Diarrhea Severity Score			

We will describe the variability of IV rehydration rates between centers. We could display the rates of IV rehydration by site with a barchart: x-axis is the de-identified site (labels are A-I, and sites are sorted by ascending rate of IV rehydration); y-axis is the percentage of patients who received IV rehydration.

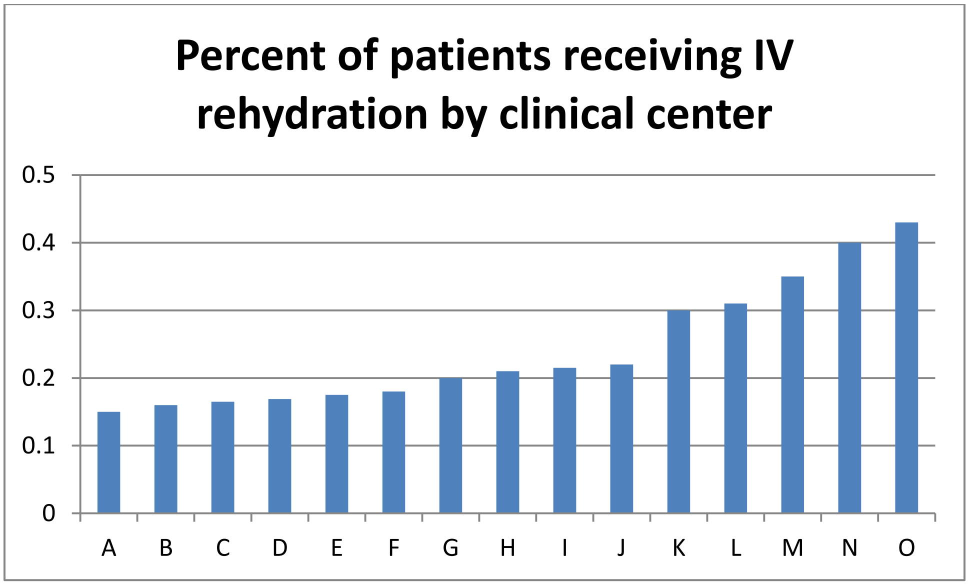

We will also report the min, max, median site IV rehydration rates.

Analyses will be repeated for the secondary outcome: hospitalization.

Objective 2: Examine multivariable associations between ED IV rehydration and patient characteristics/risk factors.

We will estimate multivariable associations between IV rehydration in the ED and risk factors using mixed logistic regression models. Mixed models will estimate for correlations within clinical centers. Although sites are nested within Country, Country will be treated as a fixed effect, and an odds-ratio will be estimated for it. We will also include the following risk factors as potential risk factors *a priori*: age group, dehydration score, and infectious etiology, antiemetic given in the ED, number of diarrhea episodes within 24 hours prior to enrollment number of vomiting episodes within 24 hours prior to enrollment, prior visit for this illness (no, yes without iv, yes with iv).

Table. Multivariable associations between IV rehydration and patient/country/site risk factors

	Risk Ratio (95% CI)	Multivariable P-value
Clinical Center (estimates not given)	N/A	0.999
Age	X.X (X.X, X.X)	0.999
Dehydration Scale	X.X (X.X, X.X)	0.999
Infectious etiology		0.999
Virus	X.X (X.X, X.X)	
Bacteria	X.X (X.X, X.X)	
Parasite	X.X (X.X, X.X)	
Other/Unidentified (Reference)	(Reference)	
Antiemetic given in the ED	X.X (X.X, X.X)	0.999

To show the effect of clinical centers, we could estimate and plot the risk of rehydration for each center at the mean/reference values of all other factors.

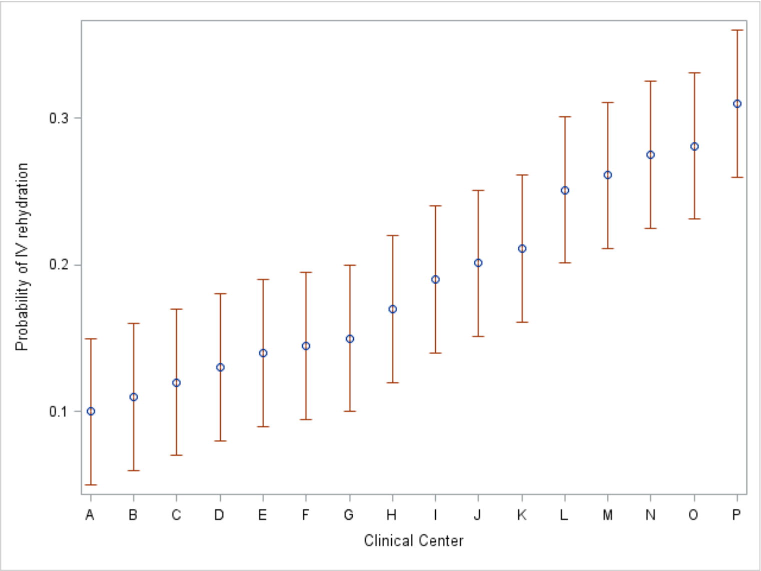

* Probability or risk, given the mean age (YY) and reference values for all risk factors (list those…).

Analyses will be repeated for the secondary outcome: hospitalization.

REFERENCES

[Provide numbered lists of any references utilized in this document (e.g., for variable definitions or in analysis plan.]

SUMMARY OF KEY REVISIONS

- We decided to NOT account for country specifically as a predictor.
    - We later decided to adjust for Country using a fixed effect, and Site using a random effect. We also decided to estimate an intra-class correlation with the site covariance parameter.
- I replaced Race and Ethnicity with Race/Ethnicity. Race is missing quite a bit, but combining with Ethnicity results in less missingness. (This is due to Unknown Race, but Hispanic Ethnicity results in Hispanic (not Unknown) Race/Ethnicity).
- 11.6.18 changes:
    - Race/Ethnicity is not collected by PROGUT Canadian study, so it is dropped from summaries and models.
    - Socieconomic status is dropped too because we don't have similar measures in Canada/US studies.
