# Supplementary material for: Variables Associated With Intravenous Rehydration and Hospitalization in Children With Acute Gastroenteritis: A Secondary Analysis of 2 Randomized Clinical Trials
Source: JAMA Netw Open. 2021 Apr 19;4(4):e216433. doi: 10.1001/jamanetworkopen.2021.6433 (PMC8056281; doi:10.1001/jamanetworkopen.2021.6433)
Supplement: Supplement 4. — Data Sharing Statement [file jamanetwopen-e216433-s004.pdf]

Data Sharing Statement

Poonai. Variables Associated With Intravenous Rehydration and Hospitalization in Children With Acute Gastroenteritis. *JAMA Netw Open*. Published April 19, 2021.
doi:10.1001/jamanetworkopen.2021.6433

Data
Data available: No
